# RuX: A Novel, Flexible, and Sensitive Mifepristone-Induced Transcriptional Regulation System

**DOI:** 10.1155/2023/7121512

**Published:** 2023-10-31

**Authors:** Anne Meinzinger, Áron Zsigmond, Péter Horváth, Alexandra Kellenberger, Katalin Paréj, Tiziano Tallone, Beáta Flachner, Marcell Cserhalmi, Zsolt Lőrincz, Sándor Cseh, Doron Shmerling

**Affiliations:** ^1^PolyGene AG, Rümlang, Switzerland; ^2^TargetEx Biosciences Ltd., Dunakeszi, Hungary; ^3^Department of Endocrinology, Metabolism and Cardiovascular Research, University of Fribourg, Fribourg, Switzerland

## Abstract

Inducible gene regulation methods are indispensable in diverse biological applications, yet many of them have severe limitations in their applicability. These include inducer toxicity, a limited variety of organisms the given system can be used in, and side effects of the induction method. In this study, a novel inducible system, the *RuX system*, was created using a mutant ligand-binding domain of the glucocorticoid receptor (CS1/CD), used together with various genetic elements such as the Gal4 DNA-binding domain or Cre recombinase. The RuX system is shown to be capable of over 1000-fold inducibility, has flexible applications, and is offered for use in cell cultures.

## 1. Introduction

Inducible gene regulation refers to a transcriptional regulation method that allows for artificial up- or downregulation of a heterologous or homologous gene of interest in either *in vitro* or *in vivo* models via administering or withdrawing a specific inducer, commonly a hormone or an antibiotic [1, 2]. In strict terms, the regulation is reversible, but the term is also used for irreversible, recombination-based gene activation.

Inducible animal models are primarily based on one of the tetracycline (tet) operon-related Tet systems or 4-hydroxytamoxifen- (4HT-) induced Cre-recombinase (Cre-ERT2, reviewed in [3]). There again, for cell and tissue cultures, a wide variety of systems is available (reviewed in [1, 2]). Synthetic biology approaches to create Boolean logic gates of genes have shown high flexibility of genetic networks in prokaryotes and the practical use of such systems as biosensors [4, 5]. Similar approaches have also emerged for eukaryotic organisms by utilizing CRISPR/Cas9 or inducible gene regulation [6, 7].

The available inducible systems have drawbacks. Several widespread inducible systems are known for inducer toxicity in mouse models. Doxycycline, the inducer of Tet, is reported for issues such as fetal loss, heart failure, or alteration of mitochondrial biochemical pathways [8–10]. 4HT, the Cre-ER-T2 inducer, is reported to be carcinogenic and harmful to the digestive system [11, 12]. Besides inducer toxicity, inducible gene regulation systems may have other limitations. The commercially available GeneSwitch system (Thermo Fisher Scientific Inc., Waltham, Massachusetts, USA; cat. no. K106001) is not recommended for use in the commonly used CHO cell line. Heavy metal- or heat-induced systems may introduce artefacts by inducing the general stress response pathways of the host organism [1]. This work is intended to address the aforementioned shortcomings by creating the RuX system: a flexible inducible gene regulator with low inducer toxicity, high inducibility, and various possible ways of utilization.

**Figure 1:**
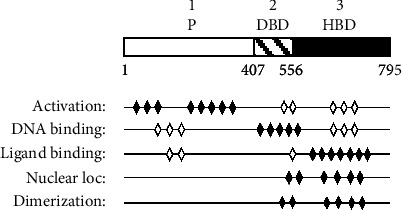
The modular structure of the rat glucocorticoid receptor (rGR). Scheme of the three major functional domains of the rGR. P denotes the potentiator domain, DBD is the DNA binding domain, and HBD is the hormone-binding domain. The operational borders of the domains are given by their amino acid positions below the rGR map (red numbers). Below: localization and quantitative estimation of the known major activities (listed at the left) of the rGR. The number and the pattern of the lozenges indicate the relative importance of the protein segment for a specific function (empty: minor; filled: primary). Abbreviations: bdg: binding; loc: localization.

The glucocorticoid receptor (GR) belongs to the large family of ligand-activated transcription factors [13, 14]. Limited proteolysis of purified rat GR [15] and the isolation of GR cDNA [16], together with extensive *in vitro* mutagenesis and functional analysis of chimeric proteins, suggested that the receptor can be separated into three distinct domains [17, 18]: (1) The N-terminal region (“potentiator”) contains the most potent transcriptional transactivation domains [17–19]. (2) The DNA-binding domain (DBD) is the segment necessary to dock the protein to the target sites on the DNA by binding the so-called glucocorticoid-responsive elements (GRE) [18]. First genetic [20] and then biophysical studies demonstrated that this domain consists of two zinc fingers [21, 22], reviewed in [18], of which the one closer to the N-terminus is primarily responsible for specific DNA recognition. In contrast, the second finger harbours several overlapping functions, such as unspecific DNA binding, dimerization, nuclear translocation, and also a weak transactivation function [18]. (3) The hormone-binding domain consists of about 300 amino acids and functions as a regulatory domain, transducing a simple chemical signal in a complex physiological answer [23]. It interacts with heat shock proteins, and it determines the intracellular compartmentalization of the GR by masking in the absence of ligands the nuclear-targeting sequences [24, 25]. A weak gene activation function [19, 26] was also detected in this segment.  Figure 1 illustrates the organization of the rat GR in three modules and shows that some important properties of the GR are “scattered” through the whole protein.

Strikingly, three decades ago, it was shown that the HBD of the GR acts as a transferable regulatory cassette that can confer hormonal control on chimeric proteins [27]. These results gave rise to the concept that this domain can function as a molecular switch to regulate the activity of unrelated linked proteins.

Nuclear receptors share a highly conserved region located at the very carboxy-terminal part of their ligand-binding domain (LBD). Point mutagenesis of conserved hydrophobic and charged residues in this region was reported to reduce ligand-dependent transcriptional activation but did not affect steroid or DNA binding [28]. Reiner B. Lanz and Sandro Rusconi constructed analogous mutations in the rat GR. They found a mutant LBD, called CS1/CD, strongly responsive to the antagonist ligand RU-486 (mifepristone) but unresponsive to the agonist dexamethasone (dex). This reversal of responsiveness was restricted to the rat GR since analogous mutations in related receptors do not generate similar phenotypes. Moreover, they found that, contrary to the progesterone receptor, carboxyl-end truncated GR mutants were not activated by RU-486 [29].

The CS1/CD LBD of the rat GR contains two substitutions (M770A, L771A—this was termed as “carboxy-terminal substitution 1” or CS1) and two deletions (P780Δ, K781Δ—this is “carboxy-terminal deletion” or CD). This mutant rat GR was highly responsive to RU-486 but not to the synthetic glucocorticoid dex, known to bind more powerfully to the GR than its endogenous agonist, cortisol, does [29]. Since this domain can be fused to heterologous polypeptides and its agonist is a pharmacological molecule (RU-486) not found in nature, it can be used as a “chemical switch” to regulate the activity of linked proteins and thus can be engineered into “gene switches” of various types.

In the past, other mutant steroid hormone-binding domains (SHBDs) were used to control the activity of linked proteins, such as the Cre recombinase. One well-known example is the Cre-ERT2 system based on a mutant estrogen receptor ligand-binding domain [30]. Alternatively, SHBDs can be used to generate inducible chimeric transcription factors by adding a DNA-binding domain (such as the DNA-binding domain of yeast Gal4 protein, Gal4 DBD) and a trans-regulatory domain (such as the herpes simplex virus VP16 domain) [29, 31].

**Figure 2:**
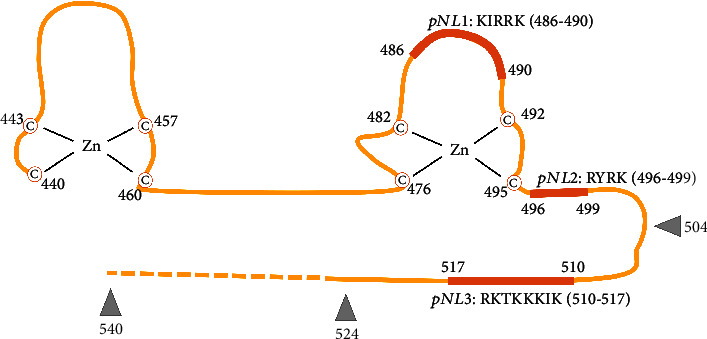
The main part of the DNA-binding region of the rGR starting with Cys440. Cysteines stabilizing the zinc fingers are labelled with the letter C in circles and their corresponding positions. Three identified protonuclear localization signals are shown with bold orange lines. The truncation positions are shown as grey arrowheads. This figure is based on Figure 1 of [44]. The original figure displays the protein between positions 440 and 525; the elongation added here is shown as a dashed line.

Developing a chimeric transcription factor from modules offers an opportunity to optimize each element one by one. The central part of the RuX system, the CS1/CD mutant of the GR^LBD^ domain, can be used starting from amino acid positions 504, 524, or 540 (see Figure 2). This involves the inclusion or exclusion of the protonuclear localization signal 3 (pNL3) and the transactivation domain tau-2 [17, 19]. Therefore, the behaviours of the chimeric transcription factors containing each of the truncated CS1/CD LBDs must be carefully evaluated to find the fragment that gives the best results.

A broad range of appropriate domains can be considered as a DNA-binding module. One such option, Gal4 DBD, is successfully established in different available systems [32, 33]. However, Gal4 DBD is also known to be nucleophilic, possibly causing noise levels in a chimeric transcription factor. Indeed, its nuclear localization signal could escape the “chemical switch control” of linked SHBDs. However, mutations that alter DNA binding, nuclear localization, or transcription characteristics of Gal4 DBD have been described, offering potential space for optimization [34, 35]. Proteins from the tetracycline repressor family (TetR family) can be considered for this role, too, such as macrolide 2′-phosphotransferase I (mphR(A)) [36] or HTH-type transcriptional regulator ttgR [37]. These proteins lose their DNA-binding ability in the presence of their respective inducers (erythromycin and phloretin).

The modular assembly allows for the exchange of the trans-regulatory domain. The most widespread transactivators are the VP16 and the p65 transactivation domains, used in several gene regulation systems [1]. Transrepression can be implemented via domains such as the Krüppel-Associated Box (KRAB), also successfully applied in inducible systems [38, 39].

Ultimately, these domains can be either tightly bound or connected via protein linker domains (see review [40]). In this work, we experimented with the latter, allowing for a choice of rigid or flexible protein linker domains.

We also developed and demonstrated here a novel inducible Cre molecule. The Cre/loxP system is an efficient recombinase machinery derived from the P1 bacteriophage. Cre recognizes loxP sites of 34 bp sequence length and catalyzes a recombination event between two sites. It is often used in genetic models to generate deletions, either causing abolition of gene expression or activating genes by deleting loxP-flanked transcriptional stop sequences; it can also cause translocations and inversions. An inducible Cre-ERT2 system, activated by tamoxifen binding to the mutated estrogen receptor ERT2, has been generated in the past [30]. The pitfalls of Cre-ERT2 include some models requiring subtoxic concentrations of tamoxifen to be applied to mice, hence causing considerable distress and side effects, as well as performance issues with high residual activity and questionable spontaneous induction effects [41]. This caused us to adopt the RuX methodology to assemble a RuX-Cre paradigm.

## 2. Materials and Methods

### 2.1. Cell Cultures Employed during Our Experiments

Parental Chinese hamster ovary cells (CHO-K1, ATCC: CRL-11268) were cultivated in F-12 Nut Mix (Ham) medium (Thermo Fisher Scientific Inc., ref. 21765-029) supplemented with 10% (*v*/*v*) heat-inactivated fetal bovine serum (FBS) (Euroclone S.p.A., cat. no. ECS0180L) and 1% (*v*/*v*) penicillin/streptomycin solution (Lonza, cat. no. DE17-602E). Heat inactivation of FBS was performed by incubation in 56°C water bath for 30 min.

Human embryonic kidney cells (HEK-293T, ATCC: CRL-11268) and HeLa cells (HeLa, ATCC: CCL-2) were cultivated in Dulbecco's modified Eagle medium (DMEM; Thermo Fisher Scientific Inc., ref. 11960-044) supplemented with 10% (*v*/*v*) heat-inactivated fetal bovine serum (FBS) (Euroclone S.p.A., cat. no. ECS0180L), 1% (*v*/*v*) L-Glutamine (Lonza, cat. no. 17-605E), and 1% (*v*/*v*) penicillin/streptomycin solution (Lonza, cat. no. DE17-602E).

All cell lines were cultivated in 75 mL cell culture flasks at 37°C in a humidified atmosphere containing 5% CO_2_. Subculture was performed every 3-4 days, at about 80% confluence. After 2x wash using Dulbecco's phosphate-buffered saline (DPBS) (Thermo Fisher Scientific Inc., ref. 14040-091), cells were detached from the surface of the culture flask by using a TrypLE Express reagent (Thermo Fisher Scientific Inc., ref. 12605-010). Viable cell numbers were determined using a TC20 Automated Cell Counter (Bio-Rad Laboratories, Inc.).

### 2.2. Plasmid Constructs Comprising the RuX System

Plasmids were generated via gene synthesis (Shanghai ShineGene Molecular Bio-Technologies, Inc.) and conventional cloning. Plasmid sequences are published as supplementary files (available here) in GenBank format. See Table 1 for all plasmids.

The GeneSwitch system (Thermo Fisher Scientific Inc.) was used to benchmark the RuX system. For the benchmarking, similar responder constructs were created using Gaussia luciferase (Gluc) [42] and NanoLuc-PEST (Nluc-P, obtained from Promega Corporation); all cloning in the GeneSwitch system was done according to the manufacturer's protocol.

### 2.3. Transfection Process

Monolayer cultures were transfected using an optimized polyethyleneimine- (PEI-) based protocol. Unless otherwise stated, 6 × 10^4^ (HEK, HeLa) or 5 × 10^4^ (CHO) cells were seeded in 0.5 ml medium per well of a 48-well plate (Greiner Bio-One Hungary Kft., cat. no. 677 180) and cultivated for 24 h. The culture medium was decanted before transfection and replaced with translucent DMEM/F-12 (Thermo Fisher Scientific Inc., ref. 11039-021) already containing the inducer (RU-486) (Sigma-Aldrich, cat. no. M8046-100MG) in the appropriate concentration.

The transfection was performed with a jetPEI transfection reagent (Polyplus Transfection, cat. no. 101-10N) according to the manufacturer's protocols: we diluted 1 *μ*l of jetPEI reagent in 25 *μ*l of 150 mM NaCl and added the solution to 25 *μ*l plasmid solution containing 500 ng DNA mix with 1 : 100-1 : 1 (regulator plasmid) : (responder plasmid) and 1 : 20 (ST00.1) : (responder plasmid) ratios, using pBS-SK as filler DNA. After vortexing and incubating at room temperature for 30 min, we added the mix to the wells.

After 24 and 48 hours, we took 150-150 *μ*l samples. After the second sampling, the plates were either discarded or used for Nluc-P assays.

### 2.4. Gaussia Luciferase Assays

The medium samples taken 48 hours after transfection were used for the Gaussia assays. 1 mM coelenterazine (Thermo Fisher Scientific Inc., USA; ref. C2944) stock solution was dissolved in a reaction buffer (25 mM Tris-HCL, 100 mM NaCl, pH = 7.5) to produce 0.2 *μ*M injection buffer. After transferring 50-50 *μ*l of the samples to a white 96-well plate (PerkinElmer Inc., Waltham, Massachusetts, USA; ref. 6005290), luminescence was measured on a Victor^2^ 1420 multilabel counter (PerkinElmer Inc.) right after injecting equal volumes of the injection buffer into the wells.

### 2.5. NanoLuc-PEST Luciferase Assays

At 48 hours, posttransfection cells were lysed with a lysis buffer made in our lab according to [43] (50 mM Tris-HCl, 150 mM NaCl, 2 mM EDTA, 1% (*v*/*v*) Nonidet P-40, pH 7.5). For the Nluc-P luciferase measurement, we prepared a reconstituted Nano-Glo luciferase assay reagent (Promega Corporation, Madison, Wisconsin, USA; ref. N1110) by combining one volume of the assay substrate with 50 volumes of the assay buffer according to the manufacturer's protocols. After transferring 50-50 *μ*l of the lysates to a white 96-well plate, luminescence was measured on a Victor^2^ 1420 multilabel counter three minutes after injecting equal volumes of the assay reagent into the wells.

### 2.6. SEAP Assays

Transfection control measurements were carried out with a SEAP reporter assay (Hoffmann-La Roche, ref. 11779842001) according to the manufacturer's protocols. After diluting samples 1 : 4 with the provided dilution buffer, we performed heat inactivation by incubating the plates for 30 min at 65°C. After transferring 25-25 *μ*l of the heat-inactivated samples, we added equal volumes of the inactivation buffer to the wells and incubated the plates for 5 min at RT. Luminescence was measured with a Victor^2^ 1420 multilabel counter after adding 25-25 *μ*l of the substrate reagent and incubating on a plate shaker at RT for 10 min.

### 2.7. Cytotoxicity Assays

3 × 10^4^ (HEK, HeLa) or 2.5 × 10^4^ (CHO) cells were seeded in 100 *μ*l medium per well of a 96-well plate (Greiner Bio-One Hungary Kft., cat. no. 655 180). After 24 hours, the culture medium was decanted and replaced with translucent DMEM/F-12 already containing the inducer in the appropriate concentrations. 48 hours after the addition of the inducer, the medium was exchanged to 10 *μ*l/well Alamar Blue reagent (BioSource Inc., ref. DAL1100). The plates were incubated on a shaker at RT for 10 min and then in a cell culture incubator at 37°C for 4 hours before measurement. Fluorescence measurement was carried out on a Victor^2^ 1420 multilabel counter.

### 2.8. Statistical Analysis of Data

Dose-response equations, curve fitting, data transformation, and descriptive statistics were calculated using the Prism software package (GraphPad Software).

## 3. Results and Discussion

### 3.1. Building a Flexible Testing System

#### 3.1.1. Universal Fusion Backbone

**Figure 3:**
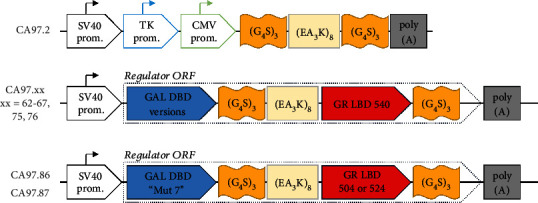
The structure of plasmid constructs CA97.2 (the base version of Universal Fusion Backbone) and the RuX activator plasmid family (CA97.62, CA97.63, CA97.64, CA97.65, CA97.66, CA97.67, CA97.75, CA97.76, CA97.86, and CA97.87) based on this construct. The base construct consists of three promoters and three linkers in-frame. Unique restriction sites (not shown) allow excising any two promoters and cloning cDNA of interest in between the linkers. Different Gal4 DNA-binding domain mutants (GAL DBD, blue box) were used in combination with different GR^LBD^ CS1/CD mutants (BR LBD, red box). Flexible linkers (“3x GGGS,” orange box) and a rigid linker (“8x EAAAK,” yellow box) are shown. The relative sizes of the elements are not proportional. Plasmid backbones are not shown.

To achieve a modular system with easy-to-exchange parts, a modular cloning vector CA97.2 was created. The most important features of CA97.2 are as follows (see also Figure 3):

- Three promoters (pSV40, pTK, and pCMV), any two of which can be easily collapsed in a single cloning step
- Linker sequences with unique restriction sites in between: the linkers used were a “flexible linker” (GGGGSGGGGSGGGGS) and a “rigid linker” (AEAAAKEAAAKEAAAKEAAAKALEAEAAAKEAAAKEAAAKEAAAKA) as suggested in [40]
- SV40 polyadenylation site

#### 3.1.2. Responder and Regulator Plasmids

**Figure 4:**
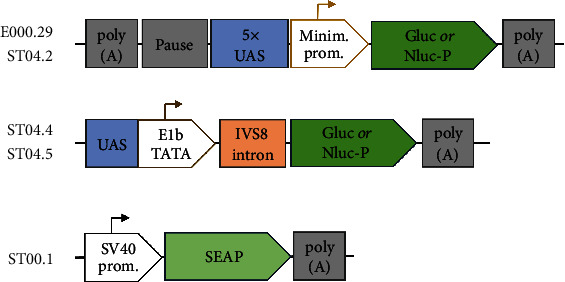
The structure of plasmid constructs E000.29, ST04.2 (Gluc and Nluc-P responder for RuX system), ST04.4 and ST04.5 (Gluc and Nluc-P responder for GeneSwitch system—recreated based on the protocol of the system), and the control plasmid ST00.1. The relative sizes of the elements are not proportional. Plasmid backbones are not shown.

To test the different versions of the RuX system and benchmark it to the GeneSwitch system, a series of responder plasmids (Figure 4) were created (E000.29 and ST04.2 for the RuX system and ST04.4 and ST04.5 for GeneSwitch). Gluc and Nluc-P were used as reporter genes. Although both systems aim to have a low baseline activity and excellent responsiveness, the exact builds of the two systems differ in many points. Unlike its counterpart, RuX responder plasmids feature an upstream polyadenylation signal and a pause site to reduce baseline activity. The regulated promoter in the RuX system consists of five copies of the Gal4 Upstream Activation Sequence (UAS) as described in [44] and a minimal promoter derived from the rabbit beta-globin gene [45].

A SEAP-expressing internal control plasmid (ST00.1) was created to serve as the overall transfection efficiency control.

### 3.2. Nucleophilicity of the RuX System

#### 3.2.1. Further Truncation of the Glucocorticoid Receptor

Although the mutations that change the inducer sensitivity of GR (thus making them relevant for the functional shift of the protein) are located only in the carboxy-terminal region (Cs1/CD), at least a part of the DNA-binding domain is also required for the nuclear shuttle. Based on the knowledge of the zinc fingers necessary for DNA binding and the protonuclear localization signals (pNL) present in this domain [46], a 3-step truncation was carried out to reduce the natural DNA binding of GR DBD (thus, the product does not bind the original binding sites in the genome), but leaving its ability to shuttle intact. The truncated parts (starting with amino acid positions 504, 524, and 540 of the full-length GR) are shown in Figure 2.

#### 3.2.2. Gal4 DBD with Reduced Nucleophilicity

To generate Gal4 DBDs with reduced nucleophilicity without affecting the DNA-binding capability, DNA-binding and nuclear localization regions were identified and modified. The following regions were considered based on data in the literature:

1. The bipartite NLS (R15-K23 and K43-R46) [47]
2. The DNA interaction surfaces (overlapping with the NLS) within the DBD (C11-K18 and C28-N35) [32]
3. The upstream DNA interaction surface, defining a stretch of 10 amino acid residues (C11-K20, overlapping with the DNA interaction surface) in which mutations disrupt Gal4 DNA-binding activity (termed as subregion A) [34]
4. Another stretch of 6 amino acid residues (C21-P26) (subregion B) [34]
5. Further mutations that affect nuclear localization but retain DNA-binding function: Y40H and K43E [35] as well as S6A, S22A, and S22D [33]

Considerations 1-4 suggest a 2-amino-acid stretch (S22-K23) that is part of the NLS and yet allows for modification: here, S22D, S22A, and K23Q were tested. Exchanges of lysins in subregion B that are not identified as parts of the NLS yet presumably contribute to the nucleophilicity of Gal4 DBD were also tested (K25F and K27S).

**Figure 5:**
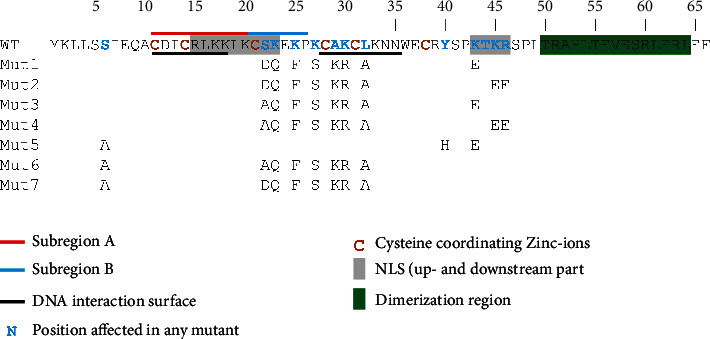
Wild-type and mutant (Mut1-Mut7) Gal4 DBD (1-66). Identified regions considered during mutagenesis are shown. In the mutants, only the substitutions are shown (for more details, see text).

Mutations based on consideration 5 were also tested in one or more mutants. Besides K43, the other lysin and the arginine in the downstream part of the NLS were also targeted in some of our mutants (K45E and R46E). Positions in the second DNA interaction surface (C28-N35) were changed in several of our mutants (A29K, K30R, and L32A). All suggested regions, subdomains, positions, and the seven mutants generated in this study are shown in Figure 5.

To identify which GR^LBD^ and Gal4 DBD mutant pairing gives the best signal/noise ratio, we performed preliminary Gaussia reporter measurements (Table 2). During the further development of the system, we used the GR^LBD^ 504-Gal4 DBD mutant 7 pairing, as the use of the chimeric protein based on these components resulted in the highest (1000x) fold change upon induction.

### 3.3. There Is No Observable Cross-Activation from Steroid Hormones at the Dose Sufficient for Half-Maximal Induction

**Figure 6:**
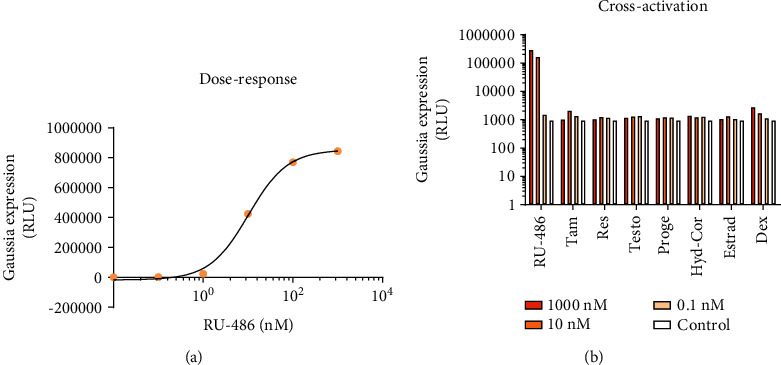
Dose-response and cross-induction measurements with steroid hormones.

The dose-response of the system was determined in a 10-fold dilution series over 6 steps. 50% of the maximum induction was reached at 10 nM (CI = 6.6 − 16.0) of the inducer (Figure 6). The mifepristone concentration required for induction is comparable to that of the GeneSwitch system (10 nM recommended by the manufacturer). To achieve robust induction, we used 50 nM inducer concentration in the benchmarking experiments.

After the determination of appropriate doses, possible instances of cross-induction by several steroid hormones (tamoxifen, resveratrol, testosterone, progesterone, hydrocortisol, estradiol, and dexamethasone) were examined in concentrations of 1000 nM, 10 nM, and 0.1 nM. The system shows some sensitivity towards dexamethasone at very high concentrations (at 1000 nM of inducer, 3-fold induction) (Figure 6).

### 3.4. Mifepristone Is Not Cytotoxic at the Concentrations Sufficient for Induction

**Figure 7:**
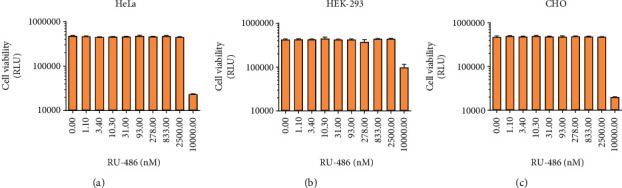
Imaging of Alamar Blue staining as an indicator of cytotoxicity (two biological replicate experiments, two parallels each, mean + SEM).

We have performed cytotoxicity measurements on all three cell lines used in this study with the inducer. We only observed cytotoxicity at approx. 1000x of the dose determined to cause half-maximal induction of the system (Figure 7).

### 3.5. Benchmarking the RuX System against the GeneSwitch System with the Use of Gaussia and NanoLuc-P Luciferase Reporters

We have compared the fold change in reporter expression levels following induction in our system and the commercially available GeneSwitch system. We measured several concentrations of activator plasmids and controls without responder plasmids.

**Figure 8:**
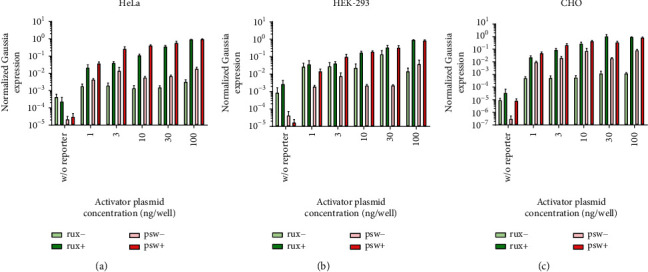
Gaussia luciferase expression levels in samples taken 48 hours after transfection from uninduced (rux-, psw-) and induced (rux+, psw+) cultures, normalized first with the respective SEAP expression levels as the transfection control, then between the datasets of each system to produce meaningful, comparable foldchange data (three biological replicate experiments, two parallels each, mean + SEM).

**Figure 9:**
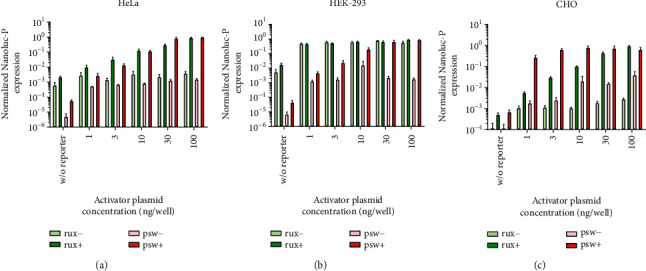
Nluc-P luciferase expression levels in samples taken 48 hours after transfection from uninduced (rux-, psw-) and induced (rux+, psw+) cultures, normalized first with the respective SEAP expression levels as the transfection control, then between the datasets of each system to produce meaningful, comparable foldchange data (three biological replicate experiments, two parallels each, mean + SEM).

The mifepristone-induced mean fold change in our system was comparable to that of the GeneSwitch system in HeLa cells, lower than that in HEK-293 cells, and higher than that in CHO cells, although there is some variation in the results between the two different reporter systems (Figures 8 and 9).

### 3.6. Construction of an Inducible, Non-Nuclear Cre System

To measure Cre action accurately against background levels, we constructed a reporter molecule that exchanges EGFP fluorescence for Gaussia luciferase activity. It is completely tight, i.e., it causes no Gaussia signal before induction (construct 0030.17).

**Figure 10:**
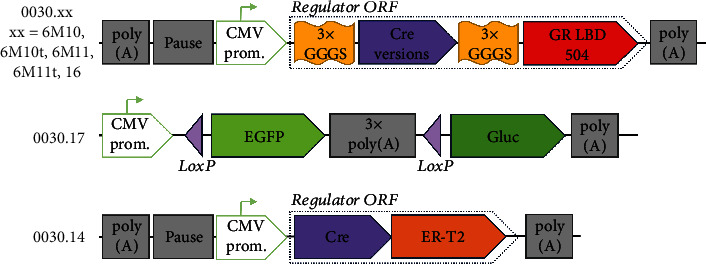
Structures of regulator and responder plasmids used for creating the Cre-RuX system. Regulator plasmids (0030.6, 0030.6M10, 0030.6M10t, 0030.6M11, 0030.6M11t, and 0030.16) express chimeric molecules composed of a fusion of different variations of a humanized Cre sequence and the GR^LBD^ CS1/CD 504 fragment. The 0030.17 responder plasmid consists of a CMV promoter-driven and floxed EGFP, polyadenylated by a strong artificial poly(A) element containing polyadenylation signals from the bovine globin gene and SV40 (“3x poly(A),” green box). This strong terminator completely blocks readthrough into the downstream Gluc. 0030.14 is a benchmarking plasmid containing the Cre-ERT2 system in the same structural setup as in the Cre-RuX constructs.

In a cyclical process of improving and mutating vectors containing point mutation and truncation variants of a humanized Cre sequence, along with nuclear localization elements (NLS) and various versions of CS1/CD as a chimeric molecule (base construct 0030.6; see Figure 10), we validated inducible Cre action upon mifepristone induction in the cell culture and benchmarked its action against an equivalent tamoxifen-inducible Cre-ERT2 construct (Table 3).

In a first step to adapt Cre functionality for this purpose, we decided to eliminate the putative NLS sequence of the Cre gene sequence. We identified six putative NLS amino acids: R100, R101, R118, R119, R121, and K122. Only one of them, amino acid R119, did not directly contact the DNA and was subjected to mutational testing. The mutants performing best were R119C (0030.6M10) and R119V (0030.6M11). Additionally, versions were made with amino-terminal deletions of 18 residues of the Cre protein in the NLS mutants 0030.6M10 and 0030.6M11. These versions were combined with different CS1/CD domain versions with and without protein linker bridges (Figure 10). Gaussia measurement revealed that both truncated NLS mutants, 0030.6M10t and 0030.6M11t, show a lower background activity compared to the Cre-ERT2 plasmid 0030.14, the NLS mutant 0030.6M11, or 0030.16 (containing a version of Cre with a V336A change proposed by Wunderlich in his 2004 PhD thesis [48]). Furthermore, we obtained higher induction levels than any of the other constructs at any concentration (Table 3).

## 4. Conclusions

We have developed a novel, robust gene regulation system, inducible by the progesterone analogue RU-486, the RuX system. The system is based on a heavily mutated ligand-binding domain of the rat glucocorticoid receptor, resulting in truncation, deletions, and amino acid substitutions. The system also features either a mutant version of the Gal4 DNA-binding domain (base system) or a mutant Cre (Cre-RuX system).

Our approach was the optimization of the global nucleophilicity of the fusion protein. During the development of these systems, we focused on altering the number and composition of the nucleophilic regions in all possible components. This concept appears to be a powerful tool for future development of similar transcriptional regulators or further enhancement of existing ones, for example, by changing the DNA-binding domain to other possible DNA-binding domains, such as TetR.

One condition that appears to affect the performance of such gene regulation systems is the cell line they are being used in. These cell lines are derived from different species and different organs and might have undergone different genetic modifications. It cannot be expected that the genetic elements introduced in these systems to keep transcriptional regulation in a certain window behave exactly in the same way. Therefore in this paper the RuX system was benchmarked against its direct competitor GeneSwitch in three commercially available mammalian cell lines: HeLa, HEK-293, and CHO. It is recommended that GeneSwitch not be used in one of the most popular cell lines, CHO cells, while the RuX system shows outstanding performance in these cells.

Similar to comparable hormones (dexamethasone, native glucocorticoid, etc.), mifepristone is a noncytotoxic agent in nanomolar concentrations and can even be applied in *μ*M concentration without showing toxicity, as observed in all three cell lines used in our experiments. Half-maximal induction was achieved at 10 nM mifepristone concentration. We used 50 nM inducer concentration in the benchmarking experiments to achieve robust induction while the operating adjustable concentrations were in the 2-50 nM range. The mifepristone-induced mean fold change in our system is comparable to that of the GeneSwitch system in HeLa cells, lower than that in HEK-293 cells, and higher than that in CHO cells, especially at higher concentrations.

Based on these results, our system is a suitable alternative or complementary tool for gene-expression studies in several mammalian cell lines, utilizing a nontoxic inducer. The modularity of the system means it is easily adaptable for each use case to achieve the highest possible signal-to-noise ratio between baseline expression and induction; however, a possible limitation of its use is the variability of the signal-to-noise ratio between cell lines. Another area to further investigate is the system's variable performance between reporter systems: this warrants further development of and experimentation with more reporter constructs. We definitely recommend testing the applicability of our system in each planned paradigm.

Besides utilization in cell culture, the RuX system can be considered in animal models as an alternative gene-regulating system to the Tet system or the Cre-ERT2. To prove this concept's validity *in vivo*, mouse models were created at PolyGene harbouring the RuX system. We are planning to test and optimize this next iteration of our product in the future to be able to offer a reliable, efficient gene regulation system for researchers working with the mouse model. However, as of now, we cannot demonstrate the applicability of our system in animal models of any kind: this constitutes the most significant limitation of our study. Extensive further investigation is warranted in this regard.

## Figures and Tables

**Table 1:** List of plasmid constructs created and used in the study (GS stands for GeneSwitch).

Plasmid name	Function	Remark
CA97.2	Base construct	Cloning backbone
CA97.67	RuX regulator	Wild-type Gal4 DBD × GR^LBD^ 540
CA97.62	RuX regulator	Gal4 DBD Mut1 × GR^LBD^ 540
CA97.63	RuX regulator	Gal4 DBD Mut2 × GR^LBD^ 540
CA97.64	RuX regulator	Gal4 DBD Mut3 × GR^LBD^ 540
CA97.65	RuX regulator	Gal4 DBD Mut4 × GR^LBD^ 540
CA97.66	RuX regulator	Gal4 DBD Mut5 × GR^LBD^ 540
CA97.75	RuX regulator	Gal4 DBD Mut6 × GR^LBD^ 540
CA97.76	RuX regulator	Gal4 DBD Mut7 × GR^LBD^ 540
CA97.86	RuX regulator	Gal4 DBD Mut7 × GR^LBD^ 524
CA97.87	RuX regulator	Gal4 DBD Mut7 × GR^LBD^ 504
E000.29	RuX responder	Testing RuX regulators, Gluc reporter
ST04.2	RuX responder	Testing RuX regulators, Nluc-P reporter
ST04.4	GS responder	Benchmarking GS, Gluc reporter
ST04.5	GS responder	Benchmarking GS, Nluc-P reporter
0030.6M10	RuX-Cre regulator	Cre R119C × GR^LBD^ 504
0030.6M11	RuX-Cre regulator	R119V × GR^LBD^ 504
0030.6M10t	RuX-Cre regulator	Cre R119C d(1-18) × GR^LBD^ 504
0030.6M11t	RuX-Cre inducer	Cre R119V d(1-18) × GR^LBD^ 504
0030.6	RuX-Cre regulator	huCre × GR^LBD^ 504
0030.16	RuX-Cre regulator	huCre V336A × GR^LBD^ 504
0030.14	Cre-ERT2 regulator	Cre-ERT2 adapted for testing against RuX-Cre
0030.17	Cre responder	EGFP expression switches to Gluc upon Cre
ST00.1	Internal control	Constitutive SEAP-expressing plasmid

**Table 2:** Fold-induction measurements with a Gaussia reporter of different (pre-) versions of the RuX system, in HeLa cells, 48 h posttransfection, compared to baseline right after transfection.

GR^LBD^	Gal4 DBD	Uninduced (log)	Induced (log)
540	WT	3	0.9
540	Mut1	—	—
540	Mut2	—	—
540	Mut3	—	—
540	Mut4	—	—
540	Mut5	—	—
540	Mut6	2	2.5
540	Mut7	1.2	2.5
524	Mut7	—	2.9
504	Mut7	—	3

**Table 3:** Performance benchmarking of Cre-RuX mutants against Cre-ERT2 HeLa cells, 48 h posttransfection, compared to baseline right after transfection. Induction rates are shown as relative Gaussia measurements normalized for transfection rates. The NLS-mutant 0030.6M011 has improved induction rates at any amount of plasmid transfected, compared to the best achievable rates with the Cre-ERT2 construct 0030.14. The base construct, 0030.6, is functional but outperformed by the improved versions 0030.6M10t and 0030.6M11t. The V336A mutant 0030.16 has high induction rates only at high transfection concentrations.


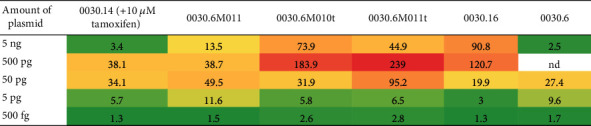

## Data Availability

The experimental data used to support the findings of this study are available from the corresponding author upon request. The plasmid sequences are listed in the supplementary material.

## Additional Points

*Patents*. The main elements of the induction system presented here have been filed for patent protection under EP 17703378.4.

## Supplementary Materials

Supplementary Materials The supplementary material file lists all plasmids used in our study in GenBank format.
