# Supplementary material for: RuX: A Novel, Flexible, and Sensitive Mifepristone-Induced Transcriptional Regulation System
Source: Int J Cell Biol. 2023 Oct 31;2023:7121512. doi: 10.1155/2023/7121512 (PMC10630016; doi:10.1155/2023/7121512)
Supplement: Supplementary Materials — The supplementary material file lists all plasmids used in our study in GenBank format. [file 7121512.f1.docx]

Supplementary: RuX: a novel, flexible and sensitive mifepristone-induced transcriptional regulation system

Anne Meinzinger ^#1^, Áron Zsigmond ^#2^, Péter Horváth ^1^, Alexandra Kellenberger ^1^, Katalin Paréj ^2^, Tiziano Tallone ^3^, Beáta Flachner ^2^, Marcell Cserhalmi ^2^, Zsolt Lőrincz ^2^, Sándor Cseh ^2^ and Doron Shmerling *^1^

^1^ PolyGene AG 1; Rümlang, Switzerland

^2^ TargetEx Biosciences Ltd.; Budapest, Hungary

^3^ Department of Endocrinology, Metabolism and Cardiovascular research, University of Fribourg; Fribourg, Switzerland

^#^ These authors have contributed equally to this work

* Correspondence: [doron.shmerling@polygene.ch](mailto:doron.shmerling@polygene.ch)

This supplementary material lists all plasmids used in our study in genbank format.

**0030.14:**

LOCUS 0030.14 4888 bp DNA circular SYN 08-JUL-2021

DEFINITION synthetic circular DNA

ACCESSION .

VERSION .

KEYWORDS .

SOURCE synthetic DNA construct

ORGANISM synthetic DNA construct

REFERENCE 1 (bases 1 to 4888)

AUTHORS .

TITLE .

JOURNAL .

FEATURES Location/Qualifiers

source 1..4888

/organism="synthetic DNA construct"

/mol_type="other DNA"

polyA_signal 102..236

/label=SV40 poly(A) signal

/note="SV40 polyadenylation signal"

rep_origin complement(425..1013)

/direction=LEFT

/label=ori

/note="high-copy-number ColE1/pMB1/pBR322/pUC origin of

replication"

CDS complement(1213..2073)

/codon_start=1

/product="beta-lactamase"

/label=AmpR

/note="confers resistance to ampicillin, carbenicillin,

and related antibiotics"

/translation="MSIQHFRVALIPFFAAFCLPVFAHPETLVKVKDAEDQLGARVGYI ELDLNSGKILESFRPEERFPMMSTFKVLLCGAVLSRIDAGQEQLGRRIHYSQNDLVEYS PVTEKHLTDGMTVRELCSAAITMSDNTAANLLLTTIGGPKELTAFLHNMGDHVTRLDRW EPELNEAIPNDERDTTMPVAMATTLRKLLTGELLTLASRQQLIDWMEADKVAGPLLRSA LPAGWFIADKSGAGERGSRGIIAALGPDGKPSRIVVIYTTGSQATMDERNRQIAEIGAS
LIKHW"

polyA_signal 2178..2226

/note="synthetic polyadenylation signal"

misc_feature 2240..2331

/label=pause site

/note="RNA polymerase II transcriptional pause signal from the human alpha-2 globin gene"

enhancer 2357..2660

/label=CMV enhancer

/note="human cytomegalovirus immediate early enhancer"

promoter 2661..2864

/label=CMV promoter

/note="human cytomegalovirus (CMV) immediate early

promoter"

CDS 2897..3925

/codon_start=1

/product="site-specific recombinase"

/label=Cre

/note="Cre is a site-specific recombinase from

bacteriophage P1. Recombination occurs at loxP

sequences."

/translation="MSNLLTVHQNLPALPVDATSDEVRKNLMDMFRDRQAFSEHTWKML LSVCRSWAAWCKLNNRKWFPAEPEDVRDYLLYLQARGLAVKTIQQHLGQLNMLHRRSGL PRPSDSNAVSLVMRRIRKENVDAGERAKQALAFERTDFDQVRSLMENSDRCQDIRNLAF LGIAYNTLLRIAEIARIRVKDISRTDGGRMLIHIGRTKTLVSTAGVEKALSLGVTKLVE RWISVSGVADDPNNYLFCRVRKNGVAAPSATSQLSTRALEGIFEATHRLIYGAKDDSGQ RYLAWSGHSARVGAARDMARAGVSIPEIMQAGGWTNVNIVMNYIRNLDSETGAMVRLLE
DGD"

CDS 3938..4873

/codon_start=1

/product="mutated ligand-binding domain of the human

estrogen receptor (Feil et al., 1997)"

/label=ERT2

/translation="AGDMRAANLWPSPLMIKRSKKNSLALSLTADQMVSALLDAEPPIL
YSEYDPTRPFSEASMMGLLTNLADRELVHMINWAKRVPGFVDLTLHDQVHLLECAWLEI LMIGLVWRSMEHPVKLLFAPNLLLDRNQGKCVEGMVEIFDMLLATSSRFRMMNLQGEEF VCLKSIILLNSGVYTFLSSTLKSLEEKDHIHRVLDKITDTLIHLMAKAGLTLQQQHQRL
AQLLLILSHIRHMSNKGMEHLYSMKCKNVVPLYDLLLEAADAHRLHAPTSRGGASVEET
DQSHLATAGSTSSHSLQKYYITGEAEGFPAT"

ORIGIN

1 cccagaggcg gccgctgaaa tcatcactaa tcagatatat tcaaatggaa atatcaaaaa

61 gcttctgttt catcagaagg ccggctaaac cggcaagctt cgatccagac atgataagat

121 acattgatga gtttggacaa accacaacta gaatgcagtg aaaaaaatgc tttatttgtg

181 aaatttgtga tgctattgct ttatttgtaa ccattataag ctgcaataaa caagttaaca

241 acaacaattg cattcatttt atgtttcagg ttcaggggga ggtgtgggag gttttttcgg

301 tatcagctca ctcaaaggcg gtaatacggt tatccacaga atcaggggat aacgcaggaa

361 agaacatgtg agcaaaaggc cagcaaaagg ccaggaaccg taaaaaggcc gcgttgctgg

421 cgtttttcca taggctccgc ccccctgacg agcatcacaa aaatcgacgc tcaagtcaga

481 ggtggcgaaa cccgacagga ctataaagat accaggcgtt tccccctgga agctccctcg

541 tgcgctctcc tgttccgacc ctgccgctta ccggatacct gtccgccttt ctcccttcgg

601 gaagcgtggc gctttctcat agctcacgct gtaggtatct cagttcggtg taggtcgttc

661 gctccaagct gggctgtgtg cacgaacccc ccgttcagcc cgaccgctgc gccttatccg

721 gtaactatcg tcttgagtcc aacccggtaa gacacgactt atcgccactg gcagcagcca

781 ctggtaacag gattagcaga gcgaggtatg taggcggtgc tacagagttc ttgaagtggt

841 ggcctaacta cggctacact agaagaacag tatttggtat ctgcgctctg ctgaagccag

901 ttaccttcgg aaaaagagtt ggtagctctt gatccggcaa acaaaccacc gctggtagcg

961 gtggtttttt tgtttgcaag cagcagatta cgcgcagaaa aaaaggatct caagaagatc

1021 ctttgatctt ttctacgggg tctgacgctc agtggaacga aaactcacgt taagggattt

1081 tggtcatgag attatcaaaa aggatcttca cctagatcct tttaaattaa aaatgaagtt

1141 ttaaatcaat ctaaagtata tatgagtaaa cttggtctga cagcggccgc aaatgctaaa

1201 ccactgcagt ggttaccagt gcttgatcag tgaggcaccg atctcagcga tctgcctatt

1261 tcgttcgtcc atagtggcct gactccccgt cgtgtagatc actacgattc gtgagggctt

1321 accatcaggc cccagcgcag caatgatgcc gcgagagccg cgttcaccgg cccccgattt

1381 gtcagcaatg aaccagccag cagggagggc cgagcgaaga agtggtcctg ctactttgtc

1441 cgcctccatc cagtctatga gctgctgtcg tgatgctaga gtaagaagtt cgccagtgag

1501 tagtttccga agagttgtgg ccattgctac tggcatcgtg gtatcacgct cgtcgttcgg

1561 tatggcttcg ttcaactctg gttcccagcg gtcaagccgg gtcacatgat cacccatatt

1621 atgaagaaat gcagtcagct ccttagggcc tccgatcgtt gtcagaagta agttggccgc

1681 ggtgttgtcg ctcatggtaa tggcagcact acacaattct cttaccgtca tgccatccgt

1741 aagatgcttt tccgtgaccg gcgagtactc aaccaagtcg ttttgtgagt agtgtatacg

1801 gcgaccaagc tgctcttgcc cggcgtctat acgggacaac accgcgccac atagcagtac

1861 tttgaaagtg ctcatcatcg ggaatcgttc ttcggggcgg aaagactcaa ggatcttgcc

1921 gctattgaga tccagttcga tatagcccac tcttgcaccc agttgatctt cagcatcttt

1981 tactttcacc agcgtttcgg ggtgtgcaaa aacaggcaag caaaatgccg caaagaaggg

2041 aatgagtgcg acacgaaaat gttggatgct catactcgtc ctttttcaat attattgaag

2101 catttatcag ggttactagt acgtctctca aggataagta agtaatatta aggtacggga

2161 ggtattggac aggccgcaat aaaatatctt tattttcatt acatctgtgt gttggttttt

2221 tgtgtgaatc gatagtacta acatacgctc tccatcaaaa caaaacgaaa caaaacaaac

2281 tagcaaaata ggctgtcccc agtgcaagtg caggtgccag aacatttctc tggcctaact

2341 ggccggtacc gagctccgtt acataactta cggtaaatgg cccgcctggc tgaccgccca

2401 acgacccccg cccattgacg tcaataatga cgtatgttcc catagtaacg ccaataggga

2461 ctttccattg acgtcaatgg gtggagtatt tacggtaaac tgcccacttg gcagtacatc

2521 aagtgtatca tatgccaagt acgcccccta ttgacgtcaa tgacggtaaa tggcccgcct

2581 ggcattatgc ccagtacatg accttatggg actttcctac ttggcagtac atctacgtat

2641 tagtcatcgc tattaccatg gtgatgcggt tttggcagta catcaatggg cgtggatagc

2701 ggtttgactc acggggattt ccaagtctcc accccattga cgtcaatggg agtttgtttt

2761 ggcaccaaaa tcaacgggac tttccaaaat gtcgtaacaa ctccgcccca ttgacgcaaa

2821 tgggcggtag gcgtgtacgg tgggaggtct atataagcag agctggttta gtgaaccgtc

2881 agatccgcta gcgaccatgt ccaatttact gaccgtacac caaaatttgc ctgcattacc

2941 ggtcgatgca acgagtgatg aggttcgcaa gaacctgatg gacatgttca gggatcgcca

3001 ggcgttttct gagcatacct ggaaaatgct tctgtccgtt tgccggtcgt gggcggcatg

3061 gtgcaagttg aataaccgga aatggtttcc cgcagaacct gaagatgttc gcgattatct

3121 tctatatctt caggcgcgcg gtctggcagt aaaaactatc cagcaacatt tgggccagct

3181 aaacatgctt catcgtcggt ccgggctgcc acgaccaagt gacagcaatg ctgtttcact

3241 ggttatgcgg cggatccgaa aagaaaacgt tgatgccggt gaacgtgcaa aacaggctct

3301 agcgttcgaa cgcactgatt tcgaccaggt tcgttcactc atggaaaata gcgatcgctg

3361 ccaggatata cgtaatctgg catttctggg gattgcttat aacaccctgt tacgtatagc

3421 cgaaattgcc aggatcaggg ttaaagatat ctcacgtact gacggtggga gaatgttaat

3481 ccatattggc agaacgaaaa cgctggttag caccgcaggt gtagagaagg cacttagcct

3541 gggggtaact aaactggtcg agcgatggat ttccgtctct ggtgtagctg atgatccgaa

3601 taactacctg ttttgccggg tcagaaaaaa tggtgttgcc gcgccatctg ccaccagcca

3661 gctatcaact cgcgccctgg aagggatttt tgaagcaact catcgattga tttacggcgc

3721 taaggatgac tctggtcaga gatacctggc ctggtctgga cacagtgccc gtgtcggagc

3781 cgcgcgagat atggcccgcg ctggagtttc aataccggag atcatgcaag ctggtggctg

3841 gaccaatgta aatattgtca tgaactatat ccgtaacctg gatagtgaaa caggggcaat

3901 ggtgcgcctg ctggaagatg gcgatctcga gccatctgct ggagacatga gagctgccaa

3961 cctttggcca agcccgctca tgatcaaacg ctctaagaag aacagcctgg ccttgtccct

4021 gacggccgac cagatggtca gtgccttgtt ggatgctgag ccccccatac tctattccga

4081 gtatgatcct accagaccct tcagtgaagc ttcgatgatg ggcttactga ccaacctggc

4141 agacagggag ctggttcaca tgatcaactg ggcgaagagg gtgccaggct ttgtggattt

4201 gaccctccat gatcaggtcc accttctaga atgtgcctgg ctagagatcc tgatgattgg

4261 tctcgtctgg cgctccatgg agcacccagt gaagctactg tttgctccta acttgctctt

4321 ggacaggaac cagggaaaat gtgtagaggg catggtggag atcttcgaca tgctgctggc

4381 tacatcatct cggttccgca tgatgaatct gcagggagag gagtttgtgt gcctcaaatc

4441 tattattttg cttaattctg gagtgtacac atttctgtcc agcaccctga agtctctgga

4501 agagaaggac catatccacc gagtcctgga caagatcaca gacactttga tccacctgat

4561 ggccaaggca ggcctgaccc tgcagcagca gcaccagcgg ctggcccagc tcctcctcat

4621 cctctcccac atcaggcaca tgagtaacaa aggcatggag catctgtaca gcatgaagtg

4681 caagaacgtg gtgcccctct atgacctgct gctggaggcg gcggacgccc accgcctaca

4741 tgcgcccact agccgtggag gggcatccgt ggaggagacg gaccaaagcc acttggccac

4801 tgcgggctct acttcatcgc attccttgca aaagtattac atcacggggg aggcagaggg

4861 tttccctgcc acagcttgat agcgaatt

//

**0030.16:**

LOCUS 0030.16 4926 bp DNA circular SYN 08-JUL-2021

DEFINITION synthetic circular DNA

ACCESSION .

VERSION .

KEYWORDS .

SOURCE synthetic DNA construct

ORGANISM synthetic DNA construct

REFERENCE 1 (bases 1 to 4926)

AUTHORS .

TITLE .

JOURNAL .

FEATURES Location/Qualifiers

source 1..4926

/organism="synthetic DNA construct"

/mol_type="other DNA"

enhancer 1..304

/label=CMV enhancer

/note="human cytomegalovirus immediate early enhancer"

promoter 305..508

/label=CMV promoter

/note="human cytomegalovirus (CMV) immediate early

promoter"

promoter 547..594

/label=EM7 promoter

/note="synthetic bacterial promoter "

CDS 619..663

/codon_start=1

/label=3x GGGGS

/translation="GGGGSGGGGSGGGGS"

CDS 673..1705

/codon_start=1

/label=huCre

/translation="MVSNLLTVHQNLPALPVDATSDEVRKNLMDMFRDRQAFSEHTWKM LLSVCRSWAAWCKLNNRKWFPAEPEDVRDYLLYLQARGLAVKTIQQHLGQLNMLHRRSG LPRPSDSNAVSLVMRRIRKENVDAGERAKQALAFERTDFDQVRSLMENSDRCQDIRNLA FLGIAYNTLLRIAEIARIRVKDISRTDGGRMLIHIGRTKTLVSTAGVEKALSLGVTKLV ERWISVSGVADDPNNYLFCRVRKNGVAAPSATSQLSTRALEGIFEATHRLIYGAKDDSG QRYLAWSGHSARVGAARDMARAGVSIPEIMQAGGWTNVNIVMNYIRNLDSETGAMARLL
EDGD"

CDS 1709..1753

/codon_start=1

/label=3x GGGGS

/translation="GEVAVVEADQVEAVV"

CDS 1777..2649

/codon_start=1

/label=GR LBD CS1/CD 504

/translation="AGMNLEARKTKKKIKGIQQATAGVSQDTSENPNKTIVPAALPQLT PTLVSLLEVIEPEVLYAGYDSSVPDSAWRIMTTLNMLGGRQVIAAVKWAKAIPGFRNLH LDDQMTLLQYSWMFLMAFALGWRSYRQSSGNLLCFAPDLIINEQRMSLPCMYDQCKHML FVSSELQRLQVSYEEYLCMKTLLLLSSVPKEGLKSQELFDEIRMTYIKELGKAIVKREG NSSQNWQRFYQLTKLLDSMHEVVENLLTYCFQTFLDKTMSIEFPEAAAEIITNQIYSNG
NIKKLLFHQK"

polyA_signal 2672..2806

/label=SV40 poly(A) signal

/note="SV40 polyadenylation signal"

rep_origin complement(2995..3583)

/direction=LEFT

/label=ori

/note="high-copy-number ColE1/pMB1/pBR322/pUC origin of

replication"

CDS complement(3783..4643)

/codon_start=1

/product="beta-lactamase"

/label=AmpR

/note="confers resistance to ampicillin, carbenicillin,

and related antibiotics"

/translation="MSIQHFRVALIPFFAAFCLPVFAHPETLVKVKDAEDQLGARVGYI ELDLNSGKILESFRPEERFPMMSTFKVLLCGAVLSRIDAGQEQLGRRIHYSQNDLVEYS PVTEKHLTDGMTVRELCSAAITMSDNTAANLLLTTIGGPKELTAFLHNMGDHVTRLDRW EPELNEAIPNDERDTTMPVAMATTLRKLLTGELLTLASRQQLIDWMEADKVAGPLLRSA LPAGWFIADKSGAGERGSRGIIAALGPDGKPSRIVVIYTTGSQATMDERNRQIAEIGAS
LIKHW"

polyA_signal 4748..4796

/note="synthetic polyadenylation signal"

misc_feature 4810..4901

/label=pause site

/note="RNA polymerase II transcriptional pause signal

from the human alpha-2 globin gene"

ORIGIN

1 cgttacataa cttacggtaa atggcccgcc tggctgaccg cccaacgacc cccgcccatt

61 gacgtcaata atgacgtatg ttcccatagt aacgccaata gggactttcc attgacgtca

121 atgggtggag tatttacggt aaactgccca cttggcagta catcaagtgt atcatatgcc

181 aagtacgccc cctattgacg tcaatgacgg taaatggccc gcctggcatt atgcccagta

241 catgacctta tgggactttc ctacttggca gtacatctac gtattagtca tcgctattac

301 catggtgatg cggttttggc agtacatcaa tgggcgtgga tagcggtttg actcacgggg

361 atttccaagt ctccacccca ttgacgtcaa tgggagtttg ttttggcacc aaaatcaacg

421 ggactttcca aaatgtcgta acaactccgc cccattgacg caaatgggcg gtaggcgtgt

481 acggtgggag gtctatataa gcagagctgg tttagtgaac cgtcagatcc gctagctcga

541 gcacgtgttg acaattaatc atcggcatag tatatcggca tagtataata cgacaaggtg

601 aggaactaaa ccatgtccgg aggcgggggc tcgggaggtg gcggctctgg aggaggcggt

661 agtaccggga ccatggtgag caacctgctg actgtgcacc agaacctgcc tgccctgcct

721 gtggatgcca cctctgatga ggtgaggaag aacctgatgg acatgttcag ggacaggcag

781 gccttctctg agcacacctg gaagatgctg ctgtctgtgt gcaggtcctg ggctgcctgg

841 tgcaagctga acaacaggaa gtggttccct gctgagccag aggatgtgag ggactacctg

901 ctgtacctgc aggccagagg cctggctgtg aagaccatcc agcagcacct gggccagctg

961 aacatgctcc acaggagatc tggcctgccc aggccctctg acagcaatgc tgtgagcctg

1021 gtgatgagga gaatcaggaa ggagaatgtg gatgctgggg agagggccaa gcaggccctg

1081 gcctttgaga ggactgactt tgaccaggtg aggagcctga tggagaacag tgacaggtgc

1141 caggacatca ggaacctggc cttcctgggc attgcctaca acaccctgct gaggattgct

1201 gagattgcca ggatcagggt gaaggacatc agcaggactg atggtggcag gatgctgatc

1261 cacattggca ggaccaagac cctggtgagc actgctgggg tggagaaggc cctgtccctg

1321 ggtgtgacca agctggtgga gaggtggatc tctgtgtctg gtgtggcaga tgaccccaac

1381 aactacctgt tctgcagggt gaggaagaat ggggtggctg ccccctctgc caccagtcag

1441 ctgagcacca gggccctgga gggcatcttt gaggccaccc acaggctgat ctatggggcc

1501 aaggatgaca gtggccagag gtatctggcc tggtctggcc actctgccag ggtgggggct

1561 gccagggaca tggccagggc tggtgtgagc atccctgaga tcatgcaggc tggtggctgg

1621 accaatgtga acattgtgat gaactacatc aggaacctgg acagtgagac tggtgccatg

1681 gcgaggctgc tggaggatgg ggacgccggg ggaggtggca gtggtggagg cggatcaggt

1741 ggaggcggta gtgccgggac catgttgggt acccctgcag gaatgaacct tgaagctcga

1801 aaaacaaaga aaaaaatcaa agggattcag caagccactg caggagtctc acaagacact

1861 tcggaaaatc ctaacaaaac aatagttcct gcagcattac cacagctcac ccctaccttg

1921 gtgtcactgc tggaggtgat tgaacccgag gtgttgtatg caggatatga tagctctgtt

1981 ccagattcag catggagaat tatgaccaca ctcaacatgt taggtgggcg tcaagtgatt

2041 gcagcagtga aatgggcaaa ggcgatacca ggcttcagaa acttacacct ggatgaccaa

2101 atgaccctgc tacagtactc atggatgttt ctcatggcat ttgccctggg ttggagatca

2161 tacagacaat caagtggaaa cctgctctgc tttgctcctg atctgattat taatgagcag

2221 agaatgtctc taccctgcat gtatgaccaa tgtaaacaca tgctgtttgt ctcctctgaa

2281 ttacaaagat tgcaggtatc ctatgaagag tatctctgta tgaaaacctt actgcttctc

2341 tcctcagttc ctaaggaagg tctgaagagc caagagttat ttgatgagat tcgaatgact

2401 tatatcaaag agctaggaaa agccatcgtc aaaagggaag ggaactccag tcagaactgg

2461 caacggtttt accaactgac aaagcttctg gactccatgc atgaggtggt tgagaatctc

2521 cttacctact gcttccagac atttttggat aagaccatga gtattgaatt cccagaggcg

2581 gccgctgaaa tcatcactaa tcagatatat tcaaatggaa atatcaaaaa gcttctgttt

2641 catcagaagg ccggctaaac cggcaagctt cgatccagac atgataagat acattgatga

2701 gtttggacaa accacaacta gaatgcagtg aaaaaaatgc tttatttgtg aaatttgtga

2761 tgctattgct ttatttgtaa ccattataag ctgcaataaa caagttaaca acaacaattg

2821 cattcatttt atgtttcagg ttcaggggga ggtgtgggag gttttttcgg tatcagctca

2881 ctcaaaggcg gtaatacggt tatccacaga atcaggggat aacgcaggaa agaacatgtg

2941 agcaaaaggc cagcaaaagg ccaggaaccg taaaaaggcc gcgttgctgg cgtttttcca

3001 taggctccgc ccccctgacg agcatcacaa aaatcgacgc tcaagtcaga ggtggcgaaa

3061 cccgacagga ctataaagat accaggcgtt tccccctgga agctccctcg tgcgctctcc

3121 tgttccgacc ctgccgctta ccggatacct gtccgccttt ctcccttcgg gaagcgtggc

3181 gctttctcat agctcacgct gtaggtatct cagttcggtg taggtcgttc gctccaagct

3241 gggctgtgtg cacgaacccc ccgttcagcc cgaccgctgc gccttatccg gtaactatcg

3301 tcttgagtcc aacccggtaa gacacgactt atcgccactg gcagcagcca ctggtaacag

3361 gattagcaga gcgaggtatg taggcggtgc tacagagttc ttgaagtggt ggcctaacta

3421 cggctacact agaagaacag tatttggtat ctgcgctctg ctgaagccag ttaccttcgg

3481 aaaaagagtt ggtagctctt gatccggcaa acaaaccacc gctggtagcg gtggtttttt

3541 tgtttgcaag cagcagatta cgcgcagaaa aaaaggatct caagaagatc ctttgatctt

3601 ttctacgggg tctgacgctc agtggaacga aaactcacgt taagggattt tggtcatgag

3661 attatcaaaa aggatcttca cctagatcct tttaaattaa aaatgaagtt ttaaatcaat

3721 ctaaagtata tatgagtaaa cttggtctga cagcggccgc aaatgctaaa ccactgcagt

3781 ggttaccagt gcttgatcag tgaggcaccg atctcagcga tctgcctatt tcgttcgtcc

3841 atagtggcct gactccccgt cgtgtagatc actacgattc gtgagggctt accatcaggc

3901 cccagcgcag caatgatgcc gcgagagccg cgttcaccgg cccccgattt gtcagcaatg

3961 aaccagccag cagggagggc cgagcgaaga agtggtcctg ctactttgtc cgcctccatc

4021 cagtctatga gctgctgtcg tgatgctaga gtaagaagtt cgccagtgag tagtttccga

4081 agagttgtgg ccattgctac tggcatcgtg gtatcacgct cgtcgttcgg tatggcttcg

4141 ttcaactctg gttcccagcg gtcaagccgg gtcacatgat cacccatatt atgaagaaat

4201 gcagtcagct ccttagggcc tccgatcgtt gtcagaagta agttggccgc ggtgttgtcg

4261 ctcatggtaa tggcagcact acacaattct cttaccgtca tgccatccgt aagatgcttt

4321 tccgtgaccg gcgagtactc aaccaagtcg ttttgtgagt agtgtatacg gcgaccaagc

4381 tgctcttgcc cggcgtctat acgggacaac accgcgccac atagcagtac tttgaaagtg

4441 ctcatcatcg ggaatcgttc ttcggggcgg aaagactcaa ggatcttgcc gctattgaga

4501 tccagttcga tatagcccac tcttgcaccc agttgatctt cagcatcttt tactttcacc

4561 agcgtttcgg ggtgtgcaaa aacaggcaag caaaatgccg caaagaaggg aatgagtgcg

4621 acacgaaaat gttggatgct catactcgtc ctttttcaat attattgaag catttatcag

4681 ggttactagt acgtctctca aggataagta agtaatatta aggtacggga ggtattggac

4741 aggccgcaat aaaatatctt tattttcatt acatctgtgt gttggttttt tgtgtgaatc

4801 gatagtacta acatacgctc tccatcaaaa caaaacgaaa caaaacaaac tagcaaaata

4861 ggctgtcccc agtgcaagtg caggtgccag aacatttctc tggcctaact ggccggtacc

4921 gagctc

//

**0030.6:**

LOCUS 0030.6 4926 bp DNA circular SYN 21-JUL-2021

DEFINITION synthetic circular DNA

ACCESSION .

VERSION .

KEYWORDS .

SOURCE synthetic DNA construct

ORGANISM synthetic DNA construct

REFERENCE 1 (bases 1 to 4926)

AUTHORS .

TITLE .

JOURNAL .

FEATURES Location/Qualifiers

source 1..4926

/organism="synthetic DNA construct"

/mol_type="other DNA"

enhancer 1..304

/label=CMV enhancer

/note="human cytomegalovirus immediate early enhancer"

promoter 305..508

/label=CMV promoter

/note="human cytomegalovirus (CMV) immediate early

promoter"

promoter 547..594

/label=EM7 promoter

/note="synthetic bacterial promoter "

CDS 619..663

/codon_start=1

/label=3x GGGGS

/translation="GGGGSGGGGSGGGGS"

CDS 673..1705

/codon_start=1

/label=huCre

/translation="MVSNLLTVHQNLPALPVDATSDEVRKNLMDMFRDRQAFSEHTWKM LLSVCRSWAAWCKLNNRKWFPAEPEDVRDYLLYLQARGLAVKTIQQHLGQLNMLHRRSG LPRPSDSNAVSLVMRRIRKENVDAGERAKQALAFERTDFDQVRSLMENSDRCQDIRNLA FLGIAYNTLLRIAEIARIRVKDISRTDGGRMLIHIGRTKTLVSTAGVEKALSLGVTKLV ERWISVSGVADDPNNYLFCRVRKNGVAAPSATSQLSTRALEGIFEATHRLIYGAKDDSG QRYLAWSGHSARVGAARDMARAGVSIPEIMQAGGWTNVNIVMNYIRNLDSETGAMVRLL
EDGD"

CDS 1709..1753

/codon_start=1

/label=3x GGGGS

/translation="GEVAVVEADQVEAVV"

CDS 1774..2566

/codon_start=1

/label=GR LBD 504

/translation="PAGMNLEARKTKKKIKGIQQATAGVSQDTSENPNKTIVPAALPQL
TPTLVSLLEVIEPEVLYAGYDSSVPDSAWRIMTTLNMLGGRQVIAAVKWAKAIPGFRNL HLDDQMTLLQYSWMFLMAFALGWRSYRQSSGNLLCFAPDLIINEQRMSLPCMYDQCKHM LFVSSELQRLQVSYEEYLCMKTLLLLSSVPKEGLKSQELFDEIRMTYIKELGKAIVKRE
GNSSQNWQRFYQLTKLLDSMHEVVENLLTYCFQTFLDKTMSI"

polyA_signal 2672..2806

/label=SV40 poly(A) signal

/note="SV40 polyadenylation signal"

rep_origin complement(2995..3583)

/direction=LEFT

/label=ori

/note="high-copy-number ColE1/pMB1/pBR322/pUC origin of

replication"

CDS complement(3783..4643)

/codon_start=1

/product="beta-lactamase"

/label=AmpR

/note="confers resistance to ampicillin, carbenicillin, and related antibiotics"

/translation="MSIQHFRVALIPFFAAFCLPVFAHPETLVKVKDAEDQLGARVGYI ELDLNSGKILESFRPEERFPMMSTFKVLLCGAVLSRIDAGQEQLGRRIHYSQNDLVEYS PVTEKHLTDGMTVRELCSAAITMSDNTAANLLLTTIGGPKELTAFLHNMGDHVTRLDRW EPELNEAIPNDERDTTMPVAMATTLRKLLTGELLTLASRQQLIDWMEADKVAGPLLRSA LPAGWFIADKSGAGERGSRGIIAALGPDGKPSRIVVIYTTGSQATMDERNRQIAEIGAS
LIKHW"

polyA_signal 4748..4796

/note="synthetic polyadenylation signal"

misc_feature 4810..4901

/label=pause site

/note="RNA polymerase II transcriptional pause signal from the human alpha-2 globin gene"

ORIGIN

1 cgttacataa cttacggtaa atggcccgcc tggctgaccg cccaacgacc cccgcccatt

61 gacgtcaata atgacgtatg ttcccatagt aacgccaata gggactttcc attgacgtca

121 atgggtggag tatttacggt aaactgccca cttggcagta catcaagtgt atcatatgcc

181 aagtacgccc cctattgacg tcaatgacgg taaatggccc gcctggcatt atgcccagta

241 catgacctta tgggactttc ctacttggca gtacatctac gtattagtca tcgctattac

301 catggtgatg cggttttggc agtacatcaa tgggcgtgga tagcggtttg actcacgggg

361 atttccaagt ctccacccca ttgacgtcaa tgggagtttg ttttggcacc aaaatcaacg

421 ggactttcca aaatgtcgta acaactccgc cccattgacg caaatgggcg gtaggcgtgt

481 acggtgggag gtctatataa gcagagctgg tttagtgaac cgtcagatcc gctagctcga

541 gcacgtgttg acaattaatc atcggcatag tatatcggca tagtataata cgacaaggtg

601 aggaactaaa ccatgtccgg aggcgggggc tcgggaggtg gcggctctgg aggaggcggt

661 agtaccggga ccatggtgag caacctgctg actgtgcacc agaacctgcc tgccctgcct

721 gtggatgcca cctctgatga ggtgaggaag aacctgatgg acatgttcag ggacaggcag

781 gccttctctg agcacacctg gaagatgctg ctgtctgtgt gcaggtcctg ggctgcctgg

841 tgcaagctga acaacaggaa gtggttccct gctgagccag aggatgtgag ggactacctg

901 ctgtacctgc aggccagagg cctggctgtg aagaccatcc agcagcacct gggccagctg

961 aacatgctcc acaggagatc tggcctgccc aggccctctg acagcaatgc tgtgagcctg

1021 gtgatgagga gaatcaggaa ggagaatgtg gatgctgggg agagggccaa gcaggccctg

1081 gcctttgaga ggactgactt tgaccaggtg aggagcctga tggagaacag tgacaggtgc

1141 caggacatca ggaacctggc cttcctgggc attgcctaca acaccctgct gaggattgct

1201 gagattgcca ggatcagggt gaaggacatc agcaggactg atggtggcag gatgctgatc

1261 cacattggca ggaccaagac cctggtgagc actgctgggg tggagaaggc cctgtccctg

1321 ggtgtgacca agctggtgga gaggtggatc tctgtgtctg gtgtggcaga tgaccccaac

1381 aactacctgt tctgcagggt gaggaagaat ggggtggctg ccccctctgc caccagtcag

1441 ctgagcacca gggccctgga gggcatcttt gaggccaccc acaggctgat ctatggggcc

1501 aaggatgaca gtggccagag gtatctggcc tggtctggcc actctgccag ggtgggggct

1561 gccagggaca tggccagggc tggtgtgagc atccctgaga tcatgcaggc tggtggctgg

1621 accaatgtga acattgtgat gaactacatc aggaacctgg acagtgagac tggtgccatg

1681 gtgaggctgc tggaggatgg ggacgccggg ggaggtggca gtggtggagg cggatcaggt

1741 ggaggcggta gtgccgggac catgttgggt acccctgcag gaatgaacct tgaagctcga

1801 aaaacaaaga aaaaaatcaa agggattcag caagccactg caggagtctc acaagacact

1861 tcggaaaatc ctaacaaaac aatagttcct gcagcattac cacagctcac ccctaccttg

1921 gtgtcactgc tggaggtgat tgaacccgag gtgttgtatg caggatatga tagctctgtt

1981 ccagattcag catggagaat tatgaccaca ctcaacatgt taggtgggcg tcaagtgatt

2041 gcagcagtga aatgggcaaa ggcgatacca ggcttcagaa acttacacct ggatgaccaa

2101 atgaccctgc tacagtactc atggatgttt ctcatggcat ttgccctggg ttggagatca

2161 tacagacaat caagtggaaa cctgctctgc tttgctcctg atctgattat taatgagcag

2221 agaatgtctc taccctgcat gtatgaccaa tgtaaacaca tgctgtttgt ctcctctgaa

2281 ttacaaagat tgcaggtatc ctatgaagag tatctctgta tgaaaacctt actgcttctc

2341 tcctcagttc ctaaggaagg tctgaagagc caagagttat ttgatgagat tcgaatgact

2401 tatatcaaag agctaggaaa agccatcgtc aaaagggaag ggaactccag tcagaactgg

2461 caacggtttt accaactgac aaagcttctg gactccatgc atgaggtggt tgagaatctc

2521 cttacctact gcttccagac atttttggat aagaccatga gtattgaatt cccagaggcg

2581 gccgctgaaa tcatcactaa tcagatatat tcaaatggaa atatcaaaaa gcttctgttt

2641 catcagaagg ccggctaaac cggcaagctt cgatccagac atgataagat acattgatga

2701 gtttggacaa accacaacta gaatgcagtg aaaaaaatgc tttatttgtg aaatttgtga

2761 tgctattgct ttatttgtaa ccattataag ctgcaataaa caagttaaca acaacaattg

2821 cattcatttt atgtttcagg ttcaggggga ggtgtgggag gttttttcgg tatcagctca

2881 ctcaaaggcg gtaatacggt tatccacaga atcaggggat aacgcaggaa agaacatgtg

2941 agcaaaaggc cagcaaaagg ccaggaaccg taaaaaggcc gcgttgctgg cgtttttcca

3001 taggctccgc ccccctgacg agcatcacaa aaatcgacgc tcaagtcaga ggtggcgaaa

3061 cccgacagga ctataaagat accaggcgtt tccccctgga agctccctcg tgcgctctcc

3121 tgttccgacc ctgccgctta ccggatacct gtccgccttt ctcccttcgg gaagcgtggc

3181 gctttctcat agctcacgct gtaggtatct cagttcggtg taggtcgttc gctccaagct

3241 gggctgtgtg cacgaacccc ccgttcagcc cgaccgctgc gccttatccg gtaactatcg

3301 tcttgagtcc aacccggtaa gacacgactt atcgccactg gcagcagcca ctggtaacag

3361 gattagcaga gcgaggtatg taggcggtgc tacagagttc ttgaagtggt ggcctaacta

3421 cggctacact agaagaacag tatttggtat ctgcgctctg ctgaagccag ttaccttcgg

3481 aaaaagagtt ggtagctctt gatccggcaa acaaaccacc gctggtagcg gtggtttttt

3541 tgtttgcaag cagcagatta cgcgcagaaa aaaaggatct caagaagatc ctttgatctt

3601 ttctacgggg tctgacgctc agtggaacga aaactcacgt taagggattt tggtcatgag

3661 attatcaaaa aggatcttca cctagatcct tttaaattaa aaatgaagtt ttaaatcaat

3721 ctaaagtata tatgagtaaa cttggtctga cagcggccgc aaatgctaaa ccactgcagt

3781 ggttaccagt gcttgatcag tgaggcaccg atctcagcga tctgcctatt tcgttcgtcc

3841 atagtggcct gactccccgt cgtgtagatc actacgattc gtgagggctt accatcaggc

3901 cccagcgcag caatgatgcc gcgagagccg cgttcaccgg cccccgattt gtcagcaatg

3961 aaccagccag cagggagggc cgagcgaaga agtggtcctg ctactttgtc cgcctccatc

4021 cagtctatga gctgctgtcg tgatgctaga gtaagaagtt cgccagtgag tagtttccga

4081 agagttgtgg ccattgctac tggcatcgtg gtatcacgct cgtcgttcgg tatggcttcg

4141 ttcaactctg gttcccagcg gtcaagccgg gtcacatgat cacccatatt atgaagaaat

4201 gcagtcagct ccttagggcc tccgatcgtt gtcagaagta agttggccgc ggtgttgtcg

4261 ctcatggtaa tggcagcact acacaattct cttaccgtca tgccatccgt aagatgcttt

4321 tccgtgaccg gcgagtactc aaccaagtcg ttttgtgagt agtgtatacg gcgaccaagc

4381 tgctcttgcc cggcgtctat acgggacaac accgcgccac atagcagtac tttgaaagtg

4441 ctcatcatcg ggaatcgttc ttcggggcgg aaagactcaa ggatcttgcc gctattgaga

4501 tccagttcga tatagcccac tcttgcaccc agttgatctt cagcatcttt tactttcacc

4561 agcgtttcgg ggtgtgcaaa aacaggcaag caaaatgccg caaagaaggg aatgagtgcg

4621 acacgaaaat gttggatgct catactcgtc ctttttcaat attattgaag catttatcag

4681 ggttactagt acgtctctca aggataagta agtaatatta aggtacggga ggtattggac

4741 aggccgcaat aaaatatctt tattttcatt acatctgtgt gttggttttt tgtgtgaatc

4801 gatagtacta acatacgctc tccatcaaaa caaaacgaaa caaaacaaac tagcaaaata

4861 ggctgtcccc agtgcaagtg caggtgccag aacatttctc tggcctaact ggccggtacc

4921 gagctc

//

**0030.6M10:**

LOCUS 0030.6M10 4926 bp DNA circular SYN 08-JUL-2021

DEFINITION synthetic circular DNA

ACCESSION .

VERSION .

KEYWORDS .

SOURCE synthetic DNA construct

ORGANISM synthetic DNA construct

REFERENCE 1 (bases 1 to 4926)

AUTHORS .

TITLE .

JOURNAL .

FEATURES Location/Qualifiers

source 1..4926

/organism="synthetic DNA construct"

/mol_type="other DNA"

enhancer 1..304

/label=CMV enhancer

/note="human cytomegalovirus immediate early enhancer"

promoter 305..508

/label=CMV promoter

/note="human cytomegalovirus (CMV) immediate early

promoter"

promoter 547..594

/label=EM7 promoter

/note="synthetic bacterial promoter "

CDS 619..663

/codon_start=1

/label=3x GGGGS

/translation="GGGGSGGGGSGGGGS"

CDS 673..1705

/codon_start=1

/label=huCre R119C

/translation="MVSNLLTVHQNLPALPVDATSDEVRKNLMDMFRDRQAFSEHTWKM LLSVCRSWAAWCKLNNRKWFPAEPEDVRDYLLYLQARGLAVKTIQQHLGQLNMLHRRSG LPRPSDSNAVSLVMRCIRKENVDAGERAKQALAFERTDFDQVRSLMENSDRCQDIRNLA FLGIAYNTLLRIAEIARIRVKDISRTDGGRMLIHIGRTKTLVSTAGVEKALSLGVTKLV ERWISVSGVADDPNNYLFCRVRKNGVAAPSATSQLSTRALEGIFEATHRLIYGAKDDSG QRYLAWSGHSARVGAARDMARAGVSIPEIMQAGGWTNVNIVMNYIRNLDSETGAMVRLL
EDGD"

CDS 1709..1753

/codon_start=1

/label=3x GGGGS

/translation="GEVAVVEADQVEAVV"

CDS 1777..2649

/codon_start=1

/label=GR LBD CS1/CD 504

/translation="AGMNLEARKTKKKIKGIQQATAGVSQDTSENPNKTIVPAALPQLT PTLVSLLEVIEPEVLYAGYDSSVPDSAWRIMTTLNMLGGRQVIAAVKWAKAIPGFRNLH LDDQMTLLQYSWMFLMAFALGWRSYRQSSGNLLCFAPDLIINEQRMSLPCMYDQCKHML FVSSELQRLQVSYEEYLCMKTLLLLSSVPKEGLKSQELFDEIRMTYIKELGKAIVKREG NSSQNWQRFYQLTKLLDSMHEVVENLLTYCFQTFLDKTMSIEFPEAAAEIITNQIYSNG
NIKKLLFHQK"

polyA_signal 2672..2806

/label=SV40 poly(A) signal

/note="SV40 polyadenylation signal"

rep_origin complement(2995..3583)

/direction=LEFT

/label=ori

/note="high-copy-number ColE1/pMB1/pBR322/pUC origin of

replication"

CDS complement(3783..4643)

/codon_start=1

/product="beta-lactamase"

/label=AmpR

/note="confers resistance to ampicillin, carbenicillin, and related antibiotics"

/translation="MSIQHFRVALIPFFAAFCLPVFAHPETLVKVKDAEDQLGARVGYI ELDLNSGKILESFRPEERFPMMSTFKVLLCGAVLSRIDAGQEQLGRRIHYSQNDLVEYS PVTEKHLTDGMTVRELCSAAITMSDNTAANLLLTTIGGPKELTAFLHNMGDHVTRLDRW EPELNEAIPNDERDTTMPVAMATTLRKLLTGELLTLASRQQLIDWMEADKVAGPLLRSA LPAGWFIADKSGAGERGSRGIIAALGPDGKPSRIVVIYTTGSQATMDERNRQIAEIGAS
LIKHW"

polyA_signal 4748..4796

/note="synthetic polyadenylation signal"

misc_feature 4810..4901

/label=pause site

/note="RNA polymerase II transcriptional pause signal from the human alpha-2 globin gene"

ORIGIN

1 cgttacataa cttacggtaa atggcccgcc tggctgaccg cccaacgacc cccgcccatt

61 gacgtcaata atgacgtatg ttcccatagt aacgccaata gggactttcc attgacgtca

121 atgggtggag tatttacggt aaactgccca cttggcagta catcaagtgt atcatatgcc

181 aagtacgccc cctattgacg tcaatgacgg taaatggccc gcctggcatt atgcccagta

241 catgacctta tgggactttc ctacttggca gtacatctac gtattagtca tcgctattac

301 catggtgatg cggttttggc agtacatcaa tgggcgtgga tagcggtttg actcacgggg

361 atttccaagt ctccacccca ttgacgtcaa tgggagtttg ttttggcacc aaaatcaacg

421 ggactttcca aaatgtcgta acaactccgc cccattgacg caaatgggcg gtaggcgtgt

481 acggtgggag gtctatataa gcagagctgg tttagtgaac cgtcagatcc gctagctcga

541 gcacgtgttg acaattaatc atcggcatag tatatcggca tagtataata cgacaaggtg

601 aggaactaaa ccatgtccgg aggcgggggc tcgggaggtg gcggctctgg aggaggcggt

661 agtaccggga ccatggtgag caacctgctg actgtgcacc agaacctgcc tgccctgcct

721 gtggatgcca cctctgatga ggtgaggaag aacctgatgg acatgttcag ggacaggcag

781 gccttctctg agcacacctg gaagatgctg ctgtctgtgt gcaggtcctg ggctgcctgg

841 tgcaagctga acaacaggaa gtggttccct gctgagccag aggatgtgag ggactacctg

901 ctgtacctgc aggccagagg cctggctgtg aagaccatcc agcagcacct gggccagctg

961 aacatgctcc acaggagatc tggcctgccc aggccctctg acagcaatgc tgtgagcctg

1021 gtgatgaggt gcatcaggaa ggagaatgtg gatgctgggg agagggccaa gcaggccctg

1081 gcctttgaga ggactgactt tgaccaggtg aggagcctga tggagaacag tgacaggtgc

1141 caggacatca ggaacctggc cttcctgggc attgcctaca acaccctgct gaggattgct

1201 gagattgcca ggatcagggt gaaggacatc agcaggactg atggtggcag gatgctgatc

1261 cacattggca ggaccaagac cctggtgagc actgctgggg tggagaaggc cctgtccctg

1321 ggtgtgacca agctggtgga gaggtggatc tctgtgtctg gtgtggcaga tgaccccaac

1381 aactacctgt tctgcagggt gaggaagaat ggggtggctg ccccctctgc caccagtcag

1441 ctgagcacca gggccctgga gggcatcttt gaggccaccc acaggctgat ctatggggcc

1501 aaggatgaca gtggccagag gtatctggcc tggtctggcc actctgccag ggtgggggct

1561 gccagggaca tggccagggc tggtgtgagc atccctgaga tcatgcaggc tggtggctgg

1621 accaatgtga acattgtgat gaactacatc aggaacctgg acagtgagac tggtgccatg

1681 gtgaggctgc tggaggatgg ggacgccggg ggaggtggca gtggtggagg cggatcaggt

1741 ggaggcggta gtgccgggac catgttgggt acccctgcag gaatgaacct tgaagctcga

1801 aaaacaaaga aaaaaatcaa agggattcag caagccactg caggagtctc acaagacact

1861 tcggaaaatc ctaacaaaac aatagttcct gcagcattac cacagctcac ccctaccttg

1921 gtgtcactgc tggaggtgat tgaacccgag gtgttgtatg caggatatga tagctctgtt

1981 ccagattcag catggagaat tatgaccaca ctcaacatgt taggtgggcg tcaagtgatt

2041 gcagcagtga aatgggcaaa ggcgatacca ggcttcagaa acttacacct ggatgaccaa

2101 atgaccctgc tacagtactc atggatgttt ctcatggcat ttgccctggg ttggagatca

2161 tacagacaat caagtggaaa cctgctctgc tttgctcctg atctgattat taatgagcag

2221 agaatgtctc taccctgcat gtatgaccaa tgtaaacaca tgctgtttgt ctcctctgaa

2281 ttacaaagat tgcaggtatc ctatgaagag tatctctgta tgaaaacctt actgcttctc

2341 tcctcagttc ctaaggaagg tctgaagagc caagagttat ttgatgagat tcgaatgact

2401 tatatcaaag agctaggaaa agccatcgtc aaaagggaag ggaactccag tcagaactgg

2461 caacggtttt accaactgac aaagcttctg gactccatgc atgaggtggt tgagaatctc

2521 cttacctact gcttccagac atttttggat aagaccatga gtattgaatt cccagaggcg

2581 gccgctgaaa tcatcactaa tcagatatat tcaaatggaa atatcaaaaa gcttctgttt

2641 catcagaagg ccggctaaac cggcaagctt cgatccagac atgataagat acattgatga

2701 gtttggacaa accacaacta gaatgcagtg aaaaaaatgc tttatttgtg aaatttgtga

2761 tgctattgct ttatttgtaa ccattataag ctgcaataaa caagttaaca acaacaattg

2821 cattcatttt atgtttcagg ttcaggggga ggtgtgggag gttttttcgg tatcagctca

2881 ctcaaaggcg gtaatacggt tatccacaga atcaggggat aacgcaggaa agaacatgtg

2941 agcaaaaggc cagcaaaagg ccaggaaccg taaaaaggcc gcgttgctgg cgtttttcca

3001 taggctccgc ccccctgacg agcatcacaa aaatcgacgc tcaagtcaga ggtggcgaaa

3061 cccgacagga ctataaagat accaggcgtt tccccctgga agctccctcg tgcgctctcc

3121 tgttccgacc ctgccgctta ccggatacct gtccgccttt ctcccttcgg gaagcgtggc

3181 gctttctcat agctcacgct gtaggtatct cagttcggtg taggtcgttc gctccaagct

3241 gggctgtgtg cacgaacccc ccgttcagcc cgaccgctgc gccttatccg gtaactatcg

3301 tcttgagtcc aacccggtaa gacacgactt atcgccactg gcagcagcca ctggtaacag

3361 gattagcaga gcgaggtatg taggcggtgc tacagagttc ttgaagtggt ggcctaacta

3421 cggctacact agaagaacag tatttggtat ctgcgctctg ctgaagccag ttaccttcgg

3481 aaaaagagtt ggtagctctt gatccggcaa acaaaccacc gctggtagcg gtggtttttt

3541 tgtttgcaag cagcagatta cgcgcagaaa aaaaggatct caagaagatc ctttgatctt

3601 ttctacgggg tctgacgctc agtggaacga aaactcacgt taagggattt tggtcatgag

3661 attatcaaaa aggatcttca cctagatcct tttaaattaa aaatgaagtt ttaaatcaat

3721 ctaaagtata tatgagtaaa cttggtctga cagcggccgc aaatgctaaa ccactgcagt

3781 ggttaccagt gcttgatcag tgaggcaccg atctcagcga tctgcctatt tcgttcgtcc

3841 atagtggcct gactccccgt cgtgtagatc actacgattc gtgagggctt accatcaggc

3901 cccagcgcag caatgatgcc gcgagagccg cgttcaccgg cccccgattt gtcagcaatg

3961 aaccagccag cagggagggc cgagcgaaga agtggtcctg ctactttgtc cgcctccatc

4021 cagtctatga gctgctgtcg tgatgctaga gtaagaagtt cgccagtgag tagtttccga

4081 agagttgtgg ccattgctac tggcatcgtg gtatcacgct cgtcgttcgg tatggcttcg

4141 ttcaactctg gttcccagcg gtcaagccgg gtcacatgat cacccatatt atgaagaaat

4201 gcagtcagct ccttagggcc tccgatcgtt gtcagaagta agttggccgc ggtgttgtcg

4261 ctcatggtaa tggcagcact acacaattct cttaccgtca tgccatccgt aagatgcttt

4321 tccgtgaccg gcgagtactc aaccaagtcg ttttgtgagt agtgtatacg gcgaccaagc

4381 tgctcttgcc cggcgtctat acgggacaac accgcgccac atagcagtac tttgaaagtg

4441 ctcatcatcg ggaatcgttc ttcggggcgg aaagactcaa ggatcttgcc gctattgaga

4501 tccagttcga tatagcccac tcttgcaccc agttgatctt cagcatcttt tactttcacc

4561 agcgtttcgg ggtgtgcaaa aacaggcaag caaaatgccg caaagaaggg aatgagtgcg

4621 acacgaaaat gttggatgct catactcgtc ctttttcaat attattgaag catttatcag

4681 ggttactagt acgtctctca aggataagta agtaatatta aggtacggga ggtattggac

4741 aggccgcaat aaaatatctt tattttcatt acatctgtgt gttggttttt tgtgtgaatc

4801 gatagtacta acatacgctc tccatcaaaa caaaacgaaa caaaacaaac tagcaaaata

4861 ggctgtcccc agtgcaagtg caggtgccag aacatttctc tggcctaact ggccggtacc

4921 gagctc

//

**0030.6M10t**

LOCUS 0030.6M10t 4875 bp DNA circular SYN 08-JUL-2021

DEFINITION synthetic circular DNA

ACCESSION .

VERSION .

KEYWORDS .

SOURCE synthetic DNA construct

ORGANISM synthetic DNA construct

REFERENCE 1 (bases 1 to 4875)

AUTHORS .

TITLE .

JOURNAL .

FEATURES Location/Qualifiers

source 1..4875

/organism="synthetic DNA construct"

/mol_type="other DNA"

promoter 16..63

/label=EM7 promoter

/note="synthetic bacterial promoter "

CDS 88..132

/codon_start=1

/label=3x GGGGS

/translation="GGGGSGGGGSGGGGS"

CDS 142..1123

/codon_start=1

/label=Cre R119C d(1-18)

/translation="MATSDEVRKNLMDMFRDRQAFSEHTWKMLLSVCRSWAAWCKLNNR KWFPAEPEDVRDYLLYLQARGLAVKTIQQHLGQLNMLHRRSGLPRPSDSNAVSLVMRCI RKENVDAGERAKQALAFERTDFDQVRSLMENSDRCQDIRNLAFLGIAYNTLLRIAEIAR IRVKDISRTDGGRMLIHIGRTKTLVSTAGVEKALSLGVTKLVERWISVSGVADDPNNYL FCRVRKNGVAAPSATSQLSTRALEGIFEATHRLIYGAKDDSGQRYLAWSGHSARVGAAR
DMARAGVSIPEIMQAGGWTNVNIVMNYIRNLDSETGAMVRLLEDGD"

CDS 1127..1171

/codon_start=1

/label=3x GGGGS

/translation="GEVAVVEADQVEAVV"

CDS 1195..2067

/codon_start=1

/label=GR LBD CS1/CD 504

/translation="AGMNLEARKTKKKIKGIQQATAGVSQDTSENPNKTIVPAALPQLT PTLVSLLEVIEPEVLYAGYDSSVPDSAWRIMTTLNMLGGRQVIAAVKWAKAIPGFRNLH LDDQMTLLQYSWMFLMAFALGWRSYRQSSGNLLCFAPDLIINEQRMSLPCMYDQCKHML FVSSELQRLQVSYEEYLCMKTLLLLSSVPKEGLKSQELFDEIRMTYIKELGKAIVKREG NSSQNWQRFYQLTKLLDSMHEVVENLLTYCFQTFLDKTMSIEFPEAAAEIITNQIYSNG
NIKKLLFHQK"

polyA_signal 2090..2224

/label=SV40 poly(A) signal

/note="SV40 polyadenylation signal"

rep_origin complement(2413..3001)

/direction=LEFT

/label=ori

/note="high-copy-number ColE1/pMB1/pBR322/pUC origin of

replication"

CDS complement(3201..4061)

/codon_start=1

/product="beta-lactamase"

/label=AmpR

/note="confers resistance to ampicillin, carbenicillin, and related antibiotics"

/translation="MSIQHFRVALIPFFAAFCLPVFAHPETLVKVKDAEDQLGARVGYI ELDLNSGKILESFRPEERFPMMSTFKVLLCGAVLSRIDAGQEQLGRRIHYSQNDLVEYS PVTEKHLTDGMTVRELCSAAITMSDNTAANLLLTTIGGPKELTAFLHNMGDHVTRLDRW EPELNEAIPNDERDTTMPVAMATTLRKLLTGELLTLASRQQLIDWMEADKVAGPLLRSA LPAGWFIADKSGAGERGSRGIIAALGPDGKPSRIVVIYTTGSQATMDERNRQIAEIGAS LIKHW"

polyA_signal 4166..4214

/note="synthetic polyadenylation signal"

misc_feature 4228..4319

/label=pause site

/note="RNA polymerase II transcriptional pause signal from the human alpha-2 globin gene"

enhancer 4345..4648

/label=CMV enhancer

/note="human cytomegalovirus immediate early enhancer"

promoter 4649..4852

/label=CMV promoter

/note="human cytomegalovirus (CMV) immediate early

promoter"

ORIGIN

1 ctagctcgag cacgtgttga caattaatca tcggcatagt atatcggcat agtataatac

61 gacaaggtga ggaactaaac catgtccgga ggcgggggct cgggaggtgg cggctctgga

121 ggaggcggta gtaccgggac catggccacc tctgatgagg tgaggaagaa cctgatggac

181 atgttcaggg acaggcaggc cttctctgag cacacctgga agatgctgct gtctgtgtgc

241 aggtcctggg ctgcctggtg caagctgaac aacaggaagt ggttccctgc tgagccagag

301 gatgtgaggg actacctgct gtacctgcag gccagaggcc tggctgtgaa gaccatccag

361 cagcacctgg gccagctgaa catgctccac aggagatctg gcctgcccag gccctctgac

421 agcaatgctg tgagcctggt gatgaggtgc atcaggaagg agaatgtgga tgctggggag

481 agggccaagc aggccctggc ctttgagagg actgactttg accaggtgag gagcctgatg

541 gagaacagtg acaggtgcca ggacatcagg aacctggcct tcctgggcat tgcctacaac

601 accctgctga ggattgctga gattgccagg atcagggtga aggacatcag caggactgat

661 ggtggcagga tgctgatcca cattggcagg accaagaccc tggtgagcac tgctggggtg

721 gagaaggccc tgtccctggg tgtgaccaag ctggtggaga ggtggatctc tgtgtctggt

781 gtggcagatg accccaacaa ctacctgttc tgcagggtga ggaagaatgg ggtggctgcc

841 ccctctgcca ccagtcagct gagcaccagg gccctggagg gcatctttga ggccacccac

901 aggctgatct atggggccaa ggatgacagt ggccagaggt atctggcctg gtctggccac

961 tctgccaggg tgggggctgc cagggacatg gccagggctg gtgtgagcat ccctgagatc

1021 atgcaggctg gtggctggac caatgtgaac attgtgatga actacatcag gaacctggac

1081 agtgagactg gtgccatggt gaggctgctg gaggatgggg acgccggggg aggtggcagt

1141 ggtggaggcg gatcaggtgg aggcggtagt gccgggacca tgttgggtac ccctgcagga

1201 atgaaccttg aagctcgaaa aacaaagaaa aaaatcaaag ggattcagca agccactgca

1261 ggagtctcac aagacacttc ggaaaatcct aacaaaacaa tagttcctgc agcattacca

1321 cagctcaccc ctaccttggt gtcactgctg gaggtgattg aacccgaggt gttgtatgca

1381 ggatatgata gctctgttcc agattcagca tggagaatta tgaccacact caacatgtta

1441 ggtgggcgtc aagtgattgc agcagtgaaa tgggcaaagg cgataccagg cttcagaaac

1501 ttacacctgg atgaccaaat gaccctgcta cagtactcat ggatgtttct catggcattt

1561 gccctgggtt ggagatcata cagacaatca agtggaaacc tgctctgctt tgctcctgat

1621 ctgattatta atgagcagag aatgtctcta ccctgcatgt atgaccaatg taaacacatg

1681 ctgtttgtct cctctgaatt acaaagattg caggtatcct atgaagagta tctctgtatg

1741 aaaaccttac tgcttctctc ctcagttcct aaggaaggtc tgaagagcca agagttattt

1801 gatgagattc gaatgactta tatcaaagag ctaggaaaag ccatcgtcaa aagggaaggg

1861 aactccagtc agaactggca acggttttac caactgacaa agcttctgga ctccatgcat

1921 gaggtggttg agaatctcct tacctactgc ttccagacat ttttggataa gaccatgagt

1981 attgaattcc cagaggcggc cgctgaaatc atcactaatc agatatattc aaatggaaat

2041 atcaaaaagc ttctgtttca tcagaaggcc ggctaaaccg gcaagcttcg atccagacat

2101 gataagatac attgatgagt ttggacaaac cacaactaga atgcagtgaa aaaaatgctt

2161 tatttgtgaa atttgtgatg ctattgcttt atttgtaacc attataagct gcaataaaca

2221 agttaacaac aacaattgca ttcattttat gtttcaggtt cagggggagg tgtgggaggt

2281 tttttcggta tcagctcact caaaggcggt aatacggtta tccacagaat caggggataa

2341 cgcaggaaag aacatgtgag caaaaggcca gcaaaaggcc aggaaccgta aaaaggccgc

2401 gttgctggcg tttttccata ggctccgccc ccctgacgag catcacaaaa atcgacgctc

2461 aagtcagagg tggcgaaacc cgacaggact ataaagatac caggcgtttc cccctggaag

2521 ctccctcgtg cgctctcctg ttccgaccct gccgcttacc ggatacctgt ccgcctttct

2581 cccttcggga agcgtggcgc tttctcatag ctcacgctgt aggtatctca gttcggtgta

2641 ggtcgttcgc tccaagctgg gctgtgtgca cgaacccccc gttcagcccg accgctgcgc

2701 cttatccggt aactatcgtc ttgagtccaa cccggtaaga cacgacttat cgccactggc

2761 agcagccact ggtaacagga ttagcagagc gaggtatgta ggcggtgcta cagagttctt

2821 gaagtggtgg cctaactacg gctacactag aagaacagta tttggtatct gcgctctgct

2881 gaagccagtt accttcggaa aaagagttgg tagctcttga tccggcaaac aaaccaccgc

2941 tggtagcggt ggtttttttg tttgcaagca gcagattacg cgcagaaaaa aaggatctca

3001 agaagatcct ttgatctttt ctacggggtc tgacgctcag tggaacgaaa actcacgtta

3061 agggattttg gtcatgagat tatcaaaaag gatcttcacc tagatccttt taaattaaaa

3121 atgaagtttt aaatcaatct aaagtatata tgagtaaact tggtctgaca gcggccgcaa

3181 atgctaaacc actgcagtgg ttaccagtgc ttgatcagtg aggcaccgat ctcagcgatc

3241 tgcctatttc gttcgtccat agtggcctga ctccccgtcg tgtagatcac tacgattcgt

3301 gagggcttac catcaggccc cagcgcagca atgatgccgc gagagccgcg ttcaccggcc

3361 cccgatttgt cagcaatgaa ccagccagca gggagggccg agcgaagaag tggtcctgct

3421 actttgtccg cctccatcca gtctatgagc tgctgtcgtg atgctagagt aagaagttcg

3481 ccagtgagta gtttccgaag agttgtggcc attgctactg gcatcgtggt atcacgctcg

3541 tcgttcggta tggcttcgtt caactctggt tcccagcggt caagccgggt cacatgatca

3601 cccatattat gaagaaatgc agtcagctcc ttagggcctc cgatcgttgt cagaagtaag

3661 ttggccgcgg tgttgtcgct catggtaatg gcagcactac acaattctct taccgtcatg

3721 ccatccgtaa gatgcttttc cgtgaccggc gagtactcaa ccaagtcgtt ttgtgagtag

3781 tgtatacggc gaccaagctg ctcttgcccg gcgtctatac gggacaacac cgcgccacat

3841 agcagtactt tgaaagtgct catcatcggg aatcgttctt cggggcggaa agactcaagg

3901 atcttgccgc tattgagatc cagttcgata tagcccactc ttgcacccag ttgatcttca

3961 gcatctttta ctttcaccag cgtttcgggg tgtgcaaaaa caggcaagca aaatgccgca

4021 aagaagggaa tgagtgcgac acgaaaatgt tggatgctca tactcgtcct ttttcaatat

4081 tattgaagca tttatcaggg ttactagtac gtctctcaag gataagtaag taatattaag

4141 gtacgggagg tattggacag gccgcaataa aatatcttta ttttcattac atctgtgtgt

4201 tggttttttg tgtgaatcga tagtactaac atacgctctc catcaaaaca aaacgaaaca

4261 aaacaaacta gcaaaatagg ctgtccccag tgcaagtgca ggtgccagaa catttctctg

4321 gcctaactgg ccggtaccga gctccgttac ataacttacg gtaaatggcc cgcctggctg

4381 accgcccaac gacccccgcc cattgacgtc aataatgacg tatgttccca tagtaacgcc

4441 aatagggact ttccattgac gtcaatgggt ggagtattta cggtaaactg cccacttggc

4501 agtacatcaa gtgtatcata tgccaagtac gccccctatt gacgtcaatg acggtaaatg

4561 gcccgcctgg cattatgccc agtacatgac cttatgggac tttcctactt ggcagtacat

4621 ctacgtatta gtcatcgcta ttaccatggt gatgcggttt tggcagtaca tcaatgggcg

4681 tggatagcgg tttgactcac ggggatttcc aagtctccac cccattgacg tcaatgggag

4741 tttgttttgg caccaaaatc aacgggactt tccaaaatgt cgtaacaact ccgccccatt

4801 gacgcaaatg ggcggtaggc gtgtacggtg ggaggtctat ataagcagag ctggtttagt

4861 gaaccgtcag atccg

//

**0030.6M11:**

LOCUS 0030.6M11 4926 bp DNA circular SYN 08-JUL-2021

DEFINITION synthetic circular DNA

ACCESSION .

VERSION .

KEYWORDS .

SOURCE synthetic DNA construct

ORGANISM synthetic DNA construct

REFERENCE 1 (bases 1 to 4926)

AUTHORS .

TITLE .

JOURNAL .

FEATURES Location/Qualifiers

source 1..4926

/organism="synthetic DNA construct"

/mol_type="other DNA"

enhancer 1..304

/label=CMV enhancer

/note="human cytomegalovirus immediate early enhancer"

promoter 305..508

/label=CMV promoter

/note="human cytomegalovirus (CMV) immediate early

promoter"

promoter 547..594

/label=EM7 promoter

/note="synthetic bacterial promoter "

CDS 619..663

/codon_start=1

/label=3x GGGGS

/translation="GGGGSGGGGSGGGGS"

CDS 673..1704

/codon_start=1

/label=huCre R119V

/translation="MVSNLLTVHQNLPALPVDATSDEVRKNLMDMFRDRQAFSEHTWKM LLSVCRSWAAWCKLNNRKWFPAEPEDVRDYLLYLQARGLAVKTIQQHLGQLNMLHRRSG LPRPSDSNAVSLVMRVIRKENVDAGERAKQALAFERTDFDQVRSLMENSDRCQDIRNLA FLGIAYNTLLRIAEIARIRVKDISRTDGGRMLIHIGRTKTLVSTAGVEKALSLGVTKLV ERWISVSGVADDPNNYLFCRVRKNGVAAPSATSQLSTRALEGIFEATHRLIYGAKDDSG QRYLAWSGHSARVGAARDMARAGVSIPEIMQAGGWTNVNIVMNYIRNLDSETGAMVRLL EDGD"

CDS 1709..1753

/codon_start=1

/label=3x GGGGS

/translation="GEVAVVEADQVEAVV"

CDS 1777..2649

/codon_start=1

/label=GR LBD CS1/CD 504

/translation="AGMNLEARKTKKKIKGIQQATAGVSQDTSENPNKTIVPAALPQLT PTLVSLLEVIEPEVLYAGYDSSVPDSAWRIMTTLNMLGGRQVIAAVKWAKAIPGFRNLH LDDQMTLLQYSWMFLMAFALGWRSYRQSSGNLLCFAPDLIINEQRMSLPCMYDQCKHML FVSSELQRLQVSYEEYLCMKTLLLLSSVPKEGLKSQELFDEIRMTYIKELGKAIVKREG NSSQNWQRFYQLTKLLDSMHEVVENLLTYCFQTFLDKTMSIEFPEAAAEIITNQIYSNG NIKKLLFHQK"

polyA_signal 2672..2806

/label=SV40 poly(A) signal

/note="SV40 polyadenylation signal"

rep_origin complement(2995..3583)

/direction=LEFT

/label=ori

/note="high-copy-number ColE1/pMB1/pBR322/pUC origin of

replication"

CDS complement(3783..4643)

/codon_start=1

/product="beta-lactamase"

/label=AmpR

/note="confers resistance to ampicillin, carbenicillin, and related antibiotics"

/translation="MSIQHFRVALIPFFAAFCLPVFAHPETLVKVKDAEDQLGARVGYI ELDLNSGKILESFRPEERFPMMSTFKVLLCGAVLSRIDAGQEQLGRRIHYSQNDLVEYS PVTEKHLTDGMTVRELCSAAITMSDNTAANLLLTTIGGPKELTAFLHNMGDHVTRLDRW EPELNEAIPNDERDTTMPVAMATTLRKLLTGELLTLASRQQLIDWMEADKVAGPLLRSA LPAGWFIADKSGAGERGSRGIIAALGPDGKPSRIVVIYTTGSQATMDERNRQIAEIGAS LIKHW"

polyA_signal 4748..4796

/note="synthetic polyadenylation signal"

misc_feature 4810..4901

/label=pause site

/note="RNA polymerase II transcriptional pause signal from the human alpha-2 globin gene"

ORIGIN

1 cgttacataa cttacggtaa atggcccgcc tggctgaccg cccaacgacc cccgcccatt

61 gacgtcaata atgacgtatg ttcccatagt aacgccaata gggactttcc attgacgtca

121 atgggtggag tatttacggt aaactgccca cttggcagta catcaagtgt atcatatgcc

181 aagtacgccc cctattgacg tcaatgacgg taaatggccc gcctggcatt atgcccagta

241 catgacctta tgggactttc ctacttggca gtacatctac gtattagtca tcgctattac

301 catggtgatg cggttttggc agtacatcaa tgggcgtgga tagcggtttg actcacgggg

361 atttccaagt ctccacccca ttgacgtcaa tgggagtttg ttttggcacc aaaatcaacg

421 ggactttcca aaatgtcgta acaactccgc cccattgacg caaatgggcg gtaggcgtgt

481 acggtgggag gtctatataa gcagagctgg tttagtgaac cgtcagatcc gctagctcga

541 gcacgtgttg acaattaatc atcggcatag tatatcggca tagtataata cgacaaggtg

601 aggaactaaa ccatgtccgg aggcgggggc tcgggaggtg gcggctctgg aggaggcggt

661 agtaccggga ccatggtgag caacctgctg actgtgcacc agaacctgcc tgccctgcct

721 gtggatgcca cctctgatga ggtgaggaag aacctgatgg acatgttcag ggacaggcag

781 gccttctctg agcacacctg gaagatgctg ctgtctgtgt gcaggtcctg ggctgcctgg

841 tgcaagctga acaacaggaa gtggttccct gctgagccag aggatgtgag ggactacctg

901 ctgtacctgc aggccagagg cctggctgtg aagaccatcc agcagcacct gggccagctg

961 aacatgctcc acaggagatc tggcctgccc aggccctctg acagcaatgc tgtgagcctg

1021 gtgatgaggg taatcaggaa ggagaatgtg gatgctgggg agagggccaa gcaggccctg

1081 gcctttgaga ggactgactt tgaccaggtg aggagcctga tggagaacag tgacaggtgc

1141 caggacatca ggaacctggc cttcctgggc attgcctaca acaccctgct gaggattgct

1201 gagattgcca ggatcagggt gaaggacatc agcaggactg atggtggcag gatgctgatc

1261 cacattggca ggaccaagac cctggtgagc actgctgggg tggagaaggc cctgtccctg

1321 ggtgtgacca agctggtgga gaggtggatc tctgtgtctg gtgtggcaga tgaccccaac

1381 aactacctgt tctgcagggt gaggaagaat ggggtggctg ccccctctgc caccagtcag

1441 ctgagcacca gggccctgga gggcatcttt gaggccaccc acaggctgat ctatggggcc

1501 aaggatgaca gtggccagag gtatctggcc tggtctggcc actctgccag ggtgggggct

1561 gccagggaca tggccagggc tggtgtgagc atccctgaga tcatgcaggc tggtggctgg

1621 accaatgtga acattgtgat gaactacatc aggaacctgg acagtgagac tggtgccatg

1681 gtgaggctgc tggaggatgg ggacgccggg ggaggtggca gtggtggagg cggatcaggt

1741 ggaggcggta gtgccgggac catgttgggt acccctgcag gaatgaacct tgaagctcga

1801 aaaacaaaga aaaaaatcaa agggattcag caagccactg caggagtctc acaagacact

1861 tcggaaaatc ctaacaaaac aatagttcct gcagcattac cacagctcac ccctaccttg

1921 gtgtcactgc tggaggtgat tgaacccgag gtgttgtatg caggatatga tagctctgtt

1981 ccagattcag catggagaat tatgaccaca ctcaacatgt taggtgggcg tcaagtgatt

2041 gcagcagtga aatgggcaaa ggcgatacca ggcttcagaa acttacacct ggatgaccaa

2101 atgaccctgc tacagtactc atggatgttt ctcatggcat ttgccctggg ttggagatca

2161 tacagacaat caagtggaaa cctgctctgc tttgctcctg atctgattat taatgagcag

2221 agaatgtctc taccctgcat gtatgaccaa tgtaaacaca tgctgtttgt ctcctctgaa

2281 ttacaaagat tgcaggtatc ctatgaagag tatctctgta tgaaaacctt actgcttctc

2341 tcctcagttc ctaaggaagg tctgaagagc caagagttat ttgatgagat tcgaatgact

2401 tatatcaaag agctaggaaa agccatcgtc aaaagggaag ggaactccag tcagaactgg

2461 caacggtttt accaactgac aaagcttctg gactccatgc atgaggtggt tgagaatctc

2521 cttacctact gcttccagac atttttggat aagaccatga gtattgaatt cccagaggcg

2581 gccgctgaaa tcatcactaa tcagatatat tcaaatggaa atatcaaaaa gcttctgttt

2641 catcagaagg ccggctaaac cggcaagctt cgatccagac atgataagat acattgatga

2701 gtttggacaa accacaacta gaatgcagtg aaaaaaatgc tttatttgtg aaatttgtga

2761 tgctattgct ttatttgtaa ccattataag ctgcaataaa caagttaaca acaacaattg

2821 cattcatttt atgtttcagg ttcaggggga ggtgtgggag gttttttcgg tatcagctca

2881 ctcaaaggcg gtaatacggt tatccacaga atcaggggat aacgcaggaa agaacatgtg

2941 agcaaaaggc cagcaaaagg ccaggaaccg taaaaaggcc gcgttgctgg cgtttttcca

3001 taggctccgc ccccctgacg agcatcacaa aaatcgacgc tcaagtcaga ggtggcgaaa

3061 cccgacagga ctataaagat accaggcgtt tccccctgga agctccctcg tgcgctctcc

3121 tgttccgacc ctgccgctta ccggatacct gtccgccttt ctcccttcgg gaagcgtggc

3181 gctttctcat agctcacgct gtaggtatct cagttcggtg taggtcgttc gctccaagct

3241 gggctgtgtg cacgaacccc ccgttcagcc cgaccgctgc gccttatccg gtaactatcg

3301 tcttgagtcc aacccggtaa gacacgactt atcgccactg gcagcagcca ctggtaacag

3361 gattagcaga gcgaggtatg taggcggtgc tacagagttc ttgaagtggt ggcctaacta

3421 cggctacact agaagaacag tatttggtat ctgcgctctg ctgaagccag ttaccttcgg

3481 aaaaagagtt ggtagctctt gatccggcaa acaaaccacc gctggtagcg gtggtttttt

3541 tgtttgcaag cagcagatta cgcgcagaaa aaaaggatct caagaagatc ctttgatctt

3601 ttctacgggg tctgacgctc agtggaacga aaactcacgt taagggattt tggtcatgag

3661 attatcaaaa aggatcttca cctagatcct tttaaattaa aaatgaagtt ttaaatcaat

3721 ctaaagtata tatgagtaaa cttggtctga cagcggccgc aaatgctaaa ccactgcagt

3781 ggttaccagt gcttgatcag tgaggcaccg atctcagcga tctgcctatt tcgttcgtcc

3841 atagtggcct gactccccgt cgtgtagatc actacgattc gtgagggctt accatcaggc

3901 cccagcgcag caatgatgcc gcgagagccg cgttcaccgg cccccgattt gtcagcaatg

3961 aaccagccag cagggagggc cgagcgaaga agtggtcctg ctactttgtc cgcctccatc

4021 cagtctatga gctgctgtcg tgatgctaga gtaagaagtt cgccagtgag tagtttccga

4081 agagttgtgg ccattgctac tggcatcgtg gtatcacgct cgtcgttcgg tatggcttcg

4141 ttcaactctg gttcccagcg gtcaagccgg gtcacatgat cacccatatt atgaagaaat

4201 gcagtcagct ccttagggcc tccgatcgtt gtcagaagta agttggccgc ggtgttgtcg

4261 ctcatggtaa tggcagcact acacaattct cttaccgtca tgccatccgt aagatgcttt

4321 tccgtgaccg gcgagtactc aaccaagtcg ttttgtgagt agtgtatacg gcgaccaagc

4381 tgctcttgcc cggcgtctat acgggacaac accgcgccac atagcagtac tttgaaagtg

4441 ctcatcatcg ggaatcgttc ttcggggcgg aaagactcaa ggatcttgcc gctattgaga

4501 tccagttcga tatagcccac tcttgcaccc agttgatctt cagcatcttt tactttcacc

4561 agcgtttcgg ggtgtgcaaa aacaggcaag caaaatgccg caaagaaggg aatgagtgcg

4621 acacgaaaat gttggatgct catactcgtc ctttttcaat attattgaag catttatcag

4681 ggttactagt acgtctctca aggataagta agtaatatta aggtacggga ggtattggac

4741 aggccgcaat aaaatatctt tattttcatt acatctgtgt gttggttttt tgtgtgaatc

4801 gatagtacta acatacgctc tccatcaaaa caaaacgaaa caaaacaaac tagcaaaata

4861 ggctgtcccc agtgcaagtg caggtgccag aacatttctc tggcctaact ggccggtacc

4921 gagctc

//

**0030.6M11t:**

LOCUS 0030.6M11t 4875 bp DNA circular SYN 08-JUL-2021

DEFINITION synthetic circular DNA

ACCESSION .

VERSION .

KEYWORDS .

SOURCE synthetic DNA construct

ORGANISM synthetic DNA construct

REFERENCE 1 (bases 1 to 4875)

AUTHORS .

TITLE .

JOURNAL .

FEATURES Location/Qualifiers

source 1..4875

/organism="synthetic DNA construct"

/mol_type="other DNA"

promoter 16..63

/label=EM7 promoter

/note="synthetic bacterial promoter "

CDS 88..132

/codon_start=1

/label=3x GGGGS

/translation="GGGGSGGGGSGGGGS"

CDS 142..1122

/codon_start=1

/label=Cre R119V d(1-18)

/translation="MATSDEVRKNLMDMFRDRQAFSEHTWKMLLSVCRSWAAWCKLNNR KWFPAEPEDVRDYLLYLQARGLAVKTIQQHLGQLNMLHRRSGLPRPSDSNAVSLVMRVI RKENVDAGERAKQALAFERTDFDQVRSLMENSDRCQDIRNLAFLGIAYNTLLRIAEIAR IRVKDISRTDGGRMLIHIGRTKTLVSTAGVEKALSLGVTKLVERWISVSGVADDPNNYL FCRVRKNGVAAPSATSQLSTRALEGIFEATHRLIYGAKDDSGQRYLAWSGHSARVGAAR DMARAGVSIPEIMQAGGWTNVNIVMNYIRNLDSETGAMVRLLEDGD"

CDS 1127..1171

/codon_start=1

/label=3x GGGGS

/translation="GEVAVVEADQVEAVV"

CDS 1195..2067

/codon_start=1

/label=GR LBD CS1/CD 504

/translation="AGMNLEARKTKKKIKGIQQATAGVSQDTSENPNKTIVPAALPQLT PTLVSLLEVIEPEVLYAGYDSSVPDSAWRIMTTLNMLGGRQVIAAVKWAKAIPGFRNLH LDDQMTLLQYSWMFLMAFALGWRSYRQSSGNLLCFAPDLIINEQRMSLPCMYDQCKHML FVSSELQRLQVSYEEYLCMKTLLLLSSVPKEGLKSQELFDEIRMTYIKELGKAIVKREG NSSQNWQRFYQLTKLLDSMHEVVENLLTYCFQTFLDKTMSIEFPEAAAEIITNQIYSNG NIKKLLFHQK"

polyA_signal 2090..2224

/label=SV40 poly(A) signal

/note="SV40 polyadenylation signal"

rep_origin complement(2413..3001)

/direction=LEFT

/label=ori

/note="high-copy-number ColE1/pMB1/pBR322/pUC origin of

replication"

CDS complement(3201..4061)

/codon_start=1

/product="beta-lactamase"

/label=AmpR

/note="confers resistance to ampicillin, carbenicillin, and related antibiotics"

/translation="MSIQHFRVALIPFFAAFCLPVFAHPETLVKVKDAEDQLGARVGYI ELDLNSGKILESFRPEERFPMMSTFKVLLCGAVLSRIDAGQEQLGRRIHYSQNDLVEYS PVTEKHLTDGMTVRELCSAAITMSDNTAANLLLTTIGGPKELTAFLHNMGDHVTRLDRW EPELNEAIPNDERDTTMPVAMATTLRKLLTGELLTLASRQQLIDWMEADKVAGPLLRSA LPAGWFIADKSGAGERGSRGIIAALGPDGKPSRIVVIYTTGSQATMDERNRQIAEIGAS LIKHW"

polyA_signal 4166..4214

/note="synthetic polyadenylation signal"

misc_feature 4228..4319

/label=pause site

/note="RNA polymerase II transcriptional pause signal from the human alpha-2 globin gene"

enhancer 4345..4648

/label=CMV enhancer

/note="human cytomegalovirus immediate early enhancer"

promoter 4649..4852

/label=CMV promoter

/note="human cytomegalovirus (CMV) immediate early

promoter"

ORIGIN

1 ctagctcgag cacgtgttga caattaatca tcggcatagt atatcggcat agtataatac

61 gacaaggtga ggaactaaac catgtccgga ggcgggggct cgggaggtgg cggctctgga

121 ggaggcggta gtaccgggac catggccacc tctgatgagg tgaggaagaa cctgatggac

181 atgttcaggg acaggcaggc cttctctgag cacacctgga agatgctgct gtctgtgtgc

241 aggtcctggg ctgcctggtg caagctgaac aacaggaagt ggttccctgc tgagccagag

301 gatgtgaggg actacctgct gtacctgcag gccagaggcc tggctgtgaa gaccatccag

361 cagcacctgg gccagctgaa catgctccac aggagatctg gcctgcccag gccctctgac

421 agcaatgctg tgagcctggt gatgagggta atcaggaagg agaatgtgga tgctggggag

481 agggccaagc aggccctggc ctttgagagg actgactttg accaggtgag gagcctgatg

541 gagaacagtg acaggtgcca ggacatcagg aacctggcct tcctgggcat tgcctacaac

601 accctgctga ggattgctga gattgccagg atcagggtga aggacatcag caggactgat

661 ggtggcagga tgctgatcca cattggcagg accaagaccc tggtgagcac tgctggggtg

721 gagaaggccc tgtccctggg tgtgaccaag ctggtggaga ggtggatctc tgtgtctggt

781 gtggcagatg accccaacaa ctacctgttc tgcagggtga ggaagaatgg ggtggctgcc

841 ccctctgcca ccagtcagct gagcaccagg gccctggagg gcatctttga ggccacccac

901 aggctgatct atggggccaa ggatgacagt ggccagaggt atctggcctg gtctggccac

961 tctgccaggg tgggggctgc cagggacatg gccagggctg gtgtgagcat ccctgagatc

1021 atgcaggctg gtggctggac caatgtgaac attgtgatga actacatcag gaacctggac

1081 agtgagactg gtgccatggt gaggctgctg gaggatgggg acgccggggg aggtggcagt

1141 ggtggaggcg gatcaggtgg aggcggtagt gccgggacca tgttgggtac ccctgcagga

1201 atgaaccttg aagctcgaaa aacaaagaaa aaaatcaaag ggattcagca agccactgca

1261 ggagtctcac aagacacttc ggaaaatcct aacaaaacaa tagttcctgc agcattacca

1321 cagctcaccc ctaccttggt gtcactgctg gaggtgattg aacccgaggt gttgtatgca

1381 ggatatgata gctctgttcc agattcagca tggagaatta tgaccacact caacatgtta

1441 ggtgggcgtc aagtgattgc agcagtgaaa tgggcaaagg cgataccagg cttcagaaac

1501 ttacacctgg atgaccaaat gaccctgcta cagtactcat ggatgtttct catggcattt

1561 gccctgggtt ggagatcata cagacaatca agtggaaacc tgctctgctt tgctcctgat

1621 ctgattatta atgagcagag aatgtctcta ccctgcatgt atgaccaatg taaacacatg

1681 ctgtttgtct cctctgaatt acaaagattg caggtatcct atgaagagta tctctgtatg

1741 aaaaccttac tgcttctctc ctcagttcct aaggaaggtc tgaagagcca agagttattt

1801 gatgagattc gaatgactta tatcaaagag ctaggaaaag ccatcgtcaa aagggaaggg

1861 aactccagtc agaactggca acggttttac caactgacaa agcttctgga ctccatgcat

1921 gaggtggttg agaatctcct tacctactgc ttccagacat ttttggataa gaccatgagt

1981 attgaattcc cagaggcggc cgctgaaatc atcactaatc agatatattc aaatggaaat

2041 atcaaaaagc ttctgtttca tcagaaggcc ggctaaaccg gcaagcttcg atccagacat

2101 gataagatac attgatgagt ttggacaaac cacaactaga atgcagtgaa aaaaatgctt

2161 tatttgtgaa atttgtgatg ctattgcttt atttgtaacc attataagct gcaataaaca

2221 agttaacaac aacaattgca ttcattttat gtttcaggtt cagggggagg tgtgggaggt

2281 tttttcggta tcagctcact caaaggcggt aatacggtta tccacagaat caggggataa

2341 cgcaggaaag aacatgtgag caaaaggcca gcaaaaggcc aggaaccgta aaaaggccgc

2401 gttgctggcg tttttccata ggctccgccc ccctgacgag catcacaaaa atcgacgctc

2461 aagtcagagg tggcgaaacc cgacaggact ataaagatac caggcgtttc cccctggaag

2521 ctccctcgtg cgctctcctg ttccgaccct gccgcttacc ggatacctgt ccgcctttct

2581 cccttcggga agcgtggcgc tttctcatag ctcacgctgt aggtatctca gttcggtgta

2641 ggtcgttcgc tccaagctgg gctgtgtgca cgaacccccc gttcagcccg accgctgcgc

2701 cttatccggt aactatcgtc ttgagtccaa cccggtaaga cacgacttat cgccactggc

2761 agcagccact ggtaacagga ttagcagagc gaggtatgta ggcggtgcta cagagttctt

2821 gaagtggtgg cctaactacg gctacactag aagaacagta tttggtatct gcgctctgct

2881 gaagccagtt accttcggaa aaagagttgg tagctcttga tccggcaaac aaaccaccgc

2941 tggtagcggt ggtttttttg tttgcaagca gcagattacg cgcagaaaaa aaggatctca

3001 agaagatcct ttgatctttt ctacggggtc tgacgctcag tggaacgaaa actcacgtta

3061 agggattttg gtcatgagat tatcaaaaag gatcttcacc tagatccttt taaattaaaa

3121 atgaagtttt aaatcaatct aaagtatata tgagtaaact tggtctgaca gcggccgcaa

3181 atgctaaacc actgcagtgg ttaccagtgc ttgatcagtg aggcaccgat ctcagcgatc

3241 tgcctatttc gttcgtccat agtggcctga ctccccgtcg tgtagatcac tacgattcgt

3301 gagggcttac catcaggccc cagcgcagca atgatgccgc gagagccgcg ttcaccggcc

3361 cccgatttgt cagcaatgaa ccagccagca gggagggccg agcgaagaag tggtcctgct

3421 actttgtccg cctccatcca gtctatgagc tgctgtcgtg atgctagagt aagaagttcg

3481 ccagtgagta gtttccgaag agttgtggcc attgctactg gcatcgtggt atcacgctcg

3541 tcgttcggta tggcttcgtt caactctggt tcccagcggt caagccgggt cacatgatca

3601 cccatattat gaagaaatgc agtcagctcc ttagggcctc cgatcgttgt cagaagtaag

3661 ttggccgcgg tgttgtcgct catggtaatg gcagcactac acaattctct taccgtcatg

3721 ccatccgtaa gatgcttttc cgtgaccggc gagtactcaa ccaagtcgtt ttgtgagtag

3781 tgtatacggc gaccaagctg ctcttgcccg gcgtctatac gggacaacac cgcgccacat

3841 agcagtactt tgaaagtgct catcatcggg aatcgttctt cggggcggaa agactcaagg

3901 atcttgccgc tattgagatc cagttcgata tagcccactc ttgcacccag ttgatcttca

3961 gcatctttta ctttcaccag cgtttcgggg tgtgcaaaaa caggcaagca aaatgccgca

4021 aagaagggaa tgagtgcgac acgaaaatgt tggatgctca tactcgtcct ttttcaatat

4081 tattgaagca tttatcaggg ttactagtac gtctctcaag gataagtaag taatattaag

4141 gtacgggagg tattggacag gccgcaataa aatatcttta ttttcattac atctgtgtgt

4201 tggttttttg tgtgaatcga tagtactaac atacgctctc catcaaaaca aaacgaaaca

4261 aaacaaacta gcaaaatagg ctgtccccag tgcaagtgca ggtgccagaa catttctctg

4321 gcctaactgg ccggtaccga gctccgttac ataacttacg gtaaatggcc cgcctggctg

4381 accgcccaac gacccccgcc cattgacgtc aataatgacg tatgttccca tagtaacgcc

4441 aatagggact ttccattgac gtcaatgggt ggagtattta cggtaaactg cccacttggc

4501 agtacatcaa gtgtatcata tgccaagtac gccccctatt gacgtcaatg acggtaaatg

4561 gcccgcctgg cattatgccc agtacatgac cttatgggac tttcctactt ggcagtacat

4621 ctacgtatta gtcatcgcta ttaccatggt gatgcggttt tggcagtaca tcaatgggcg

4681 tggatagcgg tttgactcac ggggatttcc aagtctccac cccattgacg tcaatgggag

4741 tttgttttgg caccaaaatc aacgggactt tccaaaatgt cgtaacaact ccgccccatt

4801 gacgcaaatg ggcggtaggc gtgtacggtg ggaggtctat ataagcagag ctggtttagt

4861 gaaccgtcag atccg

//

**CA97.2:**

LOCUS CA97.2 3917 bp DNA circular SYN 06-JUL-2021

DEFINITION synthetic circular DNA

ACCESSION .

VERSION .

KEYWORDS .

SOURCE synthetic DNA construct

ORGANISM synthetic DNA construct

REFERENCE 1 (bases 1 to 3917)

AUTHORS .

TITLE .

JOURNAL .

FEATURES Location/Qualifiers

source 1..3917

/organism="synthetic DNA construct"

/mol_type="other DNA"

promoter 19..348

/label=SV40 promoter

/note="SV40 enhancer and early promoter"

rep_origin 199..334

/label=SV40 ori

/note="SV40 origin of replication"

promoter 441..710

/label=TK promoter

enhancer 717..1020

/label=CMV enhancer

/note="human cytomegalovirus immediate early enhancer"

promoter 1021..1224

/label=CMV promoter

/note="human cytomegalovirus (CMV) immediate early

promoter"

promoter 1252..1270

/label=T7 promoter

/note="promoter for bacteriophage T7 RNA polymerase"

CDS 1296..1340

/codon_start=1

/label=3xGGGS

/translation="GGGGSGGGGSGGGGS"

CDS 1347..1490

/codon_start=1

/label=8xEAAAK

/translation="AEAAAKEAAAKEAAAKEAAAKALEAEAAAKEAAAKEAAAKEAAAK
APG"

CDS 1489..1533

/codon_start=1

/label=3xGGGS

/translation="GEVAVVEADQVEAVV"

polyA_signal 1546..1680

/label=SV40 poly(A) signal

/note="SV40 polyadenylation signal"

rep_origin complement(2045..2633)

/direction=LEFT

/label=ori

/note="high-copy-number ColE1/pMB1/pBR322/pUC origin of

replication"

CDS complement(2804..3664)

/codon_start=1

/gene="bla"

/product="beta-lactamase"

/label=AmpR

/note="confers resistance to ampicillin, carbenicillin, and related antibiotics"

/translation="MSIQHFRVALIPFFAAFCLPVFAHPETLVKVKDAEDQLGARVGYI ELDLNSGKILESFRPEERFPMMSTFKVLLCGAVLSRVDAGQEQLGRRIHYSQNDLVEYS PVTEKHLTDGMTVRELCSAAITMSDNTAANLLLTTIGGPKELTAFLHNMGDHVTRLDRW EPELNEAIPNDERDTTMPVAMATTLRKLLTGELLTLASRQQLIDWMEADKVAGPLLRSA LPAGWFIADKSGAGERGSRGIIAALGPDGKPSRIVVIYTTGSQATMDERNRQIAEIGAS LIKHW"

promoter complement(3665..3769)

/gene="bla"

/label=AmpR promoter

ORIGIN

1 agctagcttc tgtggaatgt gtgtcagtta gggtgtggaa agtccccagg ctccccagca

61 ggcagaagta tgcaaagcat gcatctcaat tagtcagcaa ccaggtgtgg aaagtcccca

121 ggctccccag caggcagaag tatgcaaagc atgcatctca attagtcagc aaccatagtc

181 ccgcccctaa ctccgcccat cccgccccta actccgccca gttccgccca ttctccgccc

241 catggctgac taattttttt tatttatgca gaggccgagg ccgcctcggc ctctgagcta

301 ttccagaagt agtgaggagg cttttttgga ggcctaggct tttgcaaaaa gctccctcga

361 ggaactggaa aaccagaaag ttaactggta agtttagtct ttttgtcttt tatttcaggt

421 cccggatcga attgcggccg ctcgagcagt gtggttttca agaggaagca aaaagcctct

481 ccacccaggc ctggaatgtt tccacccaat gtcgagcagt gtggttttgc aagaggaagc

541 aaaaagcctc tccacccagg cctggaatgt ttccacccaa tgtcgagcaa accccgccca

601 gcgtcttgtc attggcgaat tcgaacacgc agatgcagtc ggggcggcgc ggtcccaggt

661 ccacttcgca tattaaggtg acgcgtgtgg cctcgaacac cgagcgaccc tctagacgtt

721 acataactta cggtaaatgg cccgcctggc tgaccgccca acgacccccg cccattgacg

781 tcaataatga cgtatgttcc catagtaacg ccaataggga ctttccattg acgtcaatgg

841 gtggagtatt tacggtaaac tgcccacttg gcagtacatc aagtgtatca tatgccaagt

901 acgcccccta ttgacgtcaa tgacggtaaa tggcccgcct ggcattatgc ccagtacatg

961 accttatggg actttcctac ttggcagtac atctacgtat tagtcatcgc tattaccatg

1021 gtgatgcggt tttggcagta catcaatggg cgtggatagc ggtttgactc acggggattt

1081 ccaagtctcc accccattga cgtcaatggg agtttgtttt ggcaccaaaa tcaacgggac

1141 tttccaaaat gtcgtaacaa ctccgcccca ttgacgcaaa tgggcggtag gcgtgtacgg

1201 tgggaggtct atataagcag agctggttta gtgaaccgtc agatcgcctg gtaatacgac

1261 tcactatagg gactagtaag cttgcggccg catccggagg cgggggctcg ggaggtggcg

1321 gctctggagg aggcggtagt accggtgcag aggcagccgc taaggaagcg gctgcaaaag

1381 aggctgctgc gaaggaagca gctgccaagg cccttgaagc agaggctgca gccaaagaag

1441 ctgcagccaa ggaggctgct gcaaaagaag cagctgcaaa agctcccggg ggaggtggca

1501 gtggtggagg cggatcaggt ggaggcggta gtgccggcaa gcttcgatcc agacatgata

1561 agatacattg atgagtttgg acaaaccaca actagaatgc agtgaaaaaa atgctttatt

1621 tgtgaaattt gtgatgctat tgctttattt gtaaccatta taagctgcaa taaacaagtt

1681 aacaacaaca attgcattca ttttatgttt caggttcagg gggaggtgtg ggaggttttt

1741 taaagcaagt aaaacctcta caaatgtggt atggctgatt atgatccggc tgcctcgcgc

1801 gtttcggtga tgacggtgaa aacctctgac acatgcagct cccggagacg gtcacagctt

1861 gtctgtaagc ggatgccggg agcagacaag cccgtcaggg cgcgtcagcg ggtgttggcg

1921 ggtgtcgggg cgcagccatg acccagtcac gtagcgatag cggagtgtat actggcttaa

1981 ctatgcggca tcagagcaga ttgtactgag agtgcaccat atgtcgggcc gcgttgctgg

2041 cgtttttcca taggctccgc ccccctgacg agcatcacaa aaatcgacgc tcaagtcaga

2101 ggtggcgaaa cccgacagga ctataaagat accaggcgtt tccccctgga agctccctcg

2161 tgcgctctcc tgttccgacc ctgccgctta ccggatacct gtccgccttt ctcccttcgg

2221 gaagcgtggc gctttctcat agctcacgct gtaggtatct cagttcggtg taggtcgttc

2281 gctccaagct gggctgtgtg cacgaacccc ccgttcagcc cgaccgctgc gccttatccg

2341 gtaactatcg tcttgagtcc aacccggtaa gacacgactt atcgccactg gcagcagcca

2401 ctggtaacag gattagcaga gcgaggtatg taggcggtgc tacagagttc ttgaagtggt

2461 ggcctaacta cggctacact agaaggacag tatttggtat ctgcgctctg ctgaagccag

2521 ttaccttcgg aaaaagagtt ggtagctctt gatccggcaa acaaaccacc gctggtagcg

2581 gtggtttttt tgtttgcaag cagcagatta cgcgcagaaa aaaaggatct caagaagatc

2641 ctttgatctt ttctacgggg tctgacgctc agtggaacga aaactcacgt taagggattt

2701 tggtcatgag attatcaaaa aggatcttca cctagatcct tttaaattaa aaatgaagtt

2761 ttaaatcaat ctaaagtata tatgagtaaa cttggtctga cagttaccaa tgcttaatca

2821 gtgaggcacc tatctcagcg atctgtctat ttcgttcatc catagttgcc tgactccccg

2881 tcgtgtagat aactacgata cgggagggct taccatctgg ccccagtgct gcaatgatac

2941 cgcgagaccc acgctcaccg gctccagatt tatcagcaat aaaccagcca gccggaaggg

3001 ccgagcgcag aagtggtcct gcaactttat ccgcctccat ccagtctatt aattgttgcc

3061 gggaagctag agtaagtagt tcgccagtta atagtttgcg caacgttgtt gccattgcta

3121 caggcatcgt ggtgtcacgc tcgtcgtttg gtatggcttc attcagctcc ggttcccaac

3181 gatcaaggcg agttacatga tcccccatgt tgtgcaaaaa agcggttagc tccttcggtc

3241 ctccgatcgt tgtcagaagt aagttggccg cagtgttatc actcatggtt atggcagcac

3301 tgcataattc tcttactgtc atgccatccg taagatgctt ttctgtgact ggtgagtact

3361 caaccaagtc attctgagaa tagtgtatgc ggcgaccgag ttgctcttgc ccggcgtcaa

3421 cacgggataa taccgcgcca catagcagaa ctttaaaagt gctcatcatt ggaaaacgtt

3481 cttcggggcg aaaactctca aggatcttac cgctgttgag atccagttcg atgtaaccca

3541 ctcgtgcacc caactgatct tcagcatctt ttactttcac cagcgtttct gggtgagcaa

3601 aaacaggaag gcaaaatgcc gcaaaaaagg gaataagggc gacacggaaa tgttgaatac

3661 tcatactctt cctttttcaa tattattgaa gcatttatca gggttattgt ctcatgagcg

3721 gatacatatt tgaatgtatt tagaaaaata aacaaatagg ggttccgcgc acatttcccc

3781 gaaaagtgcc acctgacgtc taagaaacca ttattatcat gacattaacc tataaaaata

3841 ggcgtatcac gaggcccttt cgtcttcaag aattggtcga tcgaccaatt ctcatgtttg

3901 acagcttatc atcgata

//

**CA97.62:**

LOCUS CA97.62 4423 bp DNA circular SYN 08-JUL-2021

DEFINITION synthetic circular DNA

ACCESSION .

VERSION .

KEYWORDS .

SOURCE synthetic DNA construct

ORGANISM synthetic DNA construct

REFERENCE 1 (bases 1 to 4423)

AUTHORS .

TITLE .

JOURNAL .

FEATURES Location/Qualifiers

source 1..4423

/organism="synthetic DNA construct"

/mol_type="other DNA"

promoter 111..440

/label=SV40 promoter

/note="SV40 enhancer and early promoter"

rep_origin 291..426

/label=SV40 ori

/note="SV40 origin of replication"

CDS 544..819

/codon_start=1

/label=GAL DBD "Mut1"

/translation="MKLLSSIEQACDICRLKKLKCDQEFPSCKRCAKNNWECRYSPETK RSPLTRAHLTEVESRLERLEQLFLLIFPREDLDMILKMDSLQDIKAL"

CDS 829..873

/codon_start=1

/label=3x GGGGS

/translation="GGGGSGGGGSGGGGS"

CDS 880..1023

/codon_start=1

/label=8xEAAAK

/translation="AEAAAKEAAAKEAAAKEAAAKALEAEAAAKEAAAKEAAAKEAAAK APG"

CDS 1039..1800

/codon_start=1

/label=GR LBD CS1/CD 540

/translation="PAALPQLTPTLVSLLEVIEPEVLYAGYDSSVPDSAWRIMTTLNML GGRQVIAAVKWAKAIPGFRNLHLDDQMTLLQYSWMFLMAFALGWRSYRQSSGNLLCFAP DLIINEQRMSLPCMYDQCKHMLFVSSELQRLQVSYEEYLCMKTLLLLSSVPKEGLKSQE LFDEIRMTYIKELGKAIVKREGNSSQNWQRFYQLTKLLDSMHEVVENLLTYCFQTFLDK TMSIEFPEAAAEIITNQIYSNGNIKKLLFHQK"

CDS 1805..1849

/codon_start=1

/label=3x GGGGS

/translation="GEVAVVEADQVEAVV"

CDS 1894..2127

/codon_start=1

/gene="UL48"

/product="transcriptional activation domain of herpes

simplex virus protein VP16 (Triezenberg et al., 1988;

Cousens et al., 1989)"

/label=VP16 AD

/translation="APPTDVSLGDELHLDGEDVAMAHADALDDFDLDMLGDGDSPGPGF TPHDSAPYGALDMADFEFEQMFTDALGIDEYGG"

polyA_signal 2144..2278

/label=SV40 poly(A) signal

/note="SV40 polyadenylation signal"

rep_origin complement(2643..3231)

/direction=LEFT

/label=ori

/note="high-copy-number ColE1/pMB1/pBR322/pUC origin of

replication"

CDS complement(3402..4262)

/codon_start=1

/gene="bla"

/product="beta-lactamase"

/label=AmpR

/note="confers resistance to ampicillin, carbenicillin, and related antibiotics"

/translation="MSIQHFRVALIPFFAAFCLPVFAHPETLVKVKDAEDQLGARVGYI ELDLNSGKILESFRPEERFPMMSTFKVLLCGAVLSRVDAGQEQLGRRIHYSQNDLVEYS PVTEKHLTDGMTVRELCSAAITMSDNTAANLLLTTIGGPKELTAFLHNMGDHVTRLDRW EPELNEAIPNDERDTTMPVAMATTLRKLLTGELLTLASRQQLIDWMEADKVAGPLLRSA LPAGWFIADKSGAGERGSRGIIAALGPDGKPSRIVVIYTTGSQATMDERNRQIAEIGAS LIKHW"

promoter complement(4263..4367)

/gene="bla"

/label=AmpR promoter

ORIGIN

1 taacctataa aaataggcgt atcacgaggc cctttcgtct tcaagaattg gtcgatcgac

61 caattctcat gtttgacagc ttatcatcga taagctagct tctgtggaat gtgtgtcagt

121 tagggtgtgg aaagtcccca ggctccccag caggcagaag tatgcaaagc atgcatctca

181 attagtcagc aaccaggtgt ggaaagtccc caggctcccc agcaggcaga agtatgcaaa

241 gcatgcatct caattagtca gcaaccatag tcccgcccct aactccgccc atcccgcccc

301 taactccgcc cagttccgcc cattctccgc cccatggctg actaattttt tttatttatg

361 cagaggccga ggccgcctcg gcctctgagc tattccagaa gtagtgagga ggcttttttg

421 gaggcctagg cttttgcaaa aagctccctc gaggaactgg aaaaccagaa agttaactgg

481 taagtttagt ctttttgtct tttatttcag gtcccggatc gaattgcggc cgcatccggg

541 accatgaagc tactgtcttc tatcgaacaa gcatgcgata tttgccgact taaaaagctc

601 aagtgcgatc aagaattccc gagctgcaag aggtgtgcga agaacaactg ggagtgtcgc

661 tactctcccg agaccaaaag gtctccgctg actagggcac atctgacaga agtggaatca

721 aggctagaaa gactggaaca gctatttcta ctgatttttc ctcgagaaga ccttgacatg

781 attttgaaaa tggattcttt acaggatata aaagcattgt tgggtgccgg aggcgggggc

841 tcgggaggtg gcggctctgg aggaggcggt agtaccggtg cagaggcagc cgctaaggaa

901 gcggctgcaa aagaggctgc tgcgaaggaa gcagctgcca aggcccttga agcagaggct

961 gcagccaaag aagctgcagc caaggaggct gctgcaaaag aagcagctgc aaaagctccc

1021 gggaccatgt tgggtacccc tgcagcatta ccacagctca cccctacctt ggtgtcactg

1081 ctggaggtga ttgaacccga ggtgttgtat gcaggatatg atagctctgt tccagattca

1141 gcatggagaa ttatgaccac actcaacatg ttaggtgggc gtcaagtgat tgcagcagtg

1201 aaatgggcaa aggcgatacc aggcttcaga aacttacacc tggatgacca aatgaccctg

1261 ctacagtact catggatgtt tctcatggca tttgccctgg gttggagatc atacagacaa

1321 tcaagtggaa acctgctctg ctttgctcct gatctgatta ttaatgagca gagaatgtct

1381 ctaccctgca tgtatgacca atgtaaacac atgctgtttg tctcctctga attacaaaga

1441 ttgcaggtat cctatgaaga gtatctctgt atgaaaacct tactgcttct ctcctcagtt

1501 cctaaggaag gtctgaagag ccaagagtta tttgatgaga ttcgaatgac ttatatcaaa

1561 gagctaggaa aagccatcgt caaaagggaa gggaactcca gtcagaactg gcaacggttt

1621 taccaactga caaagcttct ggactccatg catgaggtgg ttgagaatct ccttacctac

1681 tgcttccaga catttttgga taagaccatg agtattgaat tcccagaggc ggccgctgaa

1741 atcatcacta atcagatata ttcaaatgga aatatcaaaa agcttctgtt tcatcagaag

1801 gccgggggag gtggcagtgg tggaggcgga tcaggtggag gcggtagtgc cggtacccct

1861 gcagctgcgt cgacccaatt cccggggatc tgggcccccc cgaccgatgt cagcctgggg

1921 gacgagctcc acttagacgg cgaggacgtg gcgatggcgc atgccgacgc gctagacgat

1981 ttcgatctgg acatgttggg ggacggggat tccccgggtc cgggatttac cccccacgac

2041 tccgccccct acggcgctct ggatatggcc gacttcgagt ttgagcagat gtttaccgat

2101 gcccttggaa ttgacgagta cggtgggtag accggcaagc ttcgatccag acatgataag

2161 atacattgat gagtttggac aaaccacaac tagaatgcag tgaaaaaaat gctttatttg

2221 tgaaatttgt gatgctattg ctttatttgt aaccattata agctgcaata aacaagttaa

2281 caacaacaat tgcattcatt ttatgtttca ggttcagggg gaggtgtggg aggtttttta

2341 aagcaagtaa aacctctaca aatgtggtat ggctgattat gatccggctg cctcgcgcgt

2401 ttcggtgatg acggtgaaaa cctctgacac atgcagctcc cggagacggt cacagcttgt

2461 ctgtaagcgg atgccgggag cagacaagcc cgtcagggcg cgtcagcggg tgttggcggg

2521 tgtcggggcg cagccatgac ccagtcacgt agcgatagcg gagtgtatac tggcttaact

2581 atgcggcatc agagcagatt gtactgagag tgcaccatat gtcgggccgc gttgctggcg

2641 tttttccata ggctccgccc ccctgacgag catcacaaaa atcgacgctc aagtcagagg

2701 tggcgaaacc cgacaggact ataaagatac caggcgtttc cccctggaag ctccctcgtg

2761 cgctctcctg ttccgaccct gccgcttacc ggatacctgt ccgcctttct cccttcggga

2821 agcgtggcgc tttctcatag ctcacgctgt aggtatctca gttcggtgta ggtcgttcgc

2881 tccaagctgg gctgtgtgca cgaacccccc gttcagcccg accgctgcgc cttatccggt

2941 aactatcgtc ttgagtccaa cccggtaaga cacgacttat cgccactggc agcagccact

3001 ggtaacagga ttagcagagc gaggtatgta ggcggtgcta cagagttctt gaagtggtgg

3061 cctaactacg gctacactag aaggacagta tttggtatct gcgctctgct gaagccagtt

3121 accttcggaa aaagagttgg tagctcttga tccggcaaac aaaccaccgc tggtagcggt

3181 ggtttttttg tttgcaagca gcagattacg cgcagaaaaa aaggatctca agaagatcct

3241 ttgatctttt ctacggggtc tgacgctcag tggaacgaaa actcacgtta agggattttg

3301 gtcatgagat tatcaaaaag gatcttcacc tagatccttt taaattaaaa atgaagtttt

3361 aaatcaatct aaagtatata tgagtaaact tggtctgaca gttaccaatg cttaatcagt

3421 gaggcaccta tctcagcgat ctgtctattt cgttcatcca tagttgcctg actccccgtc

3481 gtgtagataa ctacgatacg ggagggctta ccatctggcc ccagtgctgc aatgataccg

3541 cgagacccac gctcaccggc tccagattta tcagcaataa accagccagc cggaagggcc

3601 gagcgcagaa gtggtcctgc aactttatcc gcctccatcc agtctattaa ttgttgccgg

3661 gaagctagag taagtagttc gccagttaat agtttgcgca acgttgttgc cattgctaca

3721 ggcatcgtgg tgtcacgctc gtcgtttggt atggcttcat tcagctccgg ttcccaacga

3781 tcaaggcgag ttacatgatc ccccatgttg tgcaaaaaag cggttagctc cttcggtcct

3841 ccgatcgttg tcagaagtaa gttggccgca gtgttatcac tcatggttat ggcagcactg

3901 cataattctc ttactgtcat gccatccgta agatgctttt ctgtgactgg tgagtactca

3961 accaagtcat tctgagaata gtgtatgcgg cgaccgagtt gctcttgccc ggcgtcaaca

4021 cgggataata ccgcgccaca tagcagaact ttaaaagtgc tcatcattgg aaaacgttct

4081 tcggggcgaa aactctcaag gatcttaccg ctgttgagat ccagttcgat gtaacccact

4141 cgtgcaccca actgatcttc agcatctttt actttcacca gcgtttctgg gtgagcaaaa

4201 acaggaaggc aaaatgccgc aaaaaaggga ataagggcga cacggaaatg ttgaatactc

4261 atactcttcc tttttcaata ttattgaagc atttatcagg gttattgtct catgagcgga

4321 tacatatttg aatgtattta gaaaaataaa caaatagggg ttccgcgcac atttccccga

4381 aaagtgccac ctgacgtcta agaaaccatt attatcatga cat

//

**CA97.63:**

LOCUS CA97.63 4423 bp DNA circular SYN 08-JUL-2021

DEFINITION synthetic circular DNA

ACCESSION .

VERSION .

KEYWORDS .

SOURCE synthetic DNA construct

ORGANISM synthetic DNA construct

REFERENCE 1 (bases 1 to 4423)

AUTHORS .

TITLE .

JOURNAL .

FEATURES Location/Qualifiers

source 1..4423

/organism="synthetic DNA construct"

/mol_type="other DNA"

promoter 111..440

/label=SV40 promoter

/note="SV40 enhancer and early promoter"

rep_origin 291..426

/label=SV40 ori

/note="SV40 origin of replication"

CDS 544..819

/codon_start=1

/label=GAL DBD "Mut2"

/translation="MKLLSSIEQACDICRLKKLKCDQEFPSCKRCAKNNWECRYSPKTE ESPLTRAHLTEVESRLERLEQLFLLIFPREDLDMILKMDSLQDIKAL"

CDS 829..873

/codon_start=1

/label=3x GGGGS

/translation="GGGGSGGGGSGGGGS"

CDS 880..1023

/codon_start=1

/label=8xEAAAK

/translation="AEAAAKEAAAKEAAAKEAAAKALEAEAAAKEAAAKEAAAKEAAAK APG"

CDS 1039..1800

/codon_start=1

/label=GR LBD CS1/CD 540

/translation="PAALPQLTPTLVSLLEVIEPEVLYAGYDSSVPDSAWRIMTTLNML GGRQVIAAVKWAKAIPGFRNLHLDDQMTLLQYSWMFLMAFALGWRSYRQSSGNLLCFAP DLIINEQRMSLPCMYDQCKHMLFVSSELQRLQVSYEEYLCMKTLLLLSSVPKEGLKSQE LFDEIRMTYIKELGKAIVKREGNSSQNWQRFYQLTKLLDSMHEVVENLLTYCFQTFLDK TMSIEFPEAAAEIITNQIYSNGNIKKLLFHQK"

CDS 1805..1849

/codon_start=1

/label=3x GGGGS

/translation="GEVAVVEADQVEAVV"

CDS 1894..2127

/codon_start=1

/gene="UL48"

/product="transcriptional activation domain of herpes

simplex virus protein VP16 (Triezenberg et al., 1988;

Cousens et al., 1989)"

/label=VP16 AD

/translation="APPTDVSLGDELHLDGEDVAMAHADALDDFDLDMLGDGDSPGPGF TPHDSAPYGALDMADFEFEQMFTDALGIDEYGG"

polyA_signal 2144..2278

/label=SV40 poly(A) signal

/note="SV40 polyadenylation signal"

rep_origin complement(2643..3231)

/direction=LEFT

/label=ori

/note="high-copy-number ColE1/pMB1/pBR322/pUC origin of

replication"

CDS complement(3402..4262)

/codon_start=1

/gene="bla"

/product="beta-lactamase"

/label=AmpR

/note="confers resistance to ampicillin, carbenicillin, and related antibiotics"

/translation="MSIQHFRVALIPFFAAFCLPVFAHPETLVKVKDAEDQLGARVGYI ELDLNSGKILESFRPEERFPMMSTFKVLLCGAVLSRVDAGQEQLGRRIHYSQNDLVEYS PVTEKHLTDGMTVRELCSAAITMSDNTAANLLLTTIGGPKELTAFLHNMGDHVTRLDRW EPELNEAIPNDERDTTMPVAMATTLRKLLTGELLTLASRQQLIDWMEADKVAGPLLRSA LPAGWFIADKSGAGERGSRGIIAALGPDGKPSRIVVIYTTGSQATMDERNRQIAEIGAS LIKHW"

promoter complement(4263..4367)

/gene="bla"

/label=AmpR promoter

ORIGIN

1 taacctataa aaataggcgt atcacgaggc cctttcgtct tcaagaattg gtcgatcgac

61 caattctcat gtttgacagc ttatcatcga taagctagct tctgtggaat gtgtgtcagt

121 tagggtgtgg aaagtcccca ggctccccag caggcagaag tatgcaaagc atgcatctca

181 attagtcagc aaccaggtgt ggaaagtccc caggctcccc agcaggcaga agtatgcaaa

241 gcatgcatct caattagtca gcaaccatag tcccgcccct aactccgccc atcccgcccc

301 taactccgcc cagttccgcc cattctccgc cccatggctg actaattttt tttatttatg

361 cagaggccga ggccgcctcg gcctctgagc tattccagaa gtagtgagga ggcttttttg

421 gaggcctagg cttttgcaaa aagctccctc gaggaactgg aaaaccagaa agttaactgg

481 taagtttagt ctttttgtct tttatttcag gtcccggatc gaattgcggc cgcatccggg

541 accatgaagc tactgtcttc tatcgaacaa gcatgcgata tttgccgact taaaaagctc

601 aagtgcgatc aagaattccc gagctgcaag aggtgtgcga agaacaactg ggagtgtcgc

661 tactctccca aaaccgaaga gtctccgctg actagggcac atctgacaga agtggaatca

721 aggctagaaa gactggaaca gctatttcta ctgatttttc ctcgagaaga ccttgacatg

781 attttgaaaa tggattcttt acaggatata aaagcattgt tgggtgccgg aggcgggggc

841 tcgggaggtg gcggctctgg aggaggcggt agtaccggtg cagaggcagc cgctaaggaa

901 gcggctgcaa aagaggctgc tgcgaaggaa gcagctgcca aggcccttga agcagaggct

961 gcagccaaag aagctgcagc caaggaggct gctgcaaaag aagcagctgc aaaagctccc

1021 gggaccatgt tgggtacccc tgcagcatta ccacagctca cccctacctt ggtgtcactg

1081 ctggaggtga ttgaacccga ggtgttgtat gcaggatatg atagctctgt tccagattca

1141 gcatggagaa ttatgaccac actcaacatg ttaggtgggc gtcaagtgat tgcagcagtg

1201 aaatgggcaa aggcgatacc aggcttcaga aacttacacc tggatgacca aatgaccctg

1261 ctacagtact catggatgtt tctcatggca tttgccctgg gttggagatc atacagacaa

1321 tcaagtggaa acctgctctg ctttgctcct gatctgatta ttaatgagca gagaatgtct

1381 ctaccctgca tgtatgacca atgtaaacac atgctgtttg tctcctctga attacaaaga

1441 ttgcaggtat cctatgaaga gtatctctgt atgaaaacct tactgcttct ctcctcagtt

1501 cctaaggaag gtctgaagag ccaagagtta tttgatgaga ttcgaatgac ttatatcaaa

1561 gagctaggaa aagccatcgt caaaagggaa gggaactcca gtcagaactg gcaacggttt

1621 taccaactga caaagcttct ggactccatg catgaggtgg ttgagaatct ccttacctac

1681 tgcttccaga catttttgga taagaccatg agtattgaat tcccagaggc ggccgctgaa

1741 atcatcacta atcagatata ttcaaatgga aatatcaaaa agcttctgtt tcatcagaag

1801 gccgggggag gtggcagtgg tggaggcgga tcaggtggag gcggtagtgc cggtacccct

1861 gcagctgcgt cgacccaatt cccggggatc tgggcccccc cgaccgatgt cagcctgggg

1921 gacgagctcc acttagacgg cgaggacgtg gcgatggcgc atgccgacgc gctagacgat

1981 ttcgatctgg acatgttggg ggacggggat tccccgggtc cgggatttac cccccacgac

2041 tccgccccct acggcgctct ggatatggcc gacttcgagt ttgagcagat gtttaccgat

2101 gcccttggaa ttgacgagta cggtgggtag accggcaagc ttcgatccag acatgataag

2161 atacattgat gagtttggac aaaccacaac tagaatgcag tgaaaaaaat gctttatttg

2221 tgaaatttgt gatgctattg ctttatttgt aaccattata agctgcaata aacaagttaa

2281 caacaacaat tgcattcatt ttatgtttca ggttcagggg gaggtgtggg aggtttttta

2341 aagcaagtaa aacctctaca aatgtggtat ggctgattat gatccggctg cctcgcgcgt

2401 ttcggtgatg acggtgaaaa cctctgacac atgcagctcc cggagacggt cacagcttgt

2461 ctgtaagcgg atgccgggag cagacaagcc cgtcagggcg cgtcagcggg tgttggcggg

2521 tgtcggggcg cagccatgac ccagtcacgt agcgatagcg gagtgtatac tggcttaact

2581 atgcggcatc agagcagatt gtactgagag tgcaccatat gtcgggccgc gttgctggcg

2641 tttttccata ggctccgccc ccctgacgag catcacaaaa atcgacgctc aagtcagagg

2701 tggcgaaacc cgacaggact ataaagatac caggcgtttc cccctggaag ctccctcgtg

2761 cgctctcctg ttccgaccct gccgcttacc ggatacctgt ccgcctttct cccttcggga

2821 agcgtggcgc tttctcatag ctcacgctgt aggtatctca gttcggtgta ggtcgttcgc

2881 tccaagctgg gctgtgtgca cgaacccccc gttcagcccg accgctgcgc cttatccggt

2941 aactatcgtc ttgagtccaa cccggtaaga cacgacttat cgccactggc agcagccact

3001 ggtaacagga ttagcagagc gaggtatgta ggcggtgcta cagagttctt gaagtggtgg

3061 cctaactacg gctacactag aaggacagta tttggtatct gcgctctgct gaagccagtt

3121 accttcggaa aaagagttgg tagctcttga tccggcaaac aaaccaccgc tggtagcggt

3181 ggtttttttg tttgcaagca gcagattacg cgcagaaaaa aaggatctca agaagatcct

3241 ttgatctttt ctacggggtc tgacgctcag tggaacgaaa actcacgtta agggattttg

3301 gtcatgagat tatcaaaaag gatcttcacc tagatccttt taaattaaaa atgaagtttt

3361 aaatcaatct aaagtatata tgagtaaact tggtctgaca gttaccaatg cttaatcagt

3421 gaggcaccta tctcagcgat ctgtctattt cgttcatcca tagttgcctg actccccgtc

3481 gtgtagataa ctacgatacg ggagggctta ccatctggcc ccagtgctgc aatgataccg

3541 cgagacccac gctcaccggc tccagattta tcagcaataa accagccagc cggaagggcc

3601 gagcgcagaa gtggtcctgc aactttatcc gcctccatcc agtctattaa ttgttgccgg

3661 gaagctagag taagtagttc gccagttaat agtttgcgca acgttgttgc cattgctaca

3721 ggcatcgtgg tgtcacgctc gtcgtttggt atggcttcat tcagctccgg ttcccaacga

3781 tcaaggcgag ttacatgatc ccccatgttg tgcaaaaaag cggttagctc cttcggtcct

3841 ccgatcgttg tcagaagtaa gttggccgca gtgttatcac tcatggttat ggcagcactg

3901 cataattctc ttactgtcat gccatccgta agatgctttt ctgtgactgg tgagtactca

3961 accaagtcat tctgagaata gtgtatgcgg cgaccgagtt gctcttgccc ggcgtcaaca

4021 cgggataata ccgcgccaca tagcagaact ttaaaagtgc tcatcattgg aaaacgttct

4081 tcggggcgaa aactctcaag gatcttaccg ctgttgagat ccagttcgat gtaacccact

4141 cgtgcaccca actgatcttc agcatctttt actttcacca gcgtttctgg gtgagcaaaa

4201 acaggaaggc aaaatgccgc aaaaaaggga ataagggcga cacggaaatg ttgaatactc

4261 atactcttcc tttttcaata ttattgaagc atttatcagg gttattgtct catgagcgga

4321 tacatatttg aatgtattta gaaaaataaa caaatagggg ttccgcgcac atttccccga

4381 aaagtgccac ctgacgtcta agaaaccatt attatcatga cat

//

**CA97.64:**

LOCUS CA97.64 4423 bp DNA circular SYN 08-JUL-2021

DEFINITION synthetic circular DNA

ACCESSION .

VERSION .

KEYWORDS .

SOURCE synthetic DNA construct

ORGANISM synthetic DNA construct

REFERENCE 1 (bases 1 to 4423)

AUTHORS .

TITLE .

JOURNAL .

FEATURES Location/Qualifiers

source 1..4423

/organism="synthetic DNA construct"

/mol_type="other DNA"

promoter 111..440

/label=SV40 promoter

/note="SV40 enhancer and early promoter"

rep_origin 291..426

/label=SV40 ori

/note="SV40 origin of replication"

CDS 544..819

/codon_start=1

/label=GAL DBD "Mut3"

/translation="MKLLSSIEQACDICRLKKLKCDQEFPSCKRCAKNNWECRYSPKTE ESPLTRAHLTEVESRLERLEQLFLLIFPREDLDMILKMDSLQDIKAL"

CDS 829..873

/codon_start=1

/label=3x GGGGS

/translation="GGGGSGGGGSGGGGS"

CDS 880..1023

/codon_start=1

/label=8xEAAAK

/translation="AEAAAKEAAAKEAAAKEAAAKALEAEAAAKEAAAKEAAAKEAAAK APG"

CDS 1039..1800

/codon_start=1

/label=GR LBD CS1/CD 540

/translation="PAALPQLTPTLVSLLEVIEPEVLYAGYDSSVPDSAWRIMTTLNML GGRQVIAAVKWAKAIPGFRNLHLDDQMTLLQYSWMFLMAFALGWRSYRQSSGNLLCFAP DLIINEQRMSLPCMYDQCKHMLFVSSELQRLQVSYEEYLCMKTLLLLSSVPKEGLKSQE LFDEIRMTYIKELGKAIVKREGNSSQNWQRFYQLTKLLDSMHEVVENLLTYCFQTFLDK TMSIEFPEAAAEIITNQIYSNGNIKKLLFHQK"

CDS 1805..1849

/codon_start=1

/label=3x GGGGS

/translation="GEVAVVEADQVEAVV"

CDS 1894..2127

/codon_start=1

/gene="UL48"

/product="transcriptional activation domain of herpes

simplex virus protein VP16 (Triezenberg et al., 1988;

Cousens et al., 1989)"

/label=VP16 AD

/translation="APPTDVSLGDELHLDGEDVAMAHADALDDFDLDMLGDGDSPGPGF TPHDSAPYGALDMADFEFEQMFTDALGIDEYGG"

polyA_signal 2144..2278

/label=SV40 poly(A) signal

/note="SV40 polyadenylation signal"

rep_origin complement(2643..3231)

/direction=LEFT

/label=ori

/note="high-copy-number ColE1/pMB1/pBR322/pUC origin of

replication"

CDS complement(3402..4262)

/codon_start=1

/gene="bla"

/product="beta-lactamase"

/label=AmpR

/note="confers resistance to ampicillin, carbenicillin, and related antibiotics"

/translation="MSIQHFRVALIPFFAAFCLPVFAHPETLVKVKDAEDQLGARVGYI ELDLNSGKILESFRPEERFPMMSTFKVLLCGAVLSRVDAGQEQLGRRIHYSQNDLVEYS PVTEKHLTDGMTVRELCSAAITMSDNTAANLLLTTIGGPKELTAFLHNMGDHVTRLDRW EPELNEAIPNDERDTTMPVAMATTLRKLLTGELLTLASRQQLIDWMEADKVAGPLLRSA LPAGWFIADKSGAGERGSRGIIAALGPDGKPSRIVVIYTTGSQATMDERNRQIAEIGAS LIKHW"

promoter complement(4263..4367)

/gene="bla"

/label=AmpR promoter

ORIGIN

1 taacctataa aaataggcgt atcacgaggc cctttcgtct tcaagaattg gtcgatcgac

61 caattctcat gtttgacagc ttatcatcga taagctagct tctgtggaat gtgtgtcagt

121 tagggtgtgg aaagtcccca ggctccccag caggcagaag tatgcaaagc atgcatctca

181 attagtcagc aaccaggtgt ggaaagtccc caggctcccc agcaggcaga agtatgcaaa

241 gcatgcatct caattagtca gcaaccatag tcccgcccct aactccgccc atcccgcccc

301 taactccgcc cagttccgcc cattctccgc cccatggctg actaattttt tttatttatg

361 cagaggccga ggccgcctcg gcctctgagc tattccagaa gtagtgagga ggcttttttg

421 gaggcctagg cttttgcaaa aagctccctc gaggaactgg aaaaccagaa agttaactgg

481 taagtttagt ctttttgtct tttatttcag gtcccggatc gaattgcggc cgcatccggg

541 accatgaagc tactgtcttc tatcgaacaa gcatgcgata tttgccgact taaaaagctc

601 aagtgcgccc aagaattccc gagctgcaag aggtgtgcga agaacaactg ggagtgtcgc

661 tactctcccg agaccaaaag gtctccgctg actagggcac atctgacaga agtggaatca

721 aggctagaaa gactggaaca gctatttcta ctgatttttc ctcgagaaga ccttgacatg

781 attttgaaaa tggattcttt acaggatata aaagcattgt tgggtgccgg aggcgggggc

841 tcgggaggtg gcggctctgg aggaggcggt agtaccggtg cagaggcagc cgctaaggaa

901 gcggctgcaa aagaggctgc tgcgaaggaa gcagctgcca aggcccttga agcagaggct

961 gcagccaaag aagctgcagc caaggaggct gctgcaaaag aagcagctgc aaaagctccc

1021 gggaccatgt tgggtacccc tgcagcatta ccacagctca cccctacctt ggtgtcactg

1081 ctggaggtga ttgaacccga ggtgttgtat gcaggatatg atagctctgt tccagattca

1141 gcatggagaa ttatgaccac actcaacatg ttaggtgggc gtcaagtgat tgcagcagtg

1201 aaatgggcaa aggcgatacc aggcttcaga aacttacacc tggatgacca aatgaccctg

1261 ctacagtact catggatgtt tctcatggca tttgccctgg gttggagatc atacagacaa

1321 tcaagtggaa acctgctctg ctttgctcct gatctgatta ttaatgagca gagaatgtct

1381 ctaccctgca tgtatgacca atgtaaacac atgctgtttg tctcctctga attacaaaga

1441 ttgcaggtat cctatgaaga gtatctctgt atgaaaacct tactgcttct ctcctcagtt

1501 cctaaggaag gtctgaagag ccaagagtta tttgatgaga ttcgaatgac ttatatcaaa

1561 gagctaggaa aagccatcgt caaaagggaa gggaactcca gtcagaactg gcaacggttt

1621 taccaactga caaagcttct ggactccatg catgaggtgg ttgagaatct ccttacctac

1681 tgcttccaga catttttgga taagaccatg agtattgaat tcccagaggc ggccgctgaa

1741 atcatcacta atcagatata ttcaaatgga aatatcaaaa agcttctgtt tcatcagaag

1801 gccgggggag gtggcagtgg tggaggcgga tcaggtggag gcggtagtgc cggtacccct

1861 gcagctgcgt cgacccaatt cccggggatc tgggcccccc cgaccgatgt cagcctgggg

1921 gacgagctcc acttagacgg cgaggacgtg gcgatggcgc atgccgacgc gctagacgat

1981 ttcgatctgg acatgttggg ggacggggat tccccgggtc cgggatttac cccccacgac

2041 tccgccccct acggcgctct ggatatggcc gacttcgagt ttgagcagat gtttaccgat

2101 gcccttggaa ttgacgagta cggtgggtag accggcaagc ttcgatccag acatgataag

2161 atacattgat gagtttggac aaaccacaac tagaatgcag tgaaaaaaat gctttatttg

2221 tgaaatttgt gatgctattg ctttatttgt aaccattata agctgcaata aacaagttaa

2281 caacaacaat tgcattcatt ttatgtttca ggttcagggg gaggtgtggg aggtttttta

2341 aagcaagtaa aacctctaca aatgtggtat ggctgattat gatccggctg cctcgcgcgt

2401 ttcggtgatg acggtgaaaa cctctgacac atgcagctcc cggagacggt cacagcttgt

2461 ctgtaagcgg atgccgggag cagacaagcc cgtcagggcg cgtcagcggg tgttggcggg

2521 tgtcggggcg cagccatgac ccagtcacgt agcgatagcg gagtgtatac tggcttaact

2581 atgcggcatc agagcagatt gtactgagag tgcaccatat gtcgggccgc gttgctggcg

2641 tttttccata ggctccgccc ccctgacgag catcacaaaa atcgacgctc aagtcagagg

2701 tggcgaaacc cgacaggact ataaagatac caggcgtttc cccctggaag ctccctcgtg

2761 cgctctcctg ttccgaccct gccgcttacc ggatacctgt ccgcctttct cccttcggga

2821 agcgtggcgc tttctcatag ctcacgctgt aggtatctca gttcggtgta ggtcgttcgc

2881 tccaagctgg gctgtgtgca cgaacccccc gttcagcccg accgctgcgc cttatccggt

2941 aactatcgtc ttgagtccaa cccggtaaga cacgacttat cgccactggc agcagccact

3001 ggtaacagga ttagcagagc gaggtatgta ggcggtgcta cagagttctt gaagtggtgg

3061 cctaactacg gctacactag aaggacagta tttggtatct gcgctctgct gaagccagtt

3121 accttcggaa aaagagttgg tagctcttga tccggcaaac aaaccaccgc tggtagcggt

3181 ggtttttttg tttgcaagca gcagattacg cgcagaaaaa aaggatctca agaagatcct

3241 ttgatctttt ctacggggtc tgacgctcag tggaacgaaa actcacgtta agggattttg

3301 gtcatgagat tatcaaaaag gatcttcacc tagatccttt taaattaaaa atgaagtttt

3361 aaatcaatct aaagtatata tgagtaaact tggtctgaca gttaccaatg cttaatcagt

3421 gaggcaccta tctcagcgat ctgtctattt cgttcatcca tagttgcctg actccccgtc

3481 gtgtagataa ctacgatacg ggagggctta ccatctggcc ccagtgctgc aatgataccg

3541 cgagacccac gctcaccggc tccagattta tcagcaataa accagccagc cggaagggcc

3601 gagcgcagaa gtggtcctgc aactttatcc gcctccatcc agtctattaa ttgttgccgg

3661 gaagctagag taagtagttc gccagttaat agtttgcgca acgttgttgc cattgctaca

3721 ggcatcgtgg tgtcacgctc gtcgtttggt atggcttcat tcagctccgg ttcccaacga

3781 tcaaggcgag ttacatgatc ccccatgttg tgcaaaaaag cggttagctc cttcggtcct

3841 ccgatcgttg tcagaagtaa gttggccgca gtgttatcac tcatggttat ggcagcactg

3901 cataattctc ttactgtcat gccatccgta agatgctttt ctgtgactgg tgagtactca

3961 accaagtcat tctgagaata gtgtatgcgg cgaccgagtt gctcttgccc ggcgtcaaca

4021 cgggataata ccgcgccaca tagcagaact ttaaaagtgc tcatcattgg aaaacgttct

4081 tcggggcgaa aactctcaag gatcttaccg ctgttgagat ccagttcgat gtaacccact

4141 cgtgcaccca actgatcttc agcatctttt actttcacca gcgtttctgg gtgagcaaaa

4201 acaggaaggc aaaatgccgc aaaaaaggga ataagggcga cacggaaatg ttgaatactc

4261 atactcttcc tttttcaata ttattgaagc atttatcagg gttattgtct catgagcgga

4321 tacatatttg aatgtattta gaaaaataaa caaatagggg ttccgcgcac atttccccga

4381 aaagtgccac ctgacgtcta agaaaccatt attatcatga cat

//

**CA94.65:**

LOCUS CA97.65 4423 bp DNA circular SYN 08-JUL-2021

DEFINITION synthetic circular DNA

ACCESSION .

VERSION .

KEYWORDS .

SOURCE synthetic DNA construct

ORGANISM synthetic DNA construct

REFERENCE 1 (bases 1 to 4423)

AUTHORS .

TITLE .

JOURNAL .

FEATURES Location/Qualifiers

source 1..4423

/organism="synthetic DNA construct"

/mol_type="other DNA"

promoter 111..440

/label=SV40 promoter

/note="SV40 enhancer and early promoter"

rep_origin 291..426

/label=SV40 ori

/note="SV40 origin of replication"

CDS 544..819

/codon_start=1

/label=GAL DBD "Mut4"

/translation="MKLLSSIEQACDICRLKKLKCAQEFPSCKRCAKNNWECRYSPKTE ESPLTRAHLTEVESRLERLEQLFLLIFPREDLDMILKMDSLQDIKAL"

CDS 829..873

/codon_start=1

/label=3x GGGGS

/translation="GGGGSGGGGSGGGGS"

CDS 880..1023

/codon_start=1

/label=8xEAAAK

/translation="AEAAAKEAAAKEAAAKEAAAKALEAEAAAKEAAAKEAAAKEAAAK APG"

CDS 1039..1800

/codon_start=1

/label=GR LBD CS1/CD 540

/translation="PAALPQLTPTLVSLLEVIEPEVLYAGYDSSVPDSAWRIMTTLNML GGRQVIAAVKWAKAIPGFRNLHLDDQMTLLQYSWMFLMAFALGWRSYRQSSGNLLCFAP DLIINEQRMSLPCMYDQCKHMLFVSSELQRLQVSYEEYLCMKTLLLLSSVPKEGLKSQE LFDEIRMTYIKELGKAIVKREGNSSQNWQRFYQLTKLLDSMHEVVENLLTYCFQTFLDK TMSIEFPEAAAEIITNQIYSNGNIKKLLFHQK"

CDS 1805..1849

/codon_start=1

/label=3x GGGGS

/translation="GEVAVVEADQVEAVV"

CDS 1894..2127

/codon_start=1

/gene="UL48"

/product="transcriptional activation domain of herpes

simplex virus protein VP16 (Triezenberg et al., 1988;

Cousens et al., 1989)"

/label=VP16 AD

/translation="APPTDVSLGDELHLDGEDVAMAHADALDDFDLDMLGDGDSPGPGF TPHDSAPYGALDMADFEFEQMFTDALGIDEYGG"

polyA_signal 2144..2278

/label=SV40 poly(A) signal

/note="SV40 polyadenylation signal"

rep_origin complement(2643..3231)

/direction=LEFT

/label=ori

/note="high-copy-number ColE1/pMB1/pBR322/pUC origin of

replication"

CDS complement(3402..4262)

/codon_start=1

/gene="bla"

/product="beta-lactamase"

/label=AmpR

/note="confers resistance to ampicillin, carbenicillin, and related antibiotics"

/translation="MSIQHFRVALIPFFAAFCLPVFAHPETLVKVKDAEDQLGARVGYI ELDLNSGKILESFRPEERFPMMSTFKVLLCGAVLSRVDAGQEQLGRRIHYSQNDLVEYS PVTEKHLTDGMTVRELCSAAITMSDNTAANLLLTTIGGPKELTAFLHNMGDHVTRLDRW EPELNEAIPNDERDTTMPVAMATTLRKLLTGELLTLASRQQLIDWMEADKVAGPLLRSA LPAGWFIADKSGAGERGSRGIIAALGPDGKPSRIVVIYTTGSQATMDERNRQIAEIGAS LIKHW"

promoter complement(4263..4367)

/gene="bla"

/label=AmpR promoter

ORIGIN

1 taacctataa aaataggcgt atcacgaggc cctttcgtct tcaagaattg gtcgatcgac

61 caattctcat gtttgacagc ttatcatcga taagctagct tctgtggaat gtgtgtcagt

121 tagggtgtgg aaagtcccca ggctccccag caggcagaag tatgcaaagc atgcatctca

181 attagtcagc aaccaggtgt ggaaagtccc caggctcccc agcaggcaga agtatgcaaa

241 gcatgcatct caattagtca gcaaccatag tcccgcccct aactccgccc atcccgcccc

301 taactccgcc cagttccgcc cattctccgc cccatggctg actaattttt tttatttatg

361 cagaggccga ggccgcctcg gcctctgagc tattccagaa gtagtgagga ggcttttttg

421 gaggcctagg cttttgcaaa aagctccctc gaggaactgg aaaaccagaa agttaactgg

481 taagtttagt ctttttgtct tttatttcag gtcccggatc gaattgcggc cgcatccggg

541 accatgaagc tactgtcttc tatcgaacaa gcatgcgata tttgccgact taaaaagctc

601 aagtgcgccc aagaattccc gagctgcaag aggtgtgcga agaacaactg ggagtgtcgc

661 tactctccca aaaccgaaga gtctccgctg actagggcac atctgacaga agtggaatca

721 aggctagaaa gactggaaca gctatttcta ctgatttttc ctcgagaaga ccttgacatg

781 attttgaaaa tggattcttt acaggatata aaagcattgt tgggtgccgg aggcgggggc

841 tcgggaggtg gcggctctgg aggaggcggt agtaccggtg cagaggcagc cgctaaggaa

901 gcggctgcaa aagaggctgc tgcgaaggaa gcagctgcca aggcccttga agcagaggct

961 gcagccaaag aagctgcagc caaggaggct gctgcaaaag aagcagctgc aaaagctccc

1021 gggaccatgt tgggtacccc tgcagcatta ccacagctca cccctacctt ggtgtcactg

1081 ctggaggtga ttgaacccga ggtgttgtat gcaggatatg atagctctgt tccagattca

1141 gcatggagaa ttatgaccac actcaacatg ttaggtgggc gtcaagtgat tgcagcagtg

1201 aaatgggcaa aggcgatacc aggcttcaga aacttacacc tggatgacca aatgaccctg

1261 ctacagtact catggatgtt tctcatggca tttgccctgg gttggagatc atacagacaa

1321 tcaagtggaa acctgctctg ctttgctcct gatctgatta ttaatgagca gagaatgtct

1381 ctaccctgca tgtatgacca atgtaaacac atgctgtttg tctcctctga attacaaaga

1441 ttgcaggtat cctatgaaga gtatctctgt atgaaaacct tactgcttct ctcctcagtt

1501 cctaaggaag gtctgaagag ccaagagtta tttgatgaga ttcgaatgac ttatatcaaa

1561 gagctaggaa aagccatcgt caaaagggaa gggaactcca gtcagaactg gcaacggttt

1621 taccaactga caaagcttct ggactccatg catgaggtgg ttgagaatct ccttacctac

1681 tgcttccaga catttttgga taagaccatg agtattgaat tcccagaggc ggccgctgaa

1741 atcatcacta atcagatata ttcaaatgga aatatcaaaa agcttctgtt tcatcagaag

1801 gccgggggag gtggcagtgg tggaggcgga tcaggtggag gcggtagtgc cggtacccct

1861 gcagctgcgt cgacccaatt cccggggatc tgggcccccc cgaccgatgt cagcctgggg

1921 gacgagctcc acttagacgg cgaggacgtg gcgatggcgc atgccgacgc gctagacgat

1981 ttcgatctgg acatgttggg ggacggggat tccccgggtc cgggatttac cccccacgac

2041 tccgccccct acggcgctct ggatatggcc gacttcgagt ttgagcagat gtttaccgat

2101 gcccttggaa ttgacgagta cggtgggtag accggcaagc ttcgatccag acatgataag

2161 atacattgat gagtttggac aaaccacaac tagaatgcag tgaaaaaaat gctttatttg

2221 tgaaatttgt gatgctattg ctttatttgt aaccattata agctgcaata aacaagttaa

2281 caacaacaat tgcattcatt ttatgtttca ggttcagggg gaggtgtggg aggtttttta

2341 aagcaagtaa aacctctaca aatgtggtat ggctgattat gatccggctg cctcgcgcgt

2401 ttcggtgatg acggtgaaaa cctctgacac atgcagctcc cggagacggt cacagcttgt

2461 ctgtaagcgg atgccgggag cagacaagcc cgtcagggcg cgtcagcggg tgttggcggg

2521 tgtcggggcg cagccatgac ccagtcacgt agcgatagcg gagtgtatac tggcttaact

2581 atgcggcatc agagcagatt gtactgagag tgcaccatat gtcgggccgc gttgctggcg

2641 tttttccata ggctccgccc ccctgacgag catcacaaaa atcgacgctc aagtcagagg

2701 tggcgaaacc cgacaggact ataaagatac caggcgtttc cccctggaag ctccctcgtg

2761 cgctctcctg ttccgaccct gccgcttacc ggatacctgt ccgcctttct cccttcggga

2821 agcgtggcgc tttctcatag ctcacgctgt aggtatctca gttcggtgta ggtcgttcgc

2881 tccaagctgg gctgtgtgca cgaacccccc gttcagcccg accgctgcgc cttatccggt

2941 aactatcgtc ttgagtccaa cccggtaaga cacgacttat cgccactggc agcagccact

3001 ggtaacagga ttagcagagc gaggtatgta ggcggtgcta cagagttctt gaagtggtgg

3061 cctaactacg gctacactag aaggacagta tttggtatct gcgctctgct gaagccagtt

3121 accttcggaa aaagagttgg tagctcttga tccggcaaac aaaccaccgc tggtagcggt

3181 ggtttttttg tttgcaagca gcagattacg cgcagaaaaa aaggatctca agaagatcct

3241 ttgatctttt ctacggggtc tgacgctcag tggaacgaaa actcacgtta agggattttg

3301 gtcatgagat tatcaaaaag gatcttcacc tagatccttt taaattaaaa atgaagtttt

3361 aaatcaatct aaagtatata tgagtaaact tggtctgaca gttaccaatg cttaatcagt

3421 gaggcaccta tctcagcgat ctgtctattt cgttcatcca tagttgcctg actccccgtc

3481 gtgtagataa ctacgatacg ggagggctta ccatctggcc ccagtgctgc aatgataccg

3541 cgagacccac gctcaccggc tccagattta tcagcaataa accagccagc cggaagggcc

3601 gagcgcagaa gtggtcctgc aactttatcc gcctccatcc agtctattaa ttgttgccgg

3661 gaagctagag taagtagttc gccagttaat agtttgcgca acgttgttgc cattgctaca

3721 ggcatcgtgg tgtcacgctc gtcgtttggt atggcttcat tcagctccgg ttcccaacga

3781 tcaaggcgag ttacatgatc ccccatgttg tgcaaaaaag cggttagctc cttcggtcct

3841 ccgatcgttg tcagaagtaa gttggccgca gtgttatcac tcatggttat ggcagcactg

3901 cataattctc ttactgtcat gccatccgta agatgctttt ctgtgactgg tgagtactca

3961 accaagtcat tctgagaata gtgtatgcgg cgaccgagtt gctcttgccc ggcgtcaaca

4021 cgggataata ccgcgccaca tagcagaact ttaaaagtgc tcatcattgg aaaacgttct

4081 tcggggcgaa aactctcaag gatcttaccg ctgttgagat ccagttcgat gtaacccact

4141 cgtgcaccca actgatcttc agcatctttt actttcacca gcgtttctgg gtgagcaaaa

4201 acaggaaggc aaaatgccgc aaaaaaggga ataagggcga cacggaaatg ttgaatactc

4261 atactcttcc tttttcaata ttattgaagc atttatcagg gttattgtct catgagcgga

4321 tacatatttg aatgtattta gaaaaataaa caaatagggg ttccgcgcac atttccccga

4381 aaagtgccac ctgacgtcta agaaaccatt attatcatga cat

//

**CA97.66:**

LOCUS CA97.66 4423 bp DNA circular SYN 06-JUL-2021

DEFINITION synthetic circular DNA

ACCESSION .

VERSION .

KEYWORDS .

SOURCE synthetic DNA construct

ORGANISM synthetic DNA construct

REFERENCE 1 (bases 1 to 4423)

AUTHORS .

TITLE .

JOURNAL .

FEATURES Location/Qualifiers

source 1..4423

/organism="synthetic DNA construct"

/mol_type="other DNA"

promoter 111..440

/label=SV40 promoter

/note="SV40 enhancer and early promoter"

rep_origin 291..426

/label=SV40 ori

/note="SV40 origin of replication"

CDS 544..819

/label=Gal4 DBD "Mut5"

CDS 829..873

/codon_start=1

/label=3x GGGGS

/translation="GGGGSGGGGSGGGGS"

CDS 880..1023

/codon_start=1

/label=8xEAAAK

/translation="AEAAAKEAAAKEAAAKEAAAKALEAEAAAKEAAAKEAAAKEAAAK APG"

CDS 1039..1800

/codon_start=1

/label=GR LBD CS1/CD 540

/translation="PAALPQLTPTLVSLLEVIEPEVLYAGYDSSVPDSAWRIMTTLNML GGRQVIAAVKWAKAIPGFRNLHLDDQMTLLQYSWMFLMAFALGWRSYRQSSGNLLCFAP DLIINEQRMSLPCMYDQCKHMLFVSSELQRLQVSYEEYLCMKTLLLLSSVPKEGLKSQE LFDEIRMTYIKELGKAIVKREGNSSQNWQRFYQLTKLLDSMHEVVENLLTYCFQTFLDK TMSIEFPEAAAEIITNQIYSNGNIKKLLFHQK"

CDS 1805..1849

/label=3xGGGS

CDS 1894..2127

/codon_start=1

/gene="UL48"

/product="transcriptional activation domain of herpes

simplex virus protein VP16 (Triezenberg et al., 1988;

Cousens et al., 1989)"

/label=VP16 AD

/translation="APPTDVSLGDELHLDGEDVAMAHADALDDFDLDMLGDGDSPGPGF TPHDSAPYGALDMADFEFEQMFTDALGIDEYGG"

polyA_signal 2144..2278

/label=SV40 poly(A) signal

/note="SV40 polyadenylation signal"

rep_origin complement(2643..3231)

/direction=LEFT

/label=ori

/note="high-copy-number ColE1/pMB1/pBR322/pUC origin of

replication"

CDS complement(3402..4262)

/codon_start=1

/gene="bla"

/product="beta-lactamase"

/label=AmpR

/note="confers resistance to ampicillin, carbenicillin, and related antibiotics"

/translation="MSIQHFRVALIPFFAAFCLPVFAHPETLVKVKDAEDQLGARVGYI ELDLNSGKILESFRPEERFPMMSTFKVLLCGAVLSRVDAGQEQLGRRIHYSQNDLVEYS PVTEKHLTDGMTVRELCSAAITMSDNTAANLLLTTIGGPKELTAFLHNMGDHVTRLDRW EPELNEAIPNDERDTTMPVAMATTLRKLLTGELLTLASRQQLIDWMEADKVAGPLLRSA LPAGWFIADKSGAGERGSRGIIAALGPDGKPSRIVVIYTTGSQATMDERNRQIAEIGAS LIKHW"

promoter complement(4263..4367)

/gene="bla"

/label=AmpR promoter

ORIGIN

1 taacctataa aaataggcgt atcacgaggc cctttcgtct tcaagaattg gtcgatcgac

61 caattctcat gtttgacagc ttatcatcga taagctagct tctgtggaat gtgtgtcagt

121 tagggtgtgg aaagtcccca ggctccccag caggcagaag tatgcaaagc atgcatctca

181 attagtcagc aaccaggtgt ggaaagtccc caggctcccc agcaggcaga agtatgcaaa

241 gcatgcatct caattagtca gcaaccatag tcccgcccct aactccgccc atcccgcccc

301 taactccgcc cagttccgcc cattctccgc cccatggctg actaattttt tttatttatg

361 cagaggccga ggccgcctcg gcctctgagc tattccagaa gtagtgagga ggcttttttg

421 gaggcctagg cttttgcaaa aagctccctc gaggaactgg aaaaccagaa agttaactgg

481 taagtttagt ctttttgtct tttatttcag gtcccggatc gaattgcggc cgcatccggg

541 accatgaagc tactgtctgc tatcgaacaa gcatgcgata tttgccgact taaaaagctc

601 aagtgctcca aagaaaaacc gaagtgcgcc aagtgtctga agaacaactg ggagtgtcgc

661 cactctcccg aaaccaaaag gtctccgctg actagggcac atctgacaga agtggaatca

721 aggctagaaa gactggaaca gctatttcta ctgatttttc ctcgagaaga ccttgacatg

781 attttgaaaa tggattcttt acaggatata aaagcattgt tgggtgccgg aggcgggggc

841 tcgggaggtg gcggctctgg aggaggcggt agtaccggtg cagaggcagc cgctaaggaa

901 gcggctgcaa aagaggctgc tgcgaaggaa gcagctgcca aggcccttga agcagaggct

961 gcagccaaag aagctgcagc caaggaggct gctgcaaaag aagcagctgc aaaagctccc

1021 gggaccatgt tgggtacccc tgcagcatta ccacagctca cccctacctt ggtgtcactg

1081 ctggaggtga ttgaacccga ggtgttgtat gcaggatatg atagctctgt tccagattca

1141 gcatggagaa ttatgaccac actcaacatg ttaggtgggc gtcaagtgat tgcagcagtg

1201 aaatgggcaa aggcgatacc aggcttcaga aacttacacc tggatgacca aatgaccctg

1261 ctacagtact catggatgtt tctcatggca tttgccctgg gttggagatc atacagacaa

1321 tcaagtggaa acctgctctg ctttgctcct gatctgatta ttaatgagca gagaatgtct

1381 ctaccctgca tgtatgacca atgtaaacac atgctgtttg tctcctctga attacaaaga

1441 ttgcaggtat cctatgaaga gtatctctgt atgaaaacct tactgcttct ctcctcagtt

1501 cctaaggaag gtctgaagag ccaagagtta tttgatgaga ttcgaatgac ttatatcaaa

1561 gagctaggaa aagccatcgt caaaagggaa gggaactcca gtcagaactg gcaacggttt

1621 taccaactga caaagcttct ggactccatg catgaggtgg ttgagaatct ccttacctac

1681 tgcttccaga catttttgga taagaccatg agtattgaat tcccagaggc ggccgctgaa

1741 atcatcacta atcagatata ttcaaatgga aatatcaaaa agcttctgtt tcatcagaag

1801 gccgggggag gtggcagtgg tggaggcgga tcaggtggag gcggtagtgc cggtacccct

1861 gcagctgcgt cgacccaatt cccggggatc tgggcccccc cgaccgatgt cagcctgggg

1921 gacgagctcc acttagacgg cgaggacgtg gcgatggcgc atgccgacgc gctagacgat

1981 ttcgatctgg acatgttggg ggacggggat tccccgggtc cgggatttac cccccacgac

2041 tccgccccct acggcgctct ggatatggcc gacttcgagt ttgagcagat gtttaccgat

2101 gcccttggaa ttgacgagta cggtgggtag accggcaagc ttcgatccag acatgataag

2161 atacattgat gagtttggac aaaccacaac tagaatgcag tgaaaaaaat gctttatttg

2221 tgaaatttgt gatgctattg ctttatttgt aaccattata agctgcaata aacaagttaa

2281 caacaacaat tgcattcatt ttatgtttca ggttcagggg gaggtgtggg aggtttttta

2341 aagcaagtaa aacctctaca aatgtggtat ggctgattat gatccggctg cctcgcgcgt

2401 ttcggtgatg acggtgaaaa cctctgacac atgcagctcc cggagacggt cacagcttgt

2461 ctgtaagcgg atgccgggag cagacaagcc cgtcagggcg cgtcagcggg tgttggcggg

2521 tgtcggggcg cagccatgac ccagtcacgt agcgatagcg gagtgtatac tggcttaact

2581 atgcggcatc agagcagatt gtactgagag tgcaccatat gtcgggccgc gttgctggcg

2641 tttttccata ggctccgccc ccctgacgag catcacaaaa atcgacgctc aagtcagagg

2701 tggcgaaacc cgacaggact ataaagatac caggcgtttc cccctggaag ctccctcgtg

2761 cgctctcctg ttccgaccct gccgcttacc ggatacctgt ccgcctttct cccttcggga

2821 agcgtggcgc tttctcatag ctcacgctgt aggtatctca gttcggtgta ggtcgttcgc

2881 tccaagctgg gctgtgtgca cgaacccccc gttcagcccg accgctgcgc cttatccggt

2941 aactatcgtc ttgagtccaa cccggtaaga cacgacttat cgccactggc agcagccact

3001 ggtaacagga ttagcagagc gaggtatgta ggcggtgcta cagagttctt gaagtggtgg

3061 cctaactacg gctacactag aaggacagta tttggtatct gcgctctgct gaagccagtt

3121 accttcggaa aaagagttgg tagctcttga tccggcaaac aaaccaccgc tggtagcggt

3181 ggtttttttg tttgcaagca gcagattacg cgcagaaaaa aaggatctca agaagatcct

3241 ttgatctttt ctacggggtc tgacgctcag tggaacgaaa actcacgtta agggattttg

3301 gtcatgagat tatcaaaaag gatcttcacc tagatccttt taaattaaaa atgaagtttt

3361 aaatcaatct aaagtatata tgagtaaact tggtctgaca gttaccaatg cttaatcagt

3421 gaggcaccta tctcagcgat ctgtctattt cgttcatcca tagttgcctg actccccgtc

3481 gtgtagataa ctacgatacg ggagggctta ccatctggcc ccagtgctgc aatgataccg

3541 cgagacccac gctcaccggc tccagattta tcagcaataa accagccagc cggaagggcc

3601 gagcgcagaa gtggtcctgc aactttatcc gcctccatcc agtctattaa ttgttgccgg

3661 gaagctagag taagtagttc gccagttaat agtttgcgca acgttgttgc cattgctaca

3721 ggcatcgtgg tgtcacgctc gtcgtttggt atggcttcat tcagctccgg ttcccaacga

3781 tcaaggcgag ttacatgatc ccccatgttg tgcaaaaaag cggttagctc cttcggtcct

3841 ccgatcgttg tcagaagtaa gttggccgca gtgttatcac tcatggttat ggcagcactg

3901 cataattctc ttactgtcat gccatccgta agatgctttt ctgtgactgg tgagtactca

3961 accaagtcat tctgagaata gtgtatgcgg cgaccgagtt gctcttgccc ggcgtcaaca

4021 cgggataata ccgcgccaca tagcagaact ttaaaagtgc tcatcattgg aaaacgttct

4081 tcggggcgaa aactctcaag gatcttaccg ctgttgagat ccagttcgat gtaacccact

4141 cgtgcaccca actgatcttc agcatctttt actttcacca gcgtttctgg gtgagcaaaa

4201 acaggaaggc aaaatgccgc aaaaaaggga ataagggcga cacggaaatg ttgaatactc

4261 atactcttcc tttttcaata ttattgaagc atttatcagg gttattgtct catgagcgga

4321 tacatatttg aatgtattta gaaaaataaa caaatagggg ttccgcgcac atttccccga

4381 aaagtgccac ctgacgtcta agaaaccatt attatcatga cat

//

**CA97.67:**

LOCUS CA97.67 4423 bp DNA circular SYN 08-JUL-2021

DEFINITION synthetic circular DNA

ACCESSION .

VERSION .

KEYWORDS .

SOURCE synthetic DNA construct

ORGANISM synthetic DNA construct

REFERENCE 1 (bases 1 to 4423)

AUTHORS .

TITLE .

JOURNAL .

FEATURES Location/Qualifiers

source 1..4423

/organism="synthetic DNA construct"

/mol_type="other DNA"

promoter 111..440

/label=SV40 promoter

/note="SV40 enhancer and early promoter"

rep_origin 291..426

/label=SV40 ori

/note="SV40 origin of replication"

CDS 544..819

/codon_start=1

/label=GAL DBD WT

/translation="MKLLSSIEQACDICRLKKLKCSKEKPKCAKCLKNNWECRYSPKTK RSPLTRAHLTEVESRLERLEQLFLLIFPREDLDMILKMDSLQDIKAL"

CDS 829..873

/codon_start=1

/label=3x GGGGS

/translation="GGGGSGGGGSGGGGS"

CDS 880..1023

/codon_start=1

/label=8xEAAAK

/translation="AEAAAKEAAAKEAAAKEAAAKALEAEAAAKEAAAKEAAAKEAAAK APG"

CDS 1039..1800

/codon_start=1

/label=GR LBD CS1/CD 540

/translation="PAALPQLTPTLVSLLEVIEPEVLYAGYDSSVPDSAWRIMTTLNML GGRQVIAAVKWAKAIPGFRNLHLDDQMTLLQYSWMFLMAFALGWRSYRQSSGNLLCFAP DLIINEQRMSLPCMYDQCKHMLFVSSELQRLQVSYEEYLCMKTLLLLSSVPKEGLKSQE LFDEIRMTYIKELGKAIVKREGNSSQNWQRFYQLTKLLDSMHEVVENLLTYCFQTFLDK TMSIEFPEAAAEIITNQIYSNGNIKKLLFHQK"

CDS 1805..1849

/codon_start=1

/label=3x GGGGS

/translation="GEVAVVEADQVEAVV"

CDS 1894..2127

/codon_start=1

/gene="UL48"

/product="transcriptional activation domain of herpes

simplex virus protein VP16 (Triezenberg et al., 1988;

Cousens et al., 1989)"

/label=VP16 AD

/translation="APPTDVSLGDELHLDGEDVAMAHADALDDFDLDMLGDGDSPGPGF TPHDSAPYGALDMADFEFEQMFTDALGIDEYGG"

polyA_signal 2144..2278

/label=SV40 poly(A) signal

/note="SV40 polyadenylation signal"

rep_origin complement(2643..3231)

/direction=LEFT

/label=ori

/note="high-copy-number ColE1/pMB1/pBR322/pUC origin of

replication"

CDS complement(3402..4262)

/codon_start=1

/gene="bla"

/product="beta-lactamase"

/label=AmpR

/note="confers resistance to ampicillin, carbenicillin, and related antibiotics"

/translation="MSIQHFRVALIPFFAAFCLPVFAHPETLVKVKDAEDQLGARVGYI ELDLNSGKILESFRPEERFPMMSTFKVLLCGAVLSRVDAGQEQLGRRIHYSQNDLVEYS PVTEKHLTDGMTVRELCSAAITMSDNTAANLLLTTIGGPKELTAFLHNMGDHVTRLDRW EPELNEAIPNDERDTTMPVAMATTLRKLLTGELLTLASRQQLIDWMEADKVAGPLLRSA LPAGWFIADKSGAGERGSRGIIAALGPDGKPSRIVVIYTTGSQATMDERNRQIAEIGAS LIKHW"

promoter complement(4263..4367)

/gene="bla"

/label=AmpR promoter

ORIGIN

1 taacctataa aaataggcgt atcacgaggc cctttcgtct tcaagaattg gtcgatcgac

61 caattctcat gtttgacagc ttatcatcga taagctagct tctgtggaat gtgtgtcagt

121 tagggtgtgg aaagtcccca ggctccccag caggcagaag tatgcaaagc atgcatctca

181 attagtcagc aaccaggtgt ggaaagtccc caggctcccc agcaggcaga agtatgcaaa

241 gcatgcatct caattagtca gcaaccatag tcccgcccct aactccgccc atcccgcccc

301 taactccgcc cagttccgcc cattctccgc cccatggctg actaattttt tttatttatg

361 cagaggccga ggccgcctcg gcctctgagc tattccagaa gtagtgagga ggcttttttg

421 gaggcctagg cttttgcaaa aagctccctc gaggaactgg aaaaccagaa agttaactgg

481 taagtttagt ctttttgtct tttatttcag gtcccggatc gaattgcggc cgcatccggg

541 accatgaagc tactgtcttc tatcgaacaa gcatgcgata tttgccgact taaaaagctc

601 aagtgctcca aagaaaaacc gaagtgcgcc aagtgtctga agaacaactg ggagtgtcgc

661 tactctccca aaaccaaaag gtctccgctg actagggcac atctgacaga agtggaatca

721 aggctagaaa gactggaaca gctatttcta ctgatttttc ctcgagaaga ccttgacatg

781 attttgaaaa tggattcttt acaggatata aaagcattgt tgggtgccgg aggcgggggc

841 tcgggaggtg gcggctctgg aggaggcggt agtaccggtg cagaggcagc cgctaaggaa

901 gcggctgcaa aagaggctgc tgcgaaggaa gcagctgcca aggcccttga agcagaggct

961 gcagccaaag aagctgcagc caaggaggct gctgcaaaag aagcagctgc aaaagctccc

1021 gggaccatgt tgggtacccc tgcagcatta ccacagctca cccctacctt ggtgtcactg

1081 ctggaggtga ttgaacccga ggtgttgtat gcaggatatg atagctctgt tccagattca

1141 gcatggagaa ttatgaccac actcaacatg ttaggtgggc gtcaagtgat tgcagcagtg

1201 aaatgggcaa aggcgatacc aggcttcaga aacttacacc tggatgacca aatgaccctg

1261 ctacagtact catggatgtt tctcatggca tttgccctgg gttggagatc atacagacaa

1321 tcaagtggaa acctgctctg ctttgctcct gatctgatta ttaatgagca gagaatgtct

1381 ctaccctgca tgtatgacca atgtaaacac atgctgtttg tctcctctga attacaaaga

1441 ttgcaggtat cctatgaaga gtatctctgt atgaaaacct tactgcttct ctcctcagtt

1501 cctaaggaag gtctgaagag ccaagagtta tttgatgaga ttcgaatgac ttatatcaaa

1561 gagctaggaa aagccatcgt caaaagggaa gggaactcca gtcagaactg gcaacggttt

1621 taccaactga caaagcttct ggactccatg catgaggtgg ttgagaatct ccttacctac

1681 tgcttccaga catttttgga taagaccatg agtattgaat tcccagaggc ggccgctgaa

1741 atcatcacta atcagatata ttcaaatgga aatatcaaaa agcttctgtt tcatcagaag

1801 gccgggggag gtggcagtgg tggaggcgga tcaggtggag gcggtagtgc cggtacccct

1861 gcagctgcgt cgacccaatt cccggggatc tgggcccccc cgaccgatgt cagcctgggg

1921 gacgagctcc acttagacgg cgaggacgtg gcgatggcgc atgccgacgc gctagacgat

1981 ttcgatctgg acatgttggg ggacggggat tccccgggtc cgggatttac cccccacgac

2041 tccgccccct acggcgctct ggatatggcc gacttcgagt ttgagcagat gtttaccgat

2101 gcccttggaa ttgacgagta cggtgggtag accggcaagc ttcgatccag acatgataag

2161 atacattgat gagtttggac aaaccacaac tagaatgcag tgaaaaaaat gctttatttg

2221 tgaaatttgt gatgctattg ctttatttgt aaccattata agctgcaata aacaagttaa

2281 caacaacaat tgcattcatt ttatgtttca ggttcagggg gaggtgtggg aggtttttta

2341 aagcaagtaa aacctctaca aatgtggtat ggctgattat gatccggctg cctcgcgcgt

2401 ttcggtgatg acggtgaaaa cctctgacac atgcagctcc cggagacggt cacagcttgt

2461 ctgtaagcgg atgccgggag cagacaagcc cgtcagggcg cgtcagcggg tgttggcggg

2521 tgtcggggcg cagccatgac ccagtcacgt agcgatagcg gagtgtatac tggcttaact

2581 atgcggcatc agagcagatt gtactgagag tgcaccatat gtcgggccgc gttgctggcg

2641 tttttccata ggctccgccc ccctgacgag catcacaaaa atcgacgctc aagtcagagg

2701 tggcgaaacc cgacaggact ataaagatac caggcgtttc cccctggaag ctccctcgtg

2761 cgctctcctg ttccgaccct gccgcttacc ggatacctgt ccgcctttct cccttcggga

2821 agcgtggcgc tttctcatag ctcacgctgt aggtatctca gttcggtgta ggtcgttcgc

2881 tccaagctgg gctgtgtgca cgaacccccc gttcagcccg accgctgcgc cttatccggt

2941 aactatcgtc ttgagtccaa cccggtaaga cacgacttat cgccactggc agcagccact

3001 ggtaacagga ttagcagagc gaggtatgta ggcggtgcta cagagttctt gaagtggtgg

3061 cctaactacg gctacactag aaggacagta tttggtatct gcgctctgct gaagccagtt

3121 accttcggaa aaagagttgg tagctcttga tccggcaaac aaaccaccgc tggtagcggt

3181 ggtttttttg tttgcaagca gcagattacg cgcagaaaaa aaggatctca agaagatcct

3241 ttgatctttt ctacggggtc tgacgctcag tggaacgaaa actcacgtta agggattttg

3301 gtcatgagat tatcaaaaag gatcttcacc tagatccttt taaattaaaa atgaagtttt

3361 aaatcaatct aaagtatata tgagtaaact tggtctgaca gttaccaatg cttaatcagt

3421 gaggcaccta tctcagcgat ctgtctattt cgttcatcca tagttgcctg actccccgtc

3481 gtgtagataa ctacgatacg ggagggctta ccatctggcc ccagtgctgc aatgataccg

3541 cgagacccac gctcaccggc tccagattta tcagcaataa accagccagc cggaagggcc

3601 gagcgcagaa gtggtcctgc aactttatcc gcctccatcc agtctattaa ttgttgccgg

3661 gaagctagag taagtagttc gccagttaat agtttgcgca acgttgttgc cattgctaca

3721 ggcatcgtgg tgtcacgctc gtcgtttggt atggcttcat tcagctccgg ttcccaacga

3781 tcaaggcgag ttacatgatc ccccatgttg tgcaaaaaag cggttagctc cttcggtcct

3841 ccgatcgttg tcagaagtaa gttggccgca gtgttatcac tcatggttat ggcagcactg

3901 cataattctc ttactgtcat gccatccgta agatgctttt ctgtgactgg tgagtactca

3961 accaagtcat tctgagaata gtgtatgcgg cgaccgagtt gctcttgccc ggcgtcaaca

4021 cgggataata ccgcgccaca tagcagaact ttaaaagtgc tcatcattgg aaaacgttct

4081 tcggggcgaa aactctcaag gatcttaccg ctgttgagat ccagttcgat gtaacccact

4141 cgtgcaccca actgatcttc agcatctttt actttcacca gcgtttctgg gtgagcaaaa

4201 acaggaaggc aaaatgccgc aaaaaaggga ataagggcga cacggaaatg ttgaatactc

4261 atactcttcc tttttcaata ttattgaagc atttatcagg gttattgtct catgagcgga

4321 tacatatttg aatgtattta gaaaaataaa caaatagggg ttccgcgcac atttccccga

4381 aaagtgccac ctgacgtcta agaaaccatt attatcatga cat

//

**CA97.75:**

LOCUS CA97.75 4427 bp DNA circular SYN 08-JUL-2021

DEFINITION synthetic circular DNA

ACCESSION .

VERSION .

KEYWORDS .

SOURCE synthetic DNA construct

ORGANISM synthetic DNA construct

REFERENCE 1 (bases 1 to 4427)

AUTHORS .

TITLE .

JOURNAL .

FEATURES Location/Qualifiers

source 1..4427

/organism="synthetic DNA construct"

/mol_type="other DNA"

promoter 113..442

/label=SV40 promoter

/note="SV40 enhancer and early promoter"

rep_origin 293..428

/label=SV40 ori

/note="SV40 origin of replication"

CDS 548..823

/codon_start=1

/label=GAL4 DBD "Mut6"

/translation="MKLLSSIEQACDICRLKKLKCDQEFPSCKRCAKNNWECRYSPKTK RSPLTRAHLTEVESRLERLEQLFLLIFPREDLDMILKMDSLQDIKAL"

CDS 833..877

/codon_start=1

/label=3x GGGGS

/translation="GGGGSGGGGSGGGGS"

CDS 884..1027

/codon_start=1

/label=Feature 9

/translation="AEAAAKEAAAKEAAAKEAAAKALEAEAAAKEAAAKEAAAKEAAAK APG"

CDS 1043..1804

/codon_start=1

/label=GR LBD CS1/CD 540

/translation="PAALPQLTPTLVSLLEVIEPEVLYAGYDSSVPDSAWRIMTTLNML GGRQVIAAVKWAKAIPGFRNLHLDDQMTLLQYSWMFLMAFALGWRSYRQSSGNLLCFAP DLIINEQRMSLPCMYDQCKHMLFVSSELQRLQVSYEEYLCMKTLLLLSSVPKEGLKSQE LFDEIRMTYIKELGKAIVKREGNSSQNWQRFYQLTKLLDSMHEVVENLLTYCFQTFLDK TMSIEFPEAAAEIITNQIYSNGNIKKLLFHQK"

CDS 1809..1853

/codon_start=1

/label=3x GGGGS

/translation="GEVAVVEADQVEAVV"

CDS 1898..2131

/codon_start=1

/gene="UL48"

/product="transcriptional activation domain of herpes

simplex virus protein VP16 (Triezenberg et al., 1988;

Cousens et al., 1989)"

/label=VP16 AD

/translation="APPTDVSLGDELHLDGEDVAMAHADALDDFDLDMLGDGDSPGPGF TPHDSAPYGALDMADFEFEQMFTDALGIDEYGG"

polyA_signal 2148..2282

/label=SV40 poly(A) signal

/note="SV40 polyadenylation signal"

rep_origin complement(2647..3235)

/direction=LEFT

/label=ori

/note="high-copy-number ColE1/pMB1/pBR322/pUC origin of

replication"

CDS complement(3406..4266)

/codon_start=1

/gene="bla"

/product="beta-lactamase"

/label=AmpR

/note="confers resistance to ampicillin, carbenicillin, and related antibiotics"

/translation="MSIQHFRVALIPFFAAFCLPVFAHPETLVKVKDAEDQLGARVGYI ELDLNSGKILESFRPEERFPMMSTFKVLLCGAVLSRVDAGQEQLGRRIHYSQNDLVEYS PVTEKHLTDGMTVRELCSAAITMSDNTAANLLLTTIGGPKELTAFLHNMGDHVTRLDRW EPELNEAIPNDERDTTMPVAMATTLRKLLTGELLTLASRQQLIDWMEADKVAGPLLRSA LPAGWFIADKSGAGERGSRGIIAALGPDGKPSRIVVIYTTGSQATMDERNRQIAEIGAS LIKHW"

promoter complement(4267..4371)

/gene="bla"

/label=AmpR promoter

ORIGIN

1 taacctataa aaataggcgt atcacgaggc cctttcgtct tcaagaattg gtcgatcgac

61 caattctcat gtttgacagc ttatcatcga taagctagct tggctgtgga atgtgtgtca

121 gttagggtgt ggaaagtccc caggctcccc agcaggcaga agtatgcaaa gcatgcatct

181 caattagtca gcaaccaggt gtggaaagtc cccaggctcc ccagcaggca gaagtatgca

241 aagcatgcat ctcaattagt cagcaaccat agtcccgccc ctaactccgc ccatcccgcc

301 cctaactccg cccagttccg cccattctcc gccccatggc tgactaattt tttttattta

361 tgcagaggcc gaggccgcct cggcctctga gctattccag aagtagtgag gaggcttttt

421 tggaggccta ggcttttgca aaaagctcct cgaggaactg aaaaaccaga aagttaactg

481 gtaagtttag tctttttgtc ttttatttca ggtcccggat cggaattgcg cggccgcatc

541 cgggaccatg aagctactgt cttctatcga acaagcatgc gatatttgcc gacttaaaaa

601 gctcaagtgc gatcaagaat tcccgagctg caagaggtgt gcgaagaaca actgggagtg

661 tcgctactct cccaagacca aaaggtctcc gctgactagg gcacatctga cagaagtgga

721 atcaaggcta gaaagactgg aacagctatt tctactgatt tttcctcgag aagaccttga

781 catgattttg aaaatggatt ctttacagga tataaaagca ttgttgggtg ccggaggcgg

841 gggctcggga ggtggcggct ctggaggagg cggtagtacc ggtgcagagg cagccgctaa

901 ggaagcggct gcaaaagagg ctgctgcgaa ggaagcagct gccaaggccc ttgaagcaga

961 ggctgcagcc aaagaagctg cagccaagga ggctgctgca aaagaagcag ctgcaaaagc

1021 tcccgggacc atgttgggta cccctgcagc attaccacag ctcaccccta ccttggtgtc

1081 actgctggag gtgattgaac ccgaggtgtt gtatgcagga tatgatagct ctgttccaga

1141 ttcagcatgg agaattatga ccacactcaa catgttaggt gggcgtcaag tgattgcagc

1201 agtgaaatgg gcaaaggcga taccaggctt cagaaactta cacctggatg accaaatgac

1261 cctgctacag tactcatgga tgtttctcat ggcatttgcc ctgggttgga gatcatacag

1321 acaatcaagt ggaaacctgc tctgctttgc tcctgatctg attattaatg agcagagaat

1381 gtctctaccc tgcatgtatg accaatgtaa acacatgctg tttgtctcct ctgaattaca

1441 aagattgcag gtatcctatg aagagtatct ctgtatgaaa accttactgc ttctctcctc

1501 agttcctaag gaaggtctga agagccaaga gttatttgat gagattcgaa tgacttatat

1561 caaagagcta ggaaaagcca tcgtcaaaag ggaagggaac tccagtcaga actggcaacg

1621 gttttaccaa ctgacaaagc ttctggactc catgcatgag gtggttgaga atctccttac

1681 ctactgcttc cagacatttt tggataagac catgagtatt gaattcccag aggcggccgc

1741 tgaaatcatc actaatcaga tatattcaaa tggaaatatc aaaaagcttc tgtttcatca

1801 gaaggccggg ggaggtggca gtggtggagg cggatcaggt ggaggcggta gtgccggtac

1861 ccctgcagct gcgtcgaccc aattcccggg gatctgggcc cccccgaccg atgtcagcct

1921 gggggacgag ctccacttag acggcgagga cgtggcgatg gcgcatgccg acgcgctaga

1981 cgatttcgat ctggacatgt tgggggacgg ggattccccg ggtccgggat ttacccccca

2041 cgactccgcc ccctacggcg ctctggatat ggccgacttc gagtttgagc agatgtttac

2101 cgatgccctt ggaattgacg agtacggtgg gtagaccggc aagcttcgat ccagacatga

2161 taagatacat tgatgagttt ggacaaacca caactagaat gcagtgaaaa aaatgcttta

2221 tttgtgaaat ttgtgatgct attgctttat ttgtaaccat tataagctgc aataaacaag

2281 ttaacaacaa caattgcatt cattttatgt ttcaggttca gggggaggtg tgggaggttt

2341 tttaaagcaa gtaaaacctc tacaaatgtg gtatggctga ttatgatccg gctgcctcgc

2401 gcgtttcggt gatgacggtg aaaacctctg acacatgcag ctcccggaga cggtcacagc

2461 ttgtctgtaa gcggatgccg ggagcagaca agcccgtcag ggcgcgtcag cgggtgttgg

2521 cgggtgtcgg ggcgcagcca tgacccagtc acgtagcgat agcggagtgt atactggctt

2581 aactatgcgg catcagagca gattgtactg agagtgcacc atatgtcggg ccgcgttgct

2641 ggcgtttttc cataggctcc gcccccctga cgagcatcac aaaaatcgac gctcaagtca

2701 gaggtggcga aacccgacag gactataaag ataccaggcg tttccccctg gaagctccct

2761 cgtgcgctct cctgttccga ccctgccgct taccggatac ctgtccgcct ttctcccttc

2821 gggaagcgtg gcgctttctc atagctcacg ctgtaggtat ctcagttcgg tgtaggtcgt

2881 tcgctccaag ctgggctgtg tgcacgaacc ccccgttcag cccgaccgct gcgccttatc

2941 cggtaactat cgtcttgagt ccaacccggt aagacacgac ttatcgccac tggcagcagc

3001 cactggtaac aggattagca gagcgaggta tgtaggcggt gctacagagt tcttgaagtg

3061 gtggcctaac tacggctaca ctagaaggac agtatttggt atctgcgctc tgctgaagcc

3121 agttaccttc ggaaaaagag ttggtagctc ttgatccggc aaacaaacca ccgctggtag

3181 cggtggtttt tttgtttgca agcagcagat tacgcgcaga aaaaaaggat ctcaagaaga

3241 tcctttgatc ttttctacgg ggtctgacgc tcagtggaac gaaaactcac gttaagggat

3301 tttggtcatg agattatcaa aaaggatctt cacctagatc cttttaaatt aaaaatgaag

3361 ttttaaatca atctaaagta tatatgagta aacttggtct gacagttacc aatgcttaat

3421 cagtgaggca cctatctcag cgatctgtct atttcgttca tccatagttg cctgactccc

3481 cgtcgtgtag ataactacga tacgggaggg cttaccatct ggccccagtg ctgcaatgat

3541 accgcgagac ccacgctcac cggctccaga tttatcagca ataaaccagc cagccggaag

3601 ggccgagcgc agaagtggtc ctgcaacttt atccgcctcc atccagtcta ttaattgttg

3661 ccgggaagct agagtaagta gttcgccagt taatagtttg cgcaacgttg ttgccattgc

3721 tacaggcatc gtggtgtcac gctcgtcgtt tggtatggct tcattcagct ccggttccca

3781 acgatcaagg cgagttacat gatcccccat gttgtgcaaa aaagcggtta gctccttcgg

3841 tcctccgatc gttgtcagaa gtaagttggc cgcagtgtta tcactcatgg ttatggcagc

3901 actgcataat tctcttactg tcatgccatc cgtaagatgc ttttctgtga ctggtgagta

3961 ctcaaccaag tcattctgag aatagtgtat gcggcgaccg agttgctctt gcccggcgtc

4021 aacacgggat aataccgcgc cacatagcag aactttaaaa gtgctcatca ttggaaaacg

4081 ttcttcgggg cgaaaactct caaggatctt accgctgttg agatccagtt cgatgtaacc

4141 cactcgtgca cccaactgat cttcagcatc ttttactttc accagcgttt ctgggtgagc

4201 aaaaacagga aggcaaaatg ccgcaaaaaa gggaataagg gcgacacgga aatgttgaat

4261 actcatactc ttcctttttc aatattattg aagcatttat cagggttatt gtctcatgag

4321 cggatacata tttgaatgta tttagaaaaa taaacaaata ggggttccgc gcacatttcc

4381 ccgaaaagtg ccacctgacg tctaagaaac cattattatc atgacat

//

**CA97.76:**

LOCUS CA97.76 4427 bp DNA circular SYN 08-JUL-2021

DEFINITION synthetic circular DNA

ACCESSION .

VERSION .

KEYWORDS .

SOURCE synthetic DNA construct

ORGANISM synthetic DNA construct

REFERENCE 1 (bases 1 to 4427)

AUTHORS .

TITLE .

JOURNAL .

FEATURES Location/Qualifiers

source 1..4427

/organism="synthetic DNA construct"

/mol_type="other DNA"

promoter 113..442

/label=SV40 promoter

/note="SV40 enhancer and early promoter"

rep_origin 293..428

/label=SV40 ori

/note="SV40 origin of replication"

CDS 548..823

/codon_start=1

/label=GAL4 DBD "Mut7"

/translation="MKLLSSIEQACDICRLKKLKCAQEFPSCKRCAKNNWECRYSPKTK RSPLTRAHLTEVESRLERLEQLFLLIFPREDLDMILKMDSLQDIKAL"

CDS 833..877

/codon_start=1

/label=3x GGGGS

/translation="GGGGSGGGGSGGGGS"

CDS 884..1027

/codon_start=1

/label=Feature 9

/translation="AEAAAKEAAAKEAAAKEAAAKALEAEAAAKEAAAKEAAAKEAAAK APG"

CDS 1043..1804

/codon_start=1

/label=GR LBD CS1/CD 540

/translation="PAALPQLTPTLVSLLEVIEPEVLYAGYDSSVPDSAWRIMTTLNML GGRQVIAAVKWAKAIPGFRNLHLDDQMTLLQYSWMFLMAFALGWRSYRQSSGNLLCFAP DLIINEQRMSLPCMYDQCKHMLFVSSELQRLQVSYEEYLCMKTLLLLSSVPKEGLKSQE LFDEIRMTYIKELGKAIVKREGNSSQNWQRFYQLTKLLDSMHEVVENLLTYCFQTFLDK TMSIEFPEAAAEIITNQIYSNGNIKKLLFHQK"

CDS 1809..1853

/codon_start=1

/label=3x GGGGS

/translation="GEVAVVEADQVEAVV"

CDS 1898..2131

/codon_start=1

/gene="UL48"

/product="transcriptional activation domain of herpes

simplex virus protein VP16 (Triezenberg et al., 1988;

Cousens et al., 1989)"

/label=VP16 AD

/translation="APPTDVSLGDELHLDGEDVAMAHADALDDFDLDMLGDGDSPGPGF TPHDSAPYGALDMADFEFEQMFTDALGIDEYGG"

polyA_signal 2148..2282

/label=SV40 poly(A) signal

/note="SV40 polyadenylation signal"

rep_origin complement(2647..3235)

/direction=LEFT

/label=ori

/note="high-copy-number ColE1/pMB1/pBR322/pUC origin of

replication"

CDS complement(3406..4266)

/codon_start=1

/gene="bla"

/product="beta-lactamase"

/label=AmpR

/note="confers resistance to ampicillin, carbenicillin, and related antibiotics"

/translation="MSIQHFRVALIPFFAAFCLPVFAHPETLVKVKDAEDQLGARVGYI ELDLNSGKILESFRPEERFPMMSTFKVLLCGAVLSRVDAGQEQLGRRIHYSQNDLVEYS PVTEKHLTDGMTVRELCSAAITMSDNTAANLLLTTIGGPKELTAFLHNMGDHVTRLDRW EPELNEAIPNDERDTTMPVAMATTLRKLLTGELLTLASRQQLIDWMEADKVAGPLLRSA LPAGWFIADKSGAGERGSRGIIAALGPDGKPSRIVVIYTTGSQATMDERNRQIAEIGAS LIKHW"

promoter complement(4267..4371)

/gene="bla"

/label=AmpR promoter

ORIGIN

1 taacctataa aaataggcgt atcacgaggc cctttcgtct tcaagaattg gtcgatcgac

61 caattctcat gtttgacagc ttatcatcga taagctagct tggctgtgga atgtgtgtca

121 gttagggtgt ggaaagtccc caggctcccc agcaggcaga agtatgcaaa gcatgcatct

181 caattagtca gcaaccaggt gtggaaagtc cccaggctcc ccagcaggca gaagtatgca

241 aagcatgcat ctcaattagt cagcaaccat agtcccgccc ctaactccgc ccatcccgcc

301 cctaactccg cccagttccg cccattctcc gccccatggc tgactaattt tttttattta

361 tgcagaggcc gaggccgcct cggcctctga gctattccag aagtagtgag gaggcttttt

421 tggaggccta ggcttttgca aaaagctcct cgaggaactg aaaaaccaga aagttaactg

481 gtaagtttag tctttttgtc ttttatttca ggtcccggat cggaattgcg cggccgcatc

541 cgggaccatg aagctactgt cttctatcga acaagcatgc gatatttgcc gacttaaaaa

601 gctcaagtgc gcccaagaat tcccgagctg caagaggtgt gcgaagaaca actgggagtg

661 tcgctactct cccaagacca aaaggtctcc gctgactagg gcacatctga cagaagtgga

721 atcaaggcta gaaagactgg aacagctatt tctactgatt tttcctcgag aagaccttga

781 catgattttg aaaatggatt ctttacagga tataaaagca ttgttgggtg ccggaggcgg

841 gggctcggga ggtggcggct ctggaggagg cggtagtacc ggtgcagagg cagccgctaa

901 ggaagcggct gcaaaagagg ctgctgcgaa ggaagcagct gccaaggccc ttgaagcaga

961 ggctgcagcc aaagaagctg cagccaagga ggctgctgca aaagaagcag ctgcaaaagc

1021 tcccgggacc atgttgggta cccctgcagc attaccacag ctcaccccta ccttggtgtc

1081 actgctggag gtgattgaac ccgaggtgtt gtatgcagga tatgatagct ctgttccaga

1141 ttcagcatgg agaattatga ccacactcaa catgttaggt gggcgtcaag tgattgcagc

1201 agtgaaatgg gcaaaggcga taccaggctt cagaaactta cacctggatg accaaatgac

1261 cctgctacag tactcatgga tgtttctcat ggcatttgcc ctgggttgga gatcatacag

1321 acaatcaagt ggaaacctgc tctgctttgc tcctgatctg attattaatg agcagagaat

1381 gtctctaccc tgcatgtatg accaatgtaa acacatgctg tttgtctcct ctgaattaca

1441 aagattgcag gtatcctatg aagagtatct ctgtatgaaa accttactgc ttctctcctc

1501 agttcctaag gaaggtctga agagccaaga gttatttgat gagattcgaa tgacttatat

1561 caaagagcta ggaaaagcca tcgtcaaaag ggaagggaac tccagtcaga actggcaacg

1621 gttttaccaa ctgacaaagc ttctggactc catgcatgag gtggttgaga atctccttac

1681 ctactgcttc cagacatttt tggataagac catgagtatt gaattcccag aggcggccgc

1741 tgaaatcatc actaatcaga tatattcaaa tggaaatatc aaaaagcttc tgtttcatca

1801 gaaggccggg ggaggtggca gtggtggagg cggatcaggt ggaggcggta gtgccggtac

1861 ccctgcagct gcgtcgaccc aattcccggg gatctgggcc cccccgaccg atgtcagcct

1921 gggggacgag ctccacttag acggcgagga cgtggcgatg gcgcatgccg acgcgctaga

1981 cgatttcgat ctggacatgt tgggggacgg ggattccccg ggtccgggat ttacccccca

2041 cgactccgcc ccctacggcg ctctggatat ggccgacttc gagtttgagc agatgtttac

2101 cgatgccctt ggaattgacg agtacggtgg gtagaccggc aagcttcgat ccagacatga

2161 taagatacat tgatgagttt ggacaaacca caactagaat gcagtgaaaa aaatgcttta

2221 tttgtgaaat ttgtgatgct attgctttat ttgtaaccat tataagctgc aataaacaag

2281 ttaacaacaa caattgcatt cattttatgt ttcaggttca gggggaggtg tgggaggttt

2341 tttaaagcaa gtaaaacctc tacaaatgtg gtatggctga ttatgatccg gctgcctcgc

2401 gcgtttcggt gatgacggtg aaaacctctg acacatgcag ctcccggaga cggtcacagc

2461 ttgtctgtaa gcggatgccg ggagcagaca agcccgtcag ggcgcgtcag cgggtgttgg

2521 cgggtgtcgg ggcgcagcca tgacccagtc acgtagcgat agcggagtgt atactggctt

2581 aactatgcgg catcagagca gattgtactg agagtgcacc atatgtcggg ccgcgttgct

2641 ggcgtttttc cataggctcc gcccccctga cgagcatcac aaaaatcgac gctcaagtca

2701 gaggtggcga aacccgacag gactataaag ataccaggcg tttccccctg gaagctccct

2761 cgtgcgctct cctgttccga ccctgccgct taccggatac ctgtccgcct ttctcccttc

2821 gggaagcgtg gcgctttctc atagctcacg ctgtaggtat ctcagttcgg tgtaggtcgt

2881 tcgctccaag ctgggctgtg tgcacgaacc ccccgttcag cccgaccgct gcgccttatc

2941 cggtaactat cgtcttgagt ccaacccggt aagacacgac ttatcgccac tggcagcagc

3001 cactggtaac aggattagca gagcgaggta tgtaggcggt gctacagagt tcttgaagtg

3061 gtggcctaac tacggctaca ctagaaggac agtatttggt atctgcgctc tgctgaagcc

3121 agttaccttc ggaaaaagag ttggtagctc ttgatccggc aaacaaacca ccgctggtag

3181 cggtggtttt tttgtttgca agcagcagat tacgcgcaga aaaaaaggat ctcaagaaga

3241 tcctttgatc ttttctacgg ggtctgacgc tcagtggaac gaaaactcac gttaagggat

3301 tttggtcatg agattatcaa aaaggatctt cacctagatc cttttaaatt aaaaatgaag

3361 ttttaaatca atctaaagta tatatgagta aacttggtct gacagttacc aatgcttaat

3421 cagtgaggca cctatctcag cgatctgtct atttcgttca tccatagttg cctgactccc

3481 cgtcgtgtag ataactacga tacgggaggg cttaccatct ggccccagtg ctgcaatgat

3541 accgcgagac ccacgctcac cggctccaga tttatcagca ataaaccagc cagccggaag

3601 ggccgagcgc agaagtggtc ctgcaacttt atccgcctcc atccagtcta ttaattgttg

3661 ccgggaagct agagtaagta gttcgccagt taatagtttg cgcaacgttg ttgccattgc

3721 tacaggcatc gtggtgtcac gctcgtcgtt tggtatggct tcattcagct ccggttccca

3781 acgatcaagg cgagttacat gatcccccat gttgtgcaaa aaagcggtta gctccttcgg

3841 tcctccgatc gttgtcagaa gtaagttggc cgcagtgtta tcactcatgg ttatggcagc

3901 actgcataat tctcttactg tcatgccatc cgtaagatgc ttttctgtga ctggtgagta

3961 ctcaaccaag tcattctgag aatagtgtat gcggcgaccg agttgctctt gcccggcgtc

4021 aacacgggat aataccgcgc cacatagcag aactttaaaa gtgctcatca ttggaaaacg

4081 ttcttcgggg cgaaaactct caaggatctt accgctgttg agatccagtt cgatgtaacc

4141 cactcgtgca cccaactgat cttcagcatc ttttactttc accagcgttt ctgggtgagc

4201 aaaaacagga aggcaaaatg ccgcaaaaaa gggaataagg gcgacacgga aatgttgaat

4261 actcatactc ttcctttttc aatattattg aagcatttat cagggttatt gtctcatgag

4321 cggatacata tttgaatgta tttagaaaaa taaacaaata ggggttccgc gcacatttcc

4381 ccgaaaagtg ccacctgacg tctaagaaac cattattatc atgacat

//

**CA97.86:**

LOCUS CA97.86 4394 bp DNA circular SYN 08-JUL-2021

DEFINITION synthetic circular DNA

ACCESSION .

VERSION .

KEYWORDS .

SOURCE synthetic DNA construct

ORGANISM synthetic DNA construct

REFERENCE 1 (bases 1 to 4394)

AUTHORS .

TITLE .

JOURNAL .

FEATURES Location/Qualifiers

source 1..4394

/organism="synthetic DNA construct"

/mol_type="other DNA"

promoter 113..442

/label=SV40 promoter

/note="SV40 enhancer and early promoter"

rep_origin 293..428

/label=SV40 ori

/note="SV40 origin of replication"

CDS 548..823

/codon_start=1

/label=GAL4 DBD ""Mut7""

/translation="MKLLSSIEQACDICRLKKLKCAQEFPSCKRCAKNNWECRYSPKTK RSPLTRAHLTEVESRLERLEQLFLLIFPREDLDMILKMDSLQDIKAL"

CDS 833..877

/codon_start=1

/label=3x GGGGS

/translation="GGGGSGGGGSGGGGS"

CDS 893..937

/codon_start=1

/label=3x GGGGS

/translation="GGGGSGGGGSGGGGS"

CDS 962..1771

/codon_start=1

/label=GR LBD CS1/CD 524

/translation="AGVSQDTSENPNKTIVPAALPQLTPTLVSLLEVIEPEVLYAGYDS SVPDSAWRIMTTLNMLGGRQVIAAVKWAKAIPGFRNLHLDDQMTLLQYSWMFLMAFALG WRSYRQSSGNLLCFAPDLIINEQRMSLPCMYDQCKHMLFVSSELQRLQVSYEEYLCMKT LLLLSSVPKEGLKSQELFDEIRMTYIKELGKAIVKREGNSSQNWQRFYQLTKLLDSMHE VVENLLTYCFQTFLDKTMSIEFPEAAAEIITNQIYSNGNIKKLLFHQK"

CDS 1776..1820

/codon_start=1

/label=3x GGGGS

/translation="GEVAVVEADQVEAVV"

CDS 1865..2098

/codon_start=1

/gene="UL48"

/product="transcriptional activation domain of herpes

simplex virus protein VP16 (Triezenberg et al., 1988;

Cousens et al., 1989)"

/label=VP16 AD

/translation="APPTDVSLGDELHLDGEDVAMAHADALDDFDLDMLGDGDSPGPGF TPHDSAPYGALDMADFEFEQMFTDALGIDEYGG"

polyA_signal 2115..2249

/label=SV40 poly(A) signal

/note="SV40 polyadenylation signal"

rep_origin complement(2614..3202)

/direction=LEFT

/label=ori

/note="high-copy-number ColE1/pMB1/pBR322/pUC origin of

replication"

CDS complement(3373..4233)

/codon_start=1

/gene="bla"

/product="beta-lactamase"

/label=AmpR

/note="confers resistance to ampicillin, carbenicillin, and related antibiotics"

/translation="MSIQHFRVALIPFFAAFCLPVFAHPETLVKVKDAEDQLGARVGYI ELDLNSGKILESFRPEERFPMMSTFKVLLCGAVLSRVDAGQEQLGRRIHYSQNDLVEYS PVTEKHLTDGMTVRELCSAAITMSDNTAANLLLTTIGGPKELTAFLHNMGDHVTRLDRW EPELNEAIPNDERDTTMPVAMATTLRKLLTGELLTLASRQQLIDWMEADKVAGPLLRSA LPAGWFIADKSGAGERGSRGIIAALGPDGKPSRIVVIYTTGSQATMDERNRQIAEIGAS LIKHW"

promoter complement(4234..4338)

/gene="bla"

/label=AmpR promoter

ORIGIN

1 taacctataa aaataggcgt atcacgaggc cctttcgtct tcaagaattg gtcgatcgac

61 caattctcat gtttgacagc ttatcatcga taagctagct tggctgtgga atgtgtgtca

121 gttagggtgt ggaaagtccc caggctcccc agcaggcaga agtatgcaaa gcatgcatct

181 caattagtca gcaaccaggt gtggaaagtc cccaggctcc ccagcaggca gaagtatgca

241 aagcatgcat ctcaattagt cagcaaccat agtcccgccc ctaactccgc ccatcccgcc

301 cctaactccg cccagttccg cccattctcc gccccatggc tgactaattt tttttattta

361 tgcagaggcc gaggccgcct cggcctctga gctattccag aagtagtgag gaggcttttt

421 tggaggccta ggcttttgca aaaagctcct cgaggaactg aaaaaccaga aagttaactg

481 gtaagtttag tctttttgtc ttttatttca ggtcccggat cggaattgcg cggccgcatc

541 cgggaccatg aagctactgt cttctatcga acaagcatgc gatatttgcc gacttaaaaa

601 gctcaagtgc gcccaagaat tcccgagctg caagaggtgt gcgaagaaca actgggagtg

661 tcgctactct cccaagacca aaaggtctcc gctgactagg gcacatctga cagaagtgga

721 atcaaggcta gaaagactgg aacagctatt tctactgatt tttcctcgag aagaccttga

781 catgattttg aaaatggatt ctttacagga tataaaagca ttgttgggtg ccggaggcgg

841 gggctcggga ggtggcggct ctggaggagg cggtagtacc gggtacaagg ccggaggcgg

901 gggctcggga ggtggcggct ctggaggagg cggtagtacc gggaccatgt tgggtacccc

961 tgcaggagtc tcacaagaca cttcggaaaa tcctaacaaa acaatagttc ctgcagcatt

1021 accacagctc acccctacct tggtgtcact gctggaggtg attgaacccg aggtgttgta

1081 tgcaggatat gatagctctg ttccagattc agcatggaga attatgacca cactcaacat

1141 gttaggtggg cgtcaagtga ttgcagcagt gaaatgggca aaggcgatac caggcttcag

1201 aaacttacac ctggatgacc aaatgaccct gctacagtac tcatggatgt ttctcatggc

1261 atttgccctg ggttggagat catacagaca atcaagtgga aacctgctct gctttgctcc

1321 tgatctgatt attaatgagc agagaatgtc tctaccctgc atgtatgacc aatgtaaaca

1381 catgctgttt gtctcctctg aattacaaag attgcaggta tcctatgaag agtatctctg

1441 tatgaaaacc ttactgcttc tctcctcagt tcctaaggaa ggtctgaaga gccaagagtt

1501 atttgatgag attcgaatga cttatatcaa agagctagga aaagccatcg tcaaaaggga

1561 agggaactcc agtcagaact ggcaacggtt ttaccaactg acaaagcttc tggactccat

1621 gcatgaggtg gttgagaatc tccttaccta ctgcttccag acatttttgg ataagaccat

1681 gagtattgaa ttcccagagg cggccgctga aatcatcact aatcagatat attcaaatgg

1741 aaatatcaaa aagcttctgt ttcatcagaa ggccggggga ggtggcagtg gtggaggcgg

1801 atcaggtgga ggcggtagtg ccggtacccc tgcagctgcg tcgacccaat tcccggggat

1861 ctgggccccc ccgaccgatg tcagcctggg ggacgagctc cacttagacg gcgaggacgt

1921 ggcgatggcg catgccgacg cgctagacga tttcgatctg gacatgttgg gggacgggga

1981 ttccccgggt ccgggattta ccccccacga ctccgccccc tacggcgctc tggatatggc

2041 cgacttcgag tttgagcaga tgtttaccga tgcccttgga attgacgagt acggtgggta

2101 gaccggcaag cttcgatcca gacatgataa gatacattga tgagtttgga caaaccacaa

2161 ctagaatgca gtgaaaaaaa tgctttattt gtgaaatttg tgatgctatt gctttatttg

2221 taaccattat aagctgcaat aaacaagtta acaacaacaa ttgcattcat tttatgtttc

2281 aggttcaggg ggaggtgtgg gaggtttttt aaagcaagta aaacctctac aaatgtggta

2341 tggctgatta tgatccggct gcctcgcgcg tttcggtgat gacggtgaaa acctctgaca

2401 catgcagctc ccggagacgg tcacagcttg tctgtaagcg gatgccggga gcagacaagc

2461 ccgtcagggc gcgtcagcgg gtgttggcgg gtgtcggggc gcagccatga cccagtcacg

2521 tagcgatagc ggagtgtata ctggcttaac tatgcggcat cagagcagat tgtactgaga

2581 gtgcaccata tgtcgggccg cgttgctggc gtttttccat aggctccgcc cccctgacga

2641 gcatcacaaa aatcgacgct caagtcagag gtggcgaaac ccgacaggac tataaagata

2701 ccaggcgttt ccccctggaa gctccctcgt gcgctctcct gttccgaccc tgccgcttac

2761 cggatacctg tccgcctttc tcccttcggg aagcgtggcg ctttctcata gctcacgctg

2821 taggtatctc agttcggtgt aggtcgttcg ctccaagctg ggctgtgtgc acgaaccccc

2881 cgttcagccc gaccgctgcg ccttatccgg taactatcgt cttgagtcca acccggtaag

2941 acacgactta tcgccactgg cagcagccac tggtaacagg attagcagag cgaggtatgt

3001 aggcggtgct acagagttct tgaagtggtg gcctaactac ggctacacta gaaggacagt

3061 atttggtatc tgcgctctgc tgaagccagt taccttcgga aaaagagttg gtagctcttg

3121 atccggcaaa caaaccaccg ctggtagcgg tggttttttt gtttgcaagc agcagattac

3181 gcgcagaaaa aaaggatctc aagaagatcc tttgatcttt tctacggggt ctgacgctca

3241 gtggaacgaa aactcacgtt aagggatttt ggtcatgaga ttatcaaaaa ggatcttcac

3301 ctagatcctt ttaaattaaa aatgaagttt taaatcaatc taaagtatat atgagtaaac

3361 ttggtctgac agttaccaat gcttaatcag tgaggcacct atctcagcga tctgtctatt

3421 tcgttcatcc atagttgcct gactccccgt cgtgtagata actacgatac gggagggctt

3481 accatctggc cccagtgctg caatgatacc gcgagaccca cgctcaccgg ctccagattt

3541 atcagcaata aaccagccag ccggaagggc cgagcgcaga agtggtcctg caactttatc

3601 cgcctccatc cagtctatta attgttgccg ggaagctaga gtaagtagtt cgccagttaa

3661 tagtttgcgc aacgttgttg ccattgctac aggcatcgtg gtgtcacgct cgtcgtttgg

3721 tatggcttca ttcagctccg gttcccaacg atcaaggcga gttacatgat cccccatgtt

3781 gtgcaaaaaa gcggttagct ccttcggtcc tccgatcgtt gtcagaagta agttggccgc

3841 agtgttatca ctcatggtta tggcagcact gcataattct cttactgtca tgccatccgt

3901 aagatgcttt tctgtgactg gtgagtactc aaccaagtca ttctgagaat agtgtatgcg

3961 gcgaccgagt tgctcttgcc cggcgtcaac acgggataat accgcgccac atagcagaac

4021 tttaaaagtg ctcatcattg gaaaacgttc ttcggggcga aaactctcaa ggatcttacc

4081 gctgttgaga tccagttcga tgtaacccac tcgtgcaccc aactgatctt cagcatcttt

4141 tactttcacc agcgtttctg ggtgagcaaa aacaggaagg caaaatgccg caaaaaaggg

4201 aataagggcg acacggaaat gttgaatact catactcttc ctttttcaat attattgaag

4261 catttatcag ggttattgtc tcatgagcgg atacatattt gaatgtattt agaaaaataa

4321 acaaataggg gttccgcgca catttccccg aaaagtgcca cctgacgtct aagaaaccat

4381 tattatcatg acat

//

**CA97.87:**

LOCUS CA97.87 4455 bp DNA circular SYN 08-JUL-2021

DEFINITION synthetic circular DNA

ACCESSION .

VERSION .

KEYWORDS .

SOURCE synthetic DNA construct

ORGANISM synthetic DNA construct

REFERENCE 1 (bases 1 to 4455)

AUTHORS .

TITLE .

JOURNAL .

FEATURES Location/Qualifiers

source 1..4455

/organism="synthetic DNA construct"

/mol_type="other DNA"

promoter 113..442

/label=SV40 promoter

/note="SV40 enhancer and early promoter"

rep_origin 293..428

/label=SV40 ori

/note="SV40 origin of replication"

CDS 548..823

/codon_start=1

/label=GAL4 DBD ""Mut7""

/translation="MKLLSSIEQACDICRLKKLKCAQEFPSCKRCAKNNWECRYSPKTK RSPLTRAHLTEVESRLERLEQLFLLIFPREDLDMILKMDSLQDIKAL"

CDS 833..877

/codon_start=1

/label=3x GGGGS

/translation="GGGGSGGGGSGGGGS"

CDS 893..937

/codon_start=1

/label=3x GGGGS

/translation="GGGGSGGGGSGGGGS"

CDS 962..1834

/codon_start=1

/label=GR LBD CS1/CD 504

/translation="AGMNLEARKTKKKIKGIQQATAGVSQDTSENPNKTIVPAALPQLT PTLVSLLEVIEPEVLYAGYDSSVPDSAWRIMTTLNMLGGRQVIAAVKWAKAIPGFRNLH LDDQMTLLQYSWMFLMAFALGWRSYRQSSGNLLCFAPDLIINEQRMSLPCMYDQCKHML FVSSELQRLQVSYEEYLCMKTLLLLSSVPKEGLKSQELFDEIRMTYIKELGKAIVKREG NSSQNWQRFYQLTKLLDSMHEVVENLLTYCFQTFLDKTMSIEFPEAAAEIITNQIYSNG NIKKLLFHQK"

CDS 1839..1883

/codon_start=1

/label=3x GGGGS

/translation="GEVAVVEADQVEAVV"

CDS 1928..2161

/codon_start=1

/gene="UL48"

/product="transcriptional activation domain of herpes

simplex virus protein VP16 (Triezenberg et al., 1988;

Cousens et al., 1989)"

/label=VP16 AD

/translation="APPTDVSLGDELHLDGEDVAMAHADALDDFDLDMLGDGDSPGPGF TPHDSAPYGALDMADFEFEQMFTDALGIDEYGG"

polyA_signal 2178..2312

/label=SV40 poly(A) signal

/note="SV40 polyadenylation signal"

rep_origin complement(2675..3263)

/direction=LEFT

/label=ori

/note="high-copy-number ColE1/pMB1/pBR322/pUC origin of

replication"

CDS complement(3434..4294)

/codon_start=1

/gene="bla"

/product="beta-lactamase"

/label=AmpR

/note="confers resistance to ampicillin, carbenicillin, and related antibiotics"

/translation="MSIQHFRVALIPFFAAFCLPVFAHPETLVKVKDAEDQLGARVGYI ELDLNSGKILESFRPEERFPMMSTFKVLLCGAVLSRVDAGQEQLGRRIHYSQNDLVEYS PVTEKHLTDGMTVRELCSAAITMSDNTAANLLLTTIGGPKELTAFLHNMGDHVTRLDRW EPELNEAIPNDERDTTMPVAMATTLRKLLTGELLTLASRQQLIDWMEADKVAGPLLRSA LPAGWFIADKSGAGERGSRGIIAALGPDGKPSRIVVIYTTGSQATMDERNRQIAEIGAS LIKHW"

promoter complement(4295..4399)

/gene="bla"

/label=AmpR promoter

ORIGIN

1 taacctataa aaataggcgt atcacgaggc cctttcgtct tcaagaattg gtcgatcgac

61 caattctcat gtttgacagc ttatcatcga taagctagct tggctgtgga atgtgtgtca

121 gttagggtgt ggaaagtccc caggctcccc agcaggcaga agtatgcaaa gcatgcatct

181 caattagtca gcaaccaggt gtggaaagtc cccaggctcc ccagcaggca gaagtatgca

241 aagcatgcat ctcaattagt cagcaaccat agtcccgccc ctaactccgc ccatcccgcc

301 cctaactccg cccagttccg cccattctcc gccccatggc tgactaattt tttttattta

361 tgcagaggcc gaggccgcct cggcctctga gctattccag aagtagtgag gaggcttttt

421 tggaggccta ggcttttgca aaaagctcct cgaggaactg aaaaaccaga aagttaactg

481 gtaagtttag tctttttgtc ttttatttca ggtcccggat cggaattgcg cggccgcatc

541 cgggaccatg aagctactgt cttctatcga acaagcatgc gatatttgcc gacttaaaaa

601 gctcaagtgc gcccaagaat tcccgagctg caagaggtgt gcgaagaaca actgggagtg

661 tcgctactct cccaagacca aaaggtctcc gctgactagg gcacatctga cagaagtgga

721 atcaaggcta gaaagactgg aacagctatt tctactgatt tttcctcgag aagaccttga

781 catgattttg aaaatggatt ctttacagga tataaaagca ttgttgggtg ccggaggcgg

841 gggctcggga ggtggcggct ctggaggagg cggtagtacc gggtacaagg ccggaggcgg

901 gggctcggga ggtggcggct ctggaggagg cggtagtacc gggaccatgt tgggtacccc

961 tgcaggaatg aaccttgaag ctcgaaaaac aaagaaaaaa atcaaaggga ttcagcaagc

1021 cactgcagga gtctcacaag acacttcgga aaatcctaac aaaacaatag ttcctgcagc

1081 attaccacag ctcaccccta ccttggtgtc actgctggag gtgattgaac ccgaggtgtt

1141 gtatgcagga tatgatagct ctgttccaga ttcagcatgg agaattatga ccacactcaa

1201 catgttaggt gggcgtcaag tgattgcagc agtgaaatgg gcaaaggcga taccaggctt

1261 cagaaactta cacctggatg accaaatgac cctgctacag tactcatgga tgtttctcat

1321 ggcatttgcc ctgggttgga gatcatacag acaatcaagt ggaaacctgc tctgctttgc

1381 tcctgatctg attattaatg agcagagaat gtctctaccc tgcatgtatg accaatgtaa

1441 acacatgctg tttgtctcct ctgaattaca aagattgcag gtatcctatg aagagtatct

1501 ctgtatgaaa accttactgc ttctctcctc agttcctaag gaaggtctga agagccaaga

1561 gttatttgat gagattcgaa tgacttatat caaagagcta ggaaaagcca tcgtcaaaag

1621 ggaagggaac tccagtcaga actggcaacg gttttaccaa ctgacaaagc ttctggactc

1681 catgcatgag gtggttgaga atctccttac ctactgcttc cagacatttt tggataagac

1741 catgagtatt gaattcccag aggcggccgc tgaaatcatc actaatcaga tatattcaaa

1801 tggaaatatc aaaaagcttc tgtttcatca gaaggccggg ggaggtggca gtggtggagg

1861 cggatcaggt ggaggcggta gtgccggtac ccctgcagct gcgtcgaccc aattcccggg

1921 gatctgggcc cccccgaccg atgtcagcct gggggacgag ctccacttag acggcgagga

1981 cgtggcgatg gcgcatgccg acgcgctaga cgatttcgat ctggacatgt tgggggacgg

2041 ggattccccg ggtccgggat ttacccccca cgactccgcc ccctacggcg ctctggatat

2101 ggccgacttc gagtttgagc agatgtttac cgatgccctt ggaattgacg agtacggtgg

2161 gtagaccggc aagcttcgat ccagacatga taagatacat tgatgagttt ggacaaacca

2221 caactagaat gcagtgaaaa aaatgcttta tttgtgaaat ttgtgatgct attgctttat

2281 ttgtaaccat tataagctgc aataaacaag ttaacaacaa caattgcatt cattttatgt

2341 ttcaggttca gggggaggtg tgggaggttt tttaaagcaa gtaaaacctc tacaaatgtg

2401 gtatggctga ttatgatccg gctgcctcgc gcgtttcggt gatgacggtg aaaacctctg

2461 acacatgcag ctcccggaga cggtcacagc ttgtctgtaa gcggatgccg ggagcagaca

2521 agcccgtcag ggcgcgtcag cgggtgttgg cgggtgtcgg ggcgcagcca tgacccagtc

2581 acgtagcgat agcggagtgt atactggctt aactatgcgg catcagagca gattgtactg

2641 agagtgcacc atatcgggcc gcgttgctgg cgtttttcca taggctccgc ccccctgacg

2701 agcatcacaa aaatcgacgc tcaagtcaga ggtggcgaaa cccgacagga ctataaagat

2761 accaggcgtt tccccctgga agctccctcg tgcgctctcc tgttccgacc ctgccgctta

2821 ccggatacct gtccgccttt ctcccttcgg gaagcgtggc gctttctcat agctcacgct

2881 gtaggtatct cagttcggtg taggtcgttc gctccaagct gggctgtgtg cacgaacccc

2941 ccgttcagcc cgaccgctgc gccttatccg gtaactatcg tcttgagtcc aacccggtaa

3001 gacacgactt atcgccactg gcagcagcca ctggtaacag gattagcaga gcgaggtatg

3061 taggcggtgc tacagagttc ttgaagtggt ggcctaacta cggctacact agaaggacag

3121 tatttggtat ctgcgctctg ctgaagccag ttaccttcgg aaaaagagtt ggtagctctt

3181 gatccggcaa acaaaccacc gctggtagcg gtggtttttt tgtttgcaag cagcagatta

3241 cgcgcagaaa aaaaggatct caagaagatc ctttgatctt ttctacgggg tctgacgctc

3301 agtggaacga aaactcacgt taagggattt tggtcatgag attatcaaaa aggatcttca

3361 cctagatcct tttaaattaa aaatgaagtt ttaaatcaat ctaaagtata tatgagtaaa

3421 cttggtctga cagttaccaa tgcttaatca gtgaggcacc tatctcagcg atctgtctat

3481 ttcgttcatc catagttgcc tgactccccg tcgtgtagat aactacgata cgggagggct

3541 taccatctgg ccccagtgct gcaatgatac cgcgagaccc acgctcaccg gctccagatt

3601 tatcagcaat aaaccagcca gccggaaggg ccgagcgcag aagtggtcct gcaactttat

3661 ccgcctccat ccagtctatt aattgttgcc gggaagctag agtaagtagt tcgccagtta

3721 atagtttgcg caacgttgtt gccattgcta caggcatcgt ggtgtcacgc tcgtcgtttg

3781 gtatggcttc attcagctcc ggttcccaac gatcaaggcg agttacatga tcccccatgt

3841 tgtgcaaaaa agcggttagc tccttcggtc ctccgatcgt tgtcagaagt aagttggccg

3901 cagtgttatc actcatggtt atggcagcac tgcataattc tcttactgtc atgccatccg

3961 taagatgctt ttctgtgact ggtgagtact caaccaagtc attctgagaa tagtgtatgc

4021 ggcgaccgag ttgctcttgc ccggcgtcaa cacgggataa taccgcgcca catagcagaa

4081 ctttaaaagt gctcatcatt ggaaaacgtt cttcggggcg aaaactctca aggatcttac

4141 cgctgttgag atccagttcg atgtaaccca ctcgtgcacc caactgatct tcagcatctt

4201 ttactttcac cagcgtttct gggtgagcaa aaacaggaag gcaaaatgcc gcaaaaaagg

4261 gaataagggc gacacggaaa tgttgaatac tcatactctt cctttttcaa tattattgaa

4321 gcatttatca gggttattgt ctcatgagcg gatacatatt tgaatgtatt tagaaaaata

4381 aacaaatagg ggttccgcgc acatttcccc gaaaagtgcc acctgacgtc taagaaacca

4441 ttattatcat gacat

//

**E000.29:**

LOCUS E000.29 3428 bp DNA circular SYN 06-JUL-2021

DEFINITION synthetic circular DNA

ACCESSION .

VERSION .

KEYWORDS .

SOURCE synthetic DNA construct

ORGANISM synthetic DNA construct

REFERENCE 1 (bases 1 to 3428)

AUTHORS .

TITLE .

JOURNAL .

FEATURES Location/Qualifiers

source 1..3428

/organism="synthetic DNA construct"

/mol_type="other DNA"

polyA_signal 2..50

/label=Synth. poly(A) signal

/note="synthetic polyadenylation signal"

misc_feature 64..155

/label=pause site

/note="RNA polymerase II transcriptional pause signal from the human alpha-2 globin gene"

protein_bind 245..339

/label=5X UAS

/bound_moiety="GAL4"

/note="five tandem copies of the ""ScaI site"" 17-mer

CGGAGTACTGTCCTCCG, an upstream activating sequence(UAS), that efficiently binds yeast Gal4 (Webster et al., 1988; Pfeiffer et al., 2010)"

promoter 340..454

/label=Minimal promoter

regulatory 549..558

/regulatory_class="other"

/label=Kozak sequence

/note="vertebrate consensus sequence for strong initiation of translation (Kozak, 1987)"

CDS 555..1112

/codon_start=1

/product="secreted Gaussia luciferase"

/label=hGLuc

/note="human codon-optimized"

/translation="MGVKVLFALICIAVAEAKPTENNEDFNIVAVASNFATTDLDADRG KLPGKKLPLEVLKEMEANARKAGCTRGCLICLSHIKCTPKMKKFIPGRCHTYEGDKESA QGGIGEAIVDIPEIPGFKDLEPMEQFIAQVDLCVDCTTGCLKGLANVQCSDLLKKWLPQ RCATFASKIQGQVDKIKGAGGD"

polyA_signal 1147..1268

/label=SV40 poly(A) signal

/note="SV40 polyadenylation signal"

rep_origin complement(1677..2265)

/direction=LEFT

/label=ori

/note="high-copy-number ColE1/pMB1/pBR322/pUC origin of

replication"

CDS complement(2465..3325)

/codon_start=1

/product="beta-lactamase"

/label=AmpR

/note="confers resistance to ampicillin, carbenicillin, and related antibiotics"

/translation="MSIQHFRVALIPFFAAFCLPVFAHPETLVKVKDAEDQLGARVGYI ELDLNSGKILESFRPEERFPMMSTFKVLLCGAVLSRIDAGQEQLGRRIHYSQNDLVEYS PVTEKHLTDGMTVRELCSAAITMSDNTAANLLLTTIGGPKELTAFLHNMGDHVTRLDRW EPELNEAIPNDERDTTMPVAMATTLRKLLTGELLTLASRQQLIDWMEADKVAGPLLRSA LPAGWFIADKSGAGERGSRGIIAALGPDGKPSRIVVIYTTGSQATMDERNRQIAEIGAS LIKHW"

ORIGIN

1 caataaaata tctttatttt cattacatct gtgtgttggt tttttgtgtg aatcgatagt

61 actaacatac gctctccatc aaaacaaaac gaaacaaaac aaactagcaa aataggctgt

121 ccccagtgca agtgcaggtg ccagaacatt tctctggcct aactggccgg tacctgagct

181 cgctagcctc gaggataatt cctcgacgga tctgcgatct aagtaagctt gcatgcctgc

241 aggtcggagt actgtcctcc gagcggagta ctgtcctccg agcggagtac tgtcctccga

301 gcggagtact gtcctccgag cggagtactg tcctccgagc ggagactcta gcgagataca

361 tgtcgtcgac cttgggcata aaaggcagag cactgcagct gctgcttaca cttgcttttg

421 acacaactgt gtttacttgc aatcccccaa gcttcacata tgcatgcact agtggcgcct

481 gtcgacgcgt agaattatca agatctggcc tcggcggcca agcttggcaa tccggtactg

541 ttggtaaagc caccatggga gtcaaagttc tgtttgccct gatctgcatc gctgtggccg

601 aggccaagcc caccgagaac aacgaagact tcaacatcgt ggccgtggcc agcaacttcg

661 cgaccacgga tctcgatgct gaccgcggga agttgcccgg caagaagctg ccgctggagg

721 tgctcaaaga gatggaagcc aatgcccgga aagctggctg caccaggggc tgtctgatct

781 gcctgtccca catcaagtgc acgcccaaga tgaagaagtt catcccagga cgctgccaca

841 cctacgaagg cgacaaagag tccgcacagg gcggcatagg cgaggcgatc gtcgacattc

901 ctgagattcc tgggttcaag gacttggagc ctatggagca gttcatcgca caggtcgatc

961 tgtgtgtgga ctgcacaact ggctgcctca aagggcttgc caacgtgcag tgttctgacc

1021 tgctcaagaa gtggctgccg caacgctgtg cgacctttgc cagcaagatc cagggccagg

1081 tggacaagat caagggggcc ggtggtgact aagcggccgc tcgagcatgc atctagacag

1141 acatgataag atacattgat gagtttggac aaaccacaac tagaatgcag tgaaaaaaat

1201 gctttatttg tgaaatttgt gatgctattg ctttatttgt aaccattata agctgcaata

1261 aacaagttaa caacaacaat tgcattcatt ttatgtttca ggttcagggg gaggtgtggg

1321 aggtttttta aagcaagtaa aacctctaca aatgtggtag gatccgtcga ccgatgccct

1381 tgagagcctt caacccagtc agctccttcc ggtgggcgcg gggcatgact atcgtcgccg

1441 cacttatgac tgtcttcttt atcatgcaac tcgtaggaca ggtgccggca gcgctcttcc

1501 gcttcctcgc tcactgactc gctgcgctcg gtcgttcggc tgcggcgagc ggtatcagct

1561 cactcaaagg cggtaatacg gttatccaca gaatcagggg ataacgcagg aaagaacatg

1621 tgagcaaaag gccagcaaaa ggccaggaac cgtaaaaagg ccgcgttgct ggcgtttttc

1681 cataggctcc gcccccctga cgagcatcac aaaaatcgac gctcaagtca gaggtggcga

1741 aacccgacag gactataaag ataccaggcg tttccccctg gaagctccct cgtgcgctct

1801 cctgttccga ccctgccgct taccggatac ctgtccgcct ttctcccttc gggaagcgtg

1861 gcgctttctc atagctcacg ctgtaggtat ctcagttcgg tgtaggtcgt tcgctccaag

1921 ctgggctgtg tgcacgaacc ccccgttcag cccgaccgct gcgccttatc cggtaactat

1981 cgtcttgagt ccaacccggt aagacacgac ttatcgccac tggcagcagc cactggtaac

2041 aggattagca gagcgaggta tgtaggcggt gctacagagt tcttgaagtg gtggcctaac

2101 tacggctaca ctagaagaac agtatttggt atctgcgctc tgctgaagcc agttaccttc

2161 ggaaaaagag ttggtagctc ttgatccggc aaacaaacca ccgctggtag cggtggtttt

2221 tttgtttgca agcagcagat tacgcgcaga aaaaaaggat ctcaagaaga tcctttgatc

2281 ttttctacgg ggtctgacgc tcagtggaac gaaaactcac gttaagggat tttggtcatg

2341 agattatcaa aaaggatctt cacctagatc cttttaaatt aaaaatgaag ttttaaatca

2401 atctaaagta tatatgagta aacttggtct gacagcggcc gcaaatgcta aaccactgca

2461 gtggttacca gtgcttgatc agtgaggcac cgatctcagc gatctgccta tttcgttcgt

2521 ccatagtggc ctgactcccc gtcgtgtaga tcactacgat tcgtgagggc ttaccatcag

2581 gccccagcgc agcaatgatg ccgcgagagc cgcgttcacc ggcccccgat ttgtcagcaa

2641 tgaaccagcc agcagggagg gccgagcgaa gaagtggtcc tgctactttg tccgcctcca

2701 tccagtctat gagctgctgt cgtgatgcta gagtaagaag ttcgccagtg agtagtttcc

2761 gaagagttgt ggccattgct actggcatcg tggtatcacg ctcgtcgttc ggtatggctt

2821 cgttcaactc tggttcccag cggtcaagcc gggtcacatg atcacccata ttatgaagaa

2881 atgcagtcag ctccttaggg cctccgatcg ttgtcagaag taagttggcc gcggtgttgt

2941 cgctcatggt aatggcagca ctacacaatt ctcttaccgt catgccatcc gtaagatgct

3001 tttccgtgac cggcgagtac tcaaccaagt cgttttgtga gtagtgtata cggcgaccaa

3061 gctgctcttg cccggcgtct atacgggaca acaccgcgcc acatagcagt actttgaaag

3121 tgctcatcat cgggaatcgt tcttcggggc ggaaagactc aaggatcttg ccgctattga

3181 gatccagttc gatatagccc actcttgcac ccagttgatc ttcagcatct tttactttca

3241 ccagcgtttc ggggtgtgca aaaacaggca agcaaaatgc cgcaaagaag ggaatgagtg

3301 cgacacgaaa atgttggatg ctcatactcg tcctttttca atattattga agcatttatc

3361 agggttacta gtacgtctct caaggataag taagtaatat taaggtacgg gaggtattgg

3421 acaggccg

//

**ST00.1:**

LOCUS ST00.1 5125 bp DNA circular SYN 09-JUL-2021

DEFINITION synthetic circular DNA

ACCESSION .

VERSION .

KEYWORDS .

SOURCE synthetic DNA construct

ORGANISM synthetic DNA construct

REFERENCE 1 (bases 1 to 5125)

AUTHORS .

TITLE .

JOURNAL .

FEATURES Location/Qualifiers

source 1..5125

/organism="synthetic DNA construct"

/mol_type="other DNA"

promoter 48..244

/label=SV40 promoter

/note="SV40 early promoter"

rep_origin 95..230

/label=SV40 ori

/note="SV40 origin of replication"

CDS 282..1784

/codon_start=1

/product="secreted alkaline phosphatase from human

placenta"

/label=SEAP

/note="The natural enzyme has a C-terminal transmembrane sequence that is replaced by a GPI anchor."

/translation="MLLLLLLLGLRLQLSLGIIPVEEENPDFWNREAAEALGAAKKLQP AQTAAKNLIIFLGDGMGVSTVTAARILKGQKKDKLGPEIPLAMDRFPYVALSKTYNVDK HVPDSGATATAYLCGVKGNFQTIGLSAAARFNQCNTTRGNEVISVMNRAKKAGKSVGVV TTTRVQHASPAGTYAHTVNRNWYSDADVPASARQEGCQDIATQLISNMDIDVILGGGRK YMFRMGTPDPEYPDDYSQGGTRLDGKNLVQEWLAKRQGARYVWNRTELMQASLDPSVTH LMGLFEPGDMKYEIHRDSTLDPSLMEMTEAALRLLSRNPRGFFLFVEGGRIDHGHHESR AYRALTETIMFDDAIERAGQLTSEEDTLSLVTADHSHVFSFGGYPLRGSSIFGLAPGKA RDRKAYTVLLYGNGPGYVLKDGARPDVTESESGSPEYRQQSAVPLDEETHAGEDVAVFA RGPQAHLVHGVQEQTFIAHVMAFAACLEPYTACDLAPPAGTTD"

polyA_signal 1842..1963

/label=SV40 poly(A) signal

/note="SV40 polyadenylation signal"

rep_origin complement(2628..3216)

/direction=LEFT

/label=ori

/note="high-copy-number ColE1/pMB1/pBR322/pUC origin of

replication"

CDS complement(3387..4247)

/codon_start=1

/gene="bla"

/product="beta-lactamase"

/label=AmpR

/note="confers resistance to ampicillin, carbenicillin, and related antibiotics"

/translation="MSIQHFRVALIPFFAAFCLPVFAHPETLVKVKDAEDQLGARVGYI ELDLNSGKILESFRPEERFPMMSTFKVLLCGAVLSRIDAGQEQLGRRIHYSQNDLVEYS PVTEKHLTDGMTVRELCSAAITMSDNTAANLLLTTIGGPKELTAFLHNMGDHVTRLDRW EPELNEAIPNDERDTTMPVAMATTLRKLLTGELLTLASRQQLIDWMEADKVAGPLLRSA LPAGWFIADKSGAGERGSRGIIAALGPDGKPSRIVVIYTTGSQATMDERNRQIAEIGAS LIKHW"

promoter complement(4248..4352)

/gene="bla"

/label=AmpR promoter

rep_origin 4379..4834

/direction=RIGHT

/label=f1 ori

/note="f1 bacteriophage origin of replication; arrow

indicates direction of (+) strand synthesis"

polyA_signal 4965..5013

/note="synthetic polyadenylation signal"

misc_feature 5027..5118

/label=pause site

/note="RNA polymerase II transcriptional pause signal from the human alpha-2 globin gene"

ORIGIN

1 ggtaccgagc tcttacgcgt gctagcccgg gctcgagatc tgcgatctgc atctcaatta

61 gtcagcaacc atagtcccgc ccctaactcc gcccatcccg cccctaactc cgcccagttc

121 cgcccattct ccgccccatc gctgactaat tttttttatt tatgcagagg ccgaggccgc

181 ctcggcctct gagctattcc agaagtagtg aggaggcttt tttggaggcc taggcttttg

241 caaaaagctt cgaagtttat cctgcaggga attcgcccac catgctgctg ctgctgctgc

301 tgctgggcct gaggctacag ctctccctgg gcatcatccc agttgaggag gagaacccgg

361 acttctggaa ccgcgaggca gccgaggccc tgggtgccgc caagaagctg cagcctgcac

421 agacagccgc caagaacctc atcatcttcc tgggcgatgg gatgggggtg tctacggtga

481 cagctgccag gatcctaaaa gggcagaaga aggacaaact ggggcctgag atacccctgg

541 ccatggaccg cttcccatat gtggctctgt ccaagacata caatgtagac aaacatgtgc

601 cagacagtgg agccacagcc acggcctacc tgtgcggggt caagggcaac ttccagacca

661 ttggcttgag tgcagccgcc cgctttaacc agtgcaacac gacacgcggc aacgaggtca

721 tctccgtgat gaatcgggcc aagaaagcag ggaagtcagt gggagtggta accaccacac

781 gagtgcagca cgcctcgcca gccggcacct acgcccacac ggtgaaccgc aactggtact

841 cggacgccga cgtgcctgcc tcggcccgcc aggaggggtg ccaggacatc gctacgcagc

901 tcatctccaa catggacatt gacgtgatcc taggtggagg ccgaaagtac atgtttcgca

961 tgggaacccc agaccctgag tacccagatg actacagcca aggtgggacc aggctggacg

1021 ggaagaatct ggtgcaggaa tggctggcga agcgccaggg tgcccggtat gtgtggaacc

1081 gcactgagct catgcaggct tccctggacc cgtctgtgac ccatctcatg ggtctctttg

1141 agcctggaga catgaaatac gagatccacc gagactccac actggacccc tccctgatgg

1201 agatgacaga ggctgccctg cgcctgctga gcaggaaccc ccgcggcttc ttcctcttcg

1261 tggagggtgg tcgcatcgac catggtcatc atgaaagcag ggcttaccgg gcactgactg

1321 agacgatcat gttcgacgac gccattgaga gggcgggcca gctcaccagc gaggaggaca

1381 cgctgagcct cgtcactgcc gaccactccc acgtcttctc cttcggaggc taccccctgc

1441 gagggagctc catcttcggg ctggcccctg gcaaggcccg ggacaggaag gcctacacgg

1501 tcctcctata cggaaacggt ccaggctatg tgctcaagga cggcgcccgg ccggatgtta

1561 ccgagagcga gagcgggagc cccgagtatc ggcagcagtc agcagtgccc ctggacgaag

1621 agacccacgc aggcgaggac gtggcggtgt tcgcgcgcgg cccgcaggcg cacctggttc

1681 acggcgtgca ggagcagacc ttcatagcgc acgtcatggc cttcgccgcc tgcctggagc

1741 cctacaccgc ctgcgacctg gcgccccccg ccggcaccac cgacgccgcg cacccgggtt

1801 actctagagt cggggcggcc ggccgcttcg agcagacatg ataagataca ttgatgagtt

1861 tggacaaacc acaactagaa tgcagtgaaa aaaatgcttt atttgtgaaa tttgtgatgc

1921 tattgcttta tttgtaacca ttataagctg caataaacaa gttaacaaca acaattgcat

1981 tcattttatg tttcaggttc agggggaggt gtgggaggtt ttttaaagca agtaaaacct

2041 ctacaaatgt ggtaaaatcg ataaggatct gaacgatgga gcggagaatg ggcggaactg

2101 ggcggagtta ggggcgggat gggcggagtt aggggcggga ctatggttgc tgactaattg

2161 agatgcatgc tttgcatact tctgcctgct ggggagcctg gggactttcc acacctggtt

2221 gctgactaat tgagatgcat gctttgcata cttctgcctg ctggggagcc tggggacttt

2281 ccacacccta actgacacac attccacagc ggatccgtcg accgatgccc ttgagagcct

2341 tcaacccagt cagctccttc cggtgggcgc ggggcatgac tatcgtcgcc gcacttatga

2401 ctgtcttctt tatcatgcaa ctcgtaggac aggtgccggc agcgctcttc cgcttcctcg

2461 ctcactgact cgctgcgctc ggtcgttcgg ctgcggcgag cggtatcagc tcactcaaag

2521 gcggtaatac ggttatccac agaatcaggg gataacgcag gaaagaacat gtgagcaaaa

2581 ggccagcaaa aggccaggaa ccgtaaaaag gccgcgttgc tggcgttttt ccataggctc

2641 cgcccccctg acgagcatca caaaaatcga cgctcaagtc agaggtggcg aaacccgaca

2701 ggactataaa gataccaggc gtttccccct ggaagctccc tcgtgcgctc tcctgttccg

2761 accctgccgc ttaccggata cctgtccgcc tttctccctt cgggaagcgt ggcgctttct

2821 catagctcac gctgtaggta tctcagttcg gtgtaggtcg ttcgctccaa gctgggctgt

2881 gtgcacgaac cccccgttca gcccgaccgc tgcgccttat ccggtaacta tcgtcttgag

2941 tccaacccgg taagacacga cttatcgcca ctggcagcag ccactggtaa caggattagc

3001 agagcgaggt atgtaggcgg tgctacagag ttcttgaagt ggtggcctaa ctacggctac

3061 actagaagga cagtatttgg tatctgcgct ctgctgaagc cagttacctt cggaaaaaga

3121 gttggtagct cttgatccgg caaacaaacc accgctggta gcggtggttt ttttgtttgc

3181 aagcagcaga ttacgcgcag aaaaaaagga tctcaagaag atcctttgat cttttctacg

3241 gggtctgacg ctcagtggaa cgaaaactca cgttaaggga ttttggtcat gagattatca

3301 aaaaggatct tcacctagat ccttttaaat taaaaatgaa gttttaaatc aatctaaagt

3361 atatatgagt aaacttggtc tgacagttac caatgcttaa tcagtgaggc acctatctca

3421 gcgatctgtc tatttcgttc atccatagtt gcctgactcc ccgtcgtgta gataactacg

3481 atacgggagg gcttaccatc tggccccagt gctgcaatga taccgcgaga cccacgctca

3541 ccggctccag atttatcagc aataaaccag ccagccggaa gggccgagcg cagaagtggt

3601 cctgcaactt tatccgcctc catccagtct attaattgtt gccgggaagc tagagtaagt

3661 agttcgccag ttaatagttt gcgcaacgtt gttgccattg ctacaggcat cgtggtgtca

3721 cgctcgtcgt ttggtatggc ttcattcagc tccggttccc aacgatcaag gcgagttaca

3781 tgatccccca tgttgtgcaa aaaagcggtt agctccttcg gtcctccgat cgttgtcaga

3841 agtaagttgg ccgcagtgtt atcactcatg gttatggcag cactgcataa ttctcttact

3901 gtcatgccat ccgtaagatg cttttctgtg actggtgagt actcaaccaa gtcattctga

3961 gaatagtgta tgcggcgacc gagttgctct tgcccggcgt caatacggga taataccgcg

4021 ccacatagca gaactttaaa agtgctcatc attggaaaac gttcttcggg gcgaaaactc

4081 tcaaggatct taccgctgtt gagatccagt tcgatgtaac ccactcgtgc acccaactga

4141 tcttcagcat cttttacttt caccagcgtt tctgggtgag caaaaacagg aaggcaaaat

4201 gccgcaaaaa agggaataag ggcgacacgg aaatgttgaa tactcatact cttccttttt

4261 caatattatt gaagcattta tcagggttat tgtctcatga gcggatacat atttgaatgt

4321 atttagaaaa ataaacaaat aggggttccg cgcacatttc cccgaaaagt gccacctgac

4381 gcgccctgta gcggcgcatt aagcgcggcg ggtgtggtgg ttacgcgcag cgtgaccgct

4441 acacttgcca gcgccctagc gcccgctcct ttcgctttct tcccttcctt tctcgccacg

4501 ttcgccggct ttccccgtca agctctaaat cgggggctcc ctttagggtt ccgatttagt

4561 gctttacggc acctcgaccc caaaaaactt gattagggtg atggttcacg tagtgggcca

4621 tcgccctgat agacggtttt tcgccctttg acgttggagt ccacgttctt taatagtgga

4681 ctcttgttcc aaactggaac aacactcaac cctatctcgg tctattcttt tgatttataa

4741 gggattttgc cgatttcggc ctattggtta aaaaatgagc tgatttaaca aaaatttaac

4801 gcgaatttta acaaaatatt aacgtttaca atttcccatt cgccattcag gctgcgcaac

4861 tgttgggaag ggcgatcggt gcgggcctct tcgctattac gccagcccaa gctaccatga

4921 taagtaagta atattaaggt acgggaggta cttggagcgg ccgcaataaa atatctttat

4981 tttcattaca tctgtgtgtt ggttttttgt gtgaatcgat agtactaaca tacgctctcc

5041 atcaaaacaa aacgaaacaa aacaaactag caaaataggc tgtccccagt gcaagtgcag

5101 gtgccagaac atttctctat cgata

//

**ST04.2:**

LOCUS ST04.2 3499 bp DNA circular SYN 07-JUL-2021

DEFINITION synthetic circular DNA

ACCESSION .

VERSION .

KEYWORDS .

SOURCE synthetic DNA construct

ORGANISM synthetic DNA construct

REFERENCE 1 (bases 1 to 3499)

AUTHORS .

TITLE .

JOURNAL .

FEATURES Location/Qualifiers

source 1..3499

/organism="synthetic DNA construct"

/mol_type="other DNA"

polyA_signal 3..51

/note="synthetic polyadenylation signal"

misc_feature 65..156

/label=pause site

/note="RNA polymerase II transcriptional pause signal from the human alpha-2 globin gene"

protein_bind 246..340

/label=5X UAS

/bound_moiety="GAL4"

/note="five tandem copies of the ""ScaI site"" 17-mer

CGGAGTACTGTCCTCCG, an upstream activating sequence (UAS) that efficiently binds yeast Gal4 (Webster et al., 1988;

Pfeiffer et al., 2010)"

promoter 341..455

/label=Minimal promoter

regulatory 550..559

/regulatory_class="other"

/label=Kozak sequence

/note="vertebrate consensus sequence for strong initiation of translation (Kozak, 1987)"

CDS 556..1068

/codon_start=1

/product="NanoLuc(R) luciferase"

/label=Nluc

/note="human codon-optimized"

/translation="MVFTLEDFVGDWRQTAGYNLDQVLEQGGVSSLFQNLGVSVTPIQR IVLSGENGLKIDIHVIIPYEGLSGDQMGQIEKIFKVVYPVDDHHFKVILHYGTLVIDGV TPNMIDYFGRPYEGIAVFDGKKITVTGTLWNGNKIIDERLINPDGSLLFRVTINGVTGW RLCERILA"

CDS 1072..1191

/codon_start=1

/product="PEST degradation sequence from mouse ornithine decarboxylase"

/label=hPEST

/note="human codon-optimized"

/translation="SHGFPPEVEEQAAGTLPMSCAQESGMDRHPAACASARINV"

polyA_signal 1219..1340

/label=SV40 poly(A) signal

/note="SV40 polyadenylation signal"

rep_origin complement(1749..2337)

/direction=LEFT

/label=ori

/note="high-copy-number ColE1/pMB1/pBR322/pUC origin of

replication"

CDS complement(2537..3397)

/codon_start=1

/product="beta-lactamase"

/label=AmpR

/note="confers resistance to ampicillin, carbenicillin, and related antibiotics"

/translation="MSIQHFRVALIPFFAAFCLPVFAHPETLVKVKDAEDQLGARVGYI ELDLNSGKILESFRPEERFPMMSTFKVLLCGAVLSRIDAGQEQLGRRIHYSQNDLVEYS PVTEKHLTDGMTVRELCSAAITMSDNTAANLLLTTIGGPKELTAFLHNMGDHVTRLDRW EPELNEAIPNDERDTTMPVAMATTLRKLLTGELLTLASRQQLIDWMEADKVAGPLLRSA LPAGWFIADKSGAGERGSRGIIAALGPDGKPSRIVVIYTTGSQATMDERNRQIAEIGAS LIKHW"

ORIGIN

1 gcaataaaat atctttattt tcattacatc tgtgtgttgg ttttttgtgt gaatcgatag

61 tactaacata cgctctccat caaaacaaaa cgaaacaaaa caaactagca aaataggctg

121 tccccagtgc aagtgcaggt gccagaacat ttctctggcc taactggccg gtacctgagc

181 tcgctagcct cgaggataat tcctcgacgg atctgcgatc taagtaagct tgcatgcctg

241 caggtcggag tactgtcctc cgagcggagt actgtcctcc gagcggagta ctgtcctccg

301 agcggagtac tgtcctccga gcggagtact gtcctccgag cggagactct agcgagatac

361 atgtcgtcga ccttgggcat aaaaggcaga gcactgcagc tgctgcttac acttgctttt

421 gacacaactg tgtttacttg caatccccca agcttcacat atgcatgcac tagtggcgcc

481 tgtcgacgcg tagaattatc aagatctggc ctcggcggcc aagcttggca atccggtact

541 gttggtaaag ccaccatggt cttcacactc gaagatttcg ttggggactg gcgacagaca

601 gccggctaca acctggacca agtccttgaa cagggaggtg tgtccagttt gtttcagaat

661 ctcggggtgt ccgtaactcc gatccaaagg attgtcctga gcggtgaaaa tgggctgaag

721 atcgacatcc atgtcatcat cccgtatgaa ggtctgagcg gcgaccaaat gggccagatc

781 gaaaaaattt ttaaggtggt gtaccctgtg gatgatcatc actttaaggt gatcctgcac

841 tatggcacac tggtaatcga cggggttacg ccgaacatga tcgactattt cggacggccg

901 tatgaaggca tcgccgtgtt cgacggcaaa aagatcactg taacagggac cctgtggaac

961 ggcaacaaaa ttatcgacga gcgcctgatc aaccccgacg gctccctgct gttccgagta

1021 accatcaacg gagtgaccgg ctggcggctg tgcgaacgca ttctggcgaa ttctcacggc

1081 tttccgcctg aggttgaaga gcaagccgcc ggtacattgc ctatgtcctg cgcacaagaa

1141 agcggtatgg accggcaccc agccgcttgt gcttcagctc gcatcaacgt ctaaggccgc

1201 gactctagac agacatgata agatacattg atgagtttgg acaaaccaca actagaatgc

1261 agtgaaaaaa atgctttatt tgtgaaattt gtgatgctat tgctttattt gtaaccatta

1321 taagctgcaa taaacaagtt aacaacaaca attgcattca ttttatgttt caggttcagg

1381 gggaggtgtg ggaggttttt taaagcaagt aaaacctcta caaatgtggt aggatccgtc

1441 gaccgatgcc cttgagagcc ttcaacccag tcagctcctt ccggtgggcg cggggcatga

1501 ctatcgtcgc cgcacttatg actgtcttct ttatcatgca actcgtagga caggtgccgg

1561 cagcgctctt ccgcttcctc gctcactgac tcgctgcgct cggtcgttcg gctgcggcga

1621 gcggtatcag ctcactcaaa ggcggtaata cggttatcca cagaatcagg ggataacgca

1681 ggaaagaaca tgtgagcaaa aggccagcaa aaggccagga accgtaaaaa ggccgcgttg

1741 ctggcgtttt tccataggct ccgcccccct gacgagcatc acaaaaatcg acgctcaagt

1801 cagaggtggc gaaacccgac aggactataa agataccagg cgtttccccc tggaagctcc

1861 ctcgtgcgct ctcctgttcc gaccctgccg cttaccggat acctgtccgc ctttctccct

1921 tcgggaagcg tggcgctttc tcatagctca cgctgtaggt atctcagttc ggtgtaggtc

1981 gttcgctcca agctgggctg tgtgcacgaa ccccccgttc agcccgaccg ctgcgcctta

2041 tccggtaact atcgtcttga gtccaacccg gtaagacacg acttatcgcc actggcagca

2101 gccactggta acaggattag cagagcgagg tatgtaggcg gtgctacaga gttcttgaag

2161 tggtggccta actacggcta cactagaaga acagtatttg gtatctgcgc tctgctgaag

2221 ccagttacct tcggaaaaag agttggtagc tcttgatccg gcaaacaaac caccgctggt

2281 agcggtggtt tttttgtttg caagcagcag attacgcgca gaaaaaaagg atctcaagaa

2341 gatcctttga tcttttctac ggggtctgac gctcagtgga acgaaaactc acgttaaggg

2401 attttggtca tgagattatc aaaaaggatc ttcacctaga tccttttaaa ttaaaaatga

2461 agttttaaat caatctaaag tatatatgag taaacttggt ctgacagcgg ccgcaaatgc

2521 taaaccactg cagtggttac cagtgcttga tcagtgaggc accgatctca gcgatctgcc

2581 tatttcgttc gtccatagtg gcctgactcc ccgtcgtgta gatcactacg attcgtgagg

2641 gcttaccatc aggccccagc gcagcaatga tgccgcgaga gccgcgttca ccggcccccg

2701 atttgtcagc aatgaaccag ccagcaggga gggccgagcg aagaagtggt cctgctactt

2761 tgtccgcctc catccagtct atgagctgct gtcgtgatgc tagagtaaga agttcgccag

2821 tgagtagttt ccgaagagtt gtggccattg ctactggcat cgtggtatca cgctcgtcgt

2881 tcggtatggc ttcgttcaac tctggttccc agcggtcaag ccgggtcaca tgatcaccca

2941 tattatgaag aaatgcagtc agctccttag ggcctccgat cgttgtcaga agtaagttgg

3001 ccgcggtgtt gtcgctcatg gtaatggcag cactacacaa ttctcttacc gtcatgccat

3061 ccgtaagatg cttttccgtg accggcgagt actcaaccaa gtcgttttgt gagtagtgta

3121 tacggcgacc aagctgctct tgcccggcgt ctatacggga caacaccgcg ccacatagca

3181 gtactttgaa agtgctcatc atcgggaatc gttcttcggg gcggaaagac tcaaggatct

3241 tgccgctatt gagatccagt tcgatatagc ccactcttgc acccagttga tcttcagcat

3301 cttttacttt caccagcgtt tcggggtgtg caaaaacagg caagcaaaat gccgcaaaga

3361 agggaatgag tgcgacacga aaatgttgga tgctcatact cgtccttttt caatattatt

3421 gaagcattta tcagggttac tagtacgtct ctcaaggata agtaagtaat attaaggtac

3481 gggaggtatt ggacaggcc

//

**ST04.4:**

LOCUS ST04.4 5081 bp DNA circular SYN 07-JUL-2021

DEFINITION synthetic circular DNA

ACCESSION .

VERSION .

KEYWORDS .

SOURCE synthetic DNA construct

ORGANISM synthetic DNA construct

REFERENCE 1 (bases 1 to 5081)

AUTHORS .

TITLE .

JOURNAL .

FEATURES Location/Qualifiers

source 1..5081

/organism="synthetic DNA construct"

/mol_type="other DNA"

misc_feature 24..148

/label=GAL4 UAS

promoter 161..173

/label=E1b TATA

intron 329..446

/label=IVS8 sysnthetic intron

regulatory 482..491

/regulatory_class="other"

/label=Kozak sequence

/note="vertebrate consensus sequence for strong initiation of translation (Kozak, 1987)"

CDS 488..1045

/codon_start=1

/product="secreted Gaussia luciferase"

/label=hGLuc

/note="human codon-optimized"

/translation="MGVKVLFALICIAVAEAKPTENNEDFNIVAVASNFATTDLDADRG KLPGKKLPLEVLKEMEANARKAGCTRGCLICLSHIKCTPKMKKFIPGRCHTYEGDKESA QGGIGEAIVDIPEIPGFKDLEPMEQFIAQVDLCVDCTTGCLKGLANVQCSDLLKKWLPQ RCATFASKIQGQVDKIKGAGGD"

polyA_signal 1079..1303

/label=bGH poly(A) signal

/note="bovine growth hormone polyadenylation signal"

rep_origin 1349..1777

/direction=RIGHT

/label=f1 ori

/note="f1 bacteriophage origin of replication; arrow

indicates direction of (+) strand synthesis"

promoter 1791..2120

/label=SV40 promoter

/note="SV40 enhancer and early promoter"

rep_origin 1971..2106

/label=SV40 ori

/note="SV40 origin of replication"

promoter 2168..2215

/label=EM7 promoter

/note="synthetic bacterial promoter "

CDS 2234..2608

/codon_start=1

/gene="Sh ble from Streptoalloteichus hindustanus"

/product="antibiotic-binding protein"

/label=BleoR

/note="confers resistance to bleomycin, phleomycin, and

Zeocin(TM)"

/translation="MAKLTSAVPVLTARDVAGAVEFWTDRLGFSRDFVEDDFAGVVRDD VTLFISAVQDQVVPDNTLAWVWVRGLDELYAEWSEVVSTNFRDASGPAMTEIGEQPWGR EFALRDPAGNCVHFVAEEQD"

polyA_signal 2738..2859

/label=SV40 poly(A) signal

/note="SV40 polyadenylation signal"

primer_bind complement(2908..2924)

/label=M13 rev

/note="common sequencing primer, one of multiple similar variants"

protein_bind 2932..2948

/label=lac operator

/bound_moiety="lac repressor encoded by lacI"

/note="The lac repressor binds to the lac operator to

inhibit transcription in E. coli. This inhibition can be relieved by adding lactose or

isopropyl-beta-D-thiogalactopyranoside (IPTG)."

promoter complement(2956..2986)

/label=lac promoter

/note="promoter for the E. coli lac operon"

protein_bind 3001..3022

/label=CAP binding site

/bound_moiety="E. coli catabolite activator protein"

/note="CAP binding activates transcription in the presence of cAMP."

rep_origin complement(3310..3895)

/direction=LEFT

/label=ori

/note="high-copy-number ColE1/pMB1/pBR322/pUC origin of

replication"

CDS complement(4066..4926)

/codon_start=1

/gene="bla"

/product="beta-lactamase"

/label=AmpR

/note="confers resistance to ampicillin, carbenicillin, and related antibiotics"

/translation="MSIQHFRVALIPFFAAFCLPVFAHPETLVKVKDAEDQLGARVGYI ELDLNSGKILESFRPEERFPMMSTFKVLLCGAVLSRIDAGQEQLGRRIHYSQNDLVEYS PVTEKHLTDGMTVRELCSAAITMSDNTAANLLLTTIGGPKELTAFLHNMGDHVTRLDRW EPELNEAIPNDERDTTMPVAMATTLRKLLTGELLTLASRQQLIDWMEADKVAGPLLRSA LPAGWFIADKSGAGERGSRGIIAALGPDGKPSRIVVIYATGSQATMDERNRQIAEIGAS LIKHW"

promoter complement(4927..5031)

/gene="bla"

/label=AmpR promoter

ORIGIN

1 ccgagctctt acgcgggtcg aagcggagta ctgtcctccg agtggagtac tgtcctccga

61 gcggagtact gtcctccgag tcgagggtcg aagcggagta ctgtcctccg agtggagtac

121 tgtcctccga gcggagtact gtcctccgag tcgactctag agggtatata atggatctcg

181 agatatcgga gctcgtttag tgaaccgtca gatcgcctgg agacgccatc cacgctgttt

241 tgacctccat agaagacacc gggaccgatc cagcctccgc ggccgggaac ggtgcattgg

301 aacgcgcatt ccccgtgtta attaacaggt aagtgtcttc ctcctgtttc cttcccctgc

361 tattctgctc aaccttccta tcagaaactg cagtatctgt atttttgcta gcagtaatac

421 taacggttct ttttttctct tcacaggcca ccaagcttgg caatccggta ctgttggtaa

481 agccaccatg ggagtcaaag ttctgtttgc cctgatctgc atcgctgtgg ccgaggccaa

541 gcccaccgag aacaacgaag acttcaacat cgtggccgtg gccagcaact tcgcgaccac

601 ggatctcgat gctgaccgcg ggaagttgcc cggcaagaag ctgccgctgg aggtgctcaa

661 agagatggaa gccaatgccc ggaaagctgg ctgcaccagg ggctgtctga tctgcctgtc

721 ccacatcaag tgcacgccca agatgaagaa gttcatccca ggacgctgcc acacctacga

781 aggcgacaaa gagtccgcac agggcggcat aggcgaggcg atcgtcgaca ttcctgagat

841 tcctgggttc aaggacttgg agcctatgga gcagttcatc gcacaggtcg atctgtgtgt

901 ggactgcaca actggctgcc tcaaagggct tgccaacgtg cagtgttctg acctgctcaa

961 gaagtggctg ccgcaacgct gtgcgacctt tgccagcaag atccagggcc aggtggacaa

1021 gatcaagggg gccggtggtg actaagcggc cgctcgaaaa cccgctgatc agcctcgact

1081 gtgccttcta gttgccagcc atctgttgtt tgcccctccc ccgtgccttc cttgaccctg

1141 gaaggtgcca ctcccactgt cctttcctaa taaaatgagg aaattgcatc gcattgtctg

1201 agtaggtgtc attctattct ggggggtggg gtggggcagg acagcaaggg ggaggattgg

1261 gaagacaata gcaggcatgc tggggatgcg gtgggctcta tggcttctga ggcggaaaga

1321 accagctggg gctctagggg gtatccccac gcgccctgta gcggcgcatt aagcgcggcg

1381 ggtgtggtgg ttacgcgcag cgtgaccgct acacttgcca gcgccctagc gcccgctcct

1441 ttcgctttct tcccttcctt tctcgccacg ttcgccggct ttccccgtca agctctaaat

1501 cgggggctcc ctttagggtt ccgatttagt gctttacggc acctcgaccc caaaaaactt

1561 gattagggtg atggttcacg tagtgggcca tcgccctgat agacggtttt tcgccctttg

1621 acgttggagt ccacgttctt taatagtgga ctcttgttcc aaactggaac aacactcaac

1681 cctatctcgg tctattcttt tgatttataa gggattttgc cgatttcggc ctattggtta

1741 aaaaatgagc tgatttaaca aaaatttaac gcgaattaat tctgtggaat gtgtgtcagt

1801 tagggtgtgg aaagtcccca ggctccccag caggcagaag tatgcaaagc atgcatctca

1861 attagtcagc aaccaggtgt ggaaagtccc caggctcccc agcaggcaga agtatgcaaa

1921 gcatgcatct caattagtca gcaaccatag tcccgcccct aactccgccc atcccgcccc

1981 taactccgcc cagttccgcc cattctccgc cccatggctg actaattttt tttatttatg

2041 cagaggccga ggccgcctct gcctctgagc tattccagaa gtagtgagga ggcttttttg

2101 gaggcctagg cttttgcaaa aagctcccgg gagcttgtat atccattttc ggatctgatc

2161 agcacgtgtt gacaattaat catcggcata gtatatcggc atagtataat acgacaaggt

2221 gaggaactaa accatggcca agttgaccag tgccgttccg gtgctcaccg cgcgcgacgt

2281 cgccggagcg gtcgagttct ggaccgaccg gctcgggttc tcccgggact tcgtggagga

2341 cgacttcgcc ggtgtggtcc gggacgacgt gaccctgttc atcagcgcgg tccaggacca

2401 ggtggtgccg gacaacaccc tggcctgggt gtgggtgcgc ggcctggacg agctgtacgc

2461 cgagtggtcg gaggtcgtgt ccacgaactt ccgggacgcc tccgggccgg ccatgaccga

2521 gatcggcgag cagccgtggg ggcgggagtt cgccctgcgc gacccggccg gcaactgcgt

2581 gcacttcgtg gccgaggagc aggactgaca cgtgctacga gatttcgatt ccaccgccgc

2641 cttctatgaa aggttgggct tcggaatcgt tttccgggac gccggctgga tgatcctcca

2701 gcgcggggat ctcatgctgg agttcttcgc ccaccccaac ttgtttattg cagcttataa

2761 tggttacaaa taaagcaata gcatcacaaa tttcacaaat aaagcatttt tttcactgca

2821 ttctagttgt ggtttgtcca aactcatcaa tgtatcttat catgtctgta taccgtcgac

2881 atctagctag agcttggcgt aatcatggtc atagctgttt cctgtgtgaa attgttatcc

2941 gctcacaatt ccacacaaca tacgagccgg aagcataaag tgtaaagcct ggggtgccta

3001 atgagtgagc taactcacat taattgcgtt gcgctcactg cccgctttcc agtcgggaaa

3061 cctgtcgtgc cagctgcatt aatgaatcgg ccaacgcgcg gggagaggcg gtttgcgtat

3121 tgggcgctct tccgcttcct cgctcactga ctcgctgcgc tcggtcgttc ggctgcggcg

3181 agcggtatca gctcactcaa aggcggtaat acggttatcc acagaatcag gggataacgc

3241 aggaaagaac atgtgagcaa aaggccagca aaaggccagg aaccgtaaaa aggccgcgtt

3301 gctggcgttt ttccataggc tccgcccccc tgacgagcat cacaaaaatc gacgctcaag

3361 tcagaggtgg cgaaacccga caggactata aagataccag gcgtttcccc ctggaagctc

3421 cctcgtgcgc tctcctgttc cgaccctgcc gcttaccgga tacctgtccg cctttctccc

3481 ttcgggaagc gtggcgcttt ctcatagctc acgctgtagg tatctcagtt cggtgtaggt

3541 cgttcgctcc aagctgggct gtgtgcacga accccccgtt cagcccgacc gctgcgcctt

3601 atccggtaac tatcgtcttg agtccaaccc ggtaagacac gacttatcgc cactggcagc

3661 agccactggt aacaggatta gcagagcgag gtatgtaggc ggtgctacag agttcttgaa

3721 gtggtggcct aactacggct acactagaag aacagtattt ggtatctgcg ctctgctgaa

3781 gccagttacc ttcggaaaaa gagttggtag ctcttgatcc ggcaaacaaa ccaccgctgg

3841 tagcggtttt tttgtttgca agcagcagat tacgcgcaga aaaaaaggat ctcaagaaga

3901 tcctttgatc ttttctacgg ggtctgacgc tcagtggaac gaaaactcac gttaagggat

3961 tttggtcatg agattatcaa aaaggatctt cacctagatc cttttaaatt aaaaatgaag

4021 ttttaaatca atctaaagta tatatgagta aacttggtct gacagttacc aatgcttaat

4081 cagtgaggca cctatctcag cgatctgtct atttcgttca tccatagttg cctgactccc

4141 cgtcgcgtag ataactacga tacgggaggg cttaccatct ggccccagtg ctgcaatgat

4201 accgcgagac ccacgctcac cggctccaga tttatcagca ataaaccagc cagccggaag

4261 ggccgagcgc agaagtggtc ctgcaacttt atccgcctcc atccagtcta ttaattgttg

4321 ccgggaagct agagtaagta gttcgccagt taatagtttg cgcaacgttg ttgccattgc

4381 tacaggcatc gtggtgtcac gctcgtcgtt tggtatggct tcattcagct ccggttccca

4441 acgatcaagg cgagttacat gatcccccat gttgtgcaaa aaagcggtta gctccttcgg

4501 tcctccgatc gttgtcagaa gtaagttggc cgcagtgtta tcactcatgg ttatggcagc

4561 actgcataat tctcttactg tcatgccatc cgtaagatgc ttttctgtga ctggtgagta

4621 ctcaaccaag tcattctgag aatagtgtat gcggcgaccg agttgctctt gcccggcgtc

4681 aatacgggat aataccgcgc cacatagcag aactttaaaa gtgctcatca ttggaaaacg

4741 ttcttcgggg cgaaaactct caaggatctt accgctgttg agatccagtt cgatgtaacc

4801 cactcgtgca cccaactgat cttcagcatc ttttactttc accagcgttt ctgggtgagc

4861 aaaaacagga aggcaaaatg ccgcaaaaaa gggaataagg gcgacacgga aatgttgaat

4921 actcatactc ttcctttttc aatattattg aagcatttat cagggttatt gtctcatgag

4981 cggatacata tttgaatgta tttagaaaaa taaacaaata ggggttccgc gcacatttcc

5041 ccgaaaagtg ccacctgacg tcgacggatc gggagatcgt a

//

**ST04.5:**

LOCUS ST04.5 5164 bp DNA circular SYN 09-JUL-2021

DEFINITION synthetic circular DNA

ACCESSION .

VERSION .

KEYWORDS .

SOURCE synthetic DNA construct

ORGANISM synthetic DNA construct

REFERENCE 1 (bases 1 to 5164)

AUTHORS .

TITLE .

JOURNAL .

FEATURES Location/Qualifiers

source 1..5164

/organism="synthetic DNA construct"

/mol_type="other DNA"

misc_feature 24..148

/label=GAL4 UAS

promoter 161..173

/label=E1b TATA

intron 329..446

/label=IVS8 synthetic intron

regulatory 482..491

/regulatory_class="other"

/label=Kozak sequence

/note="vertebrate consensus sequence for strong initiation of translation (Kozak, 1987)"

CDS 488..1000

/codon_start=1

/product="NanoLuc(R) luciferase"

/label=Nluc

/note="human codon-optimized"

/translation="MVFTLEDFVGDWRQTAGYNLDQVLEQGGVSSLFQNLGVSVTPIQR IVLSGENGLKIDIHVIIPYEGLSGDQMGQIEKIFKVVYPVDDHHFKVILHYGTLVIDGV TPNMIDYFGRPYEGIAVFDGKKITVTGTLWNGNKIIDERLINPDGSLLFRVTINGVTGW RLCERILA"

CDS 1004..1123

/codon_start=1

/product="PEST degradation sequence from mouse ornithine decarboxylase"

/label=hPEST

/note="human codon-optimized"

/translation="SHGFPPEVEEQAAGTLPMSCAQESGMDRHPAACASARINV"

polyA_signal 1162..1386

/label=bGH poly(A) signal

/note="bovine growth hormone polyadenylation signal"

rep_origin 1432..1860

/direction=RIGHT

/label=f1 ori

/note="f1 bacteriophage origin of replication; arrow

indicates direction of (+) strand synthesis"

promoter 1874..2203

/label=SV40 promoter

/note="SV40 enhancer and early promoter"

rep_origin 2054..2189

/label=SV40 ori

/note="SV40 origin of replication"

promoter 2251..2298

/label=EM7 promoter

/note="synthetic bacterial promoter "

CDS 2317..2691

/codon_start=1

/gene="Sh ble from Streptoalloteichus hindustanus"

/product="antibiotic-binding protein"

/label=BleoR

/note="confers resistance to bleomycin, phleomycin, and

Zeocin(TM)"

/translation="MAKLTSAVPVLTARDVAGAVEFWTDRLGFSRDFVEDDFAGVVRDD VTLFISAVQDQVVPDNTLAWVWVRGLDELYAEWSEVVSTNFRDASGPAMTEIGEQPWGR EFALRDPAGNCVHFVAEEQD"

polyA_signal 2821..2942

/label=SV40 poly(A) signal

/note="SV40 polyadenylation signal"

primer_bind complement(2991..3007)

/label=M13 rev

/note="common sequencing primer, one of multiple similar variants"

protein_bind 3015..3031

/label=lac operator

/bound_moiety="lac repressor encoded by lacI"

/note="The lac repressor binds to the lac operator to

inhibit transcription in E. coli. This inhibition can be relieved by adding lactose or isopropyl-beta-D-thiogalactopyranoside (IPTG)."

promoter complement(3039..3069)

/label=lac promoter

/note="promoter for the E. coli lac operon"

protein_bind 3084..3105

/label=CAP binding site

/bound_moiety="E. coli catabolite activator protein"

/note="CAP binding activates transcription in the presence of cAMP."

rep_origin complement(3393..3978)

/direction=LEFT

/label=ori

/note="high-copy-number ColE1/pMB1/pBR322/pUC origin of

replication"

CDS complement(4149..5009)

/codon_start=1

/gene="bla"

/product="beta-lactamase"

/label=AmpR

/note="confers resistance to ampicillin, carbenicillin, and related antibiotics"

/translation="MSIQHFRVALIPFFAAFCLPVFAHPETLVKVKDAEDQLGARVGYI ELDLNSGKILESFRPEERFPMMSTFKVLLCGAVLSRIDAGQEQLGRRIHYSQNDLVEYS PVTEKHLTDGMTVRELCSAAITMSDNTAANLLLTTIGGPKELTAFLHNMGDHVTRLDRW EPELNEAIPNDERDTTMPVAMATTLRKLLTGELLTLASRQQLIDWMEADKVAGPLLRSA LPAGWFIADKSGAGERGSRGIIAALGPDGKPSRIVVIYATGSQATMDERNRQIAEIGAS LIKHW"

promoter complement(5010..5114)

/gene="bla"

/label=AmpR promoter

ORIGIN

1 ccgagctctt acgcgggtcg aagcggagta ctgtcctccg agtggagtac tgtcctccga

61 gcggagtact gtcctccgag tcgagggtcg aagcggagta ctgtcctccg agtggagtac

121 tgtcctccga gcggagtact gtcctccgag tcgactctag agggtatata atggatctcg

181 agatatcgga gctcgtttag tgaaccgtca gatcgcctgg agacgccatc cacgctgttt

241 tgacctccat agaagacacc gggaccgatc cagcctccgc ggccgggaac ggtgcattgg

301 aacgcgcatt ccccgtgtta attaacaggt aagtgtcttc ctcctgtttc cttcccctgc

361 tattctgctc aaccttccta tcagaaactg cagtatctgt atttttgcta gcagtaatac

421 taacggttct ttttttctct tcacaggcca ccaagcttgg caatccggta ctgttggtaa

481 agccaccatg gtcttcacac tcgaagattt cgttggggac tggcgacaga cagccggcta

541 caacctggac caagtccttg aacagggagg tgtgtccagt ttgtttcaga atctcggggt

601 gtccgtaact ccgatccaaa ggattgtcct gagcggtgaa aatgggctga agatcgacat

661 ccatgtcatc atcccgtatg aaggtctgag cggcgaccaa atgggccaga tcgaaaaaat

721 ttttaaggtg gtgtaccctg tggatgatca tcactttaag gtgatcctgc actatggcac

781 actggtaatc gacggggtta cgccgaacat gatcgactat ttcggacggc cgtatgaagg

841 catcgccgtg ttcgacggca aaaagatcac tgtaacaggg accctgtgga acggcaacaa

901 aattatcgac gagcgcctga tcaaccccga cggctccctg ctgttccgag taaccatcaa

961 cggagtgacc ggctggcggc tgtgcgaacg cattctggcg aattctcacg gctttccgcc

1021 tgaggttgaa gagcaagccg ccggtacatt gcctatgtcc tgcgcacaag aaagcggtat

1081 ggaccggcac ccagccgctt gtgcttcagc tcgcatcaac gtctaaggcc gcgactctag

1141 aaacccgctg atcagcctcg actgtgcctt ctagttgcca gccatctgtt gtttgcccct

1201 cccccgtgcc ttccttgacc ctggaaggtg ccactcccac tgtcctttcc taataaaatg

1261 aggaaattgc atcgcattgt ctgagtaggt gtcattctat tctggggggt ggggtggggc

1321 aggacagcaa gggggaggat tgggaagaca atagcaggca tgctggggat gcggtgggct

1381 ctatggcttc tgaggcggaa agaaccagct ggggctctag ggggtatccc cacgcgccct

1441 gtagcggcgc attaagcgcg gcgggtgtgg tggttacgcg cagcgtgacc gctacacttg

1501 ccagcgccct agcgcccgct cctttcgctt tcttcccttc ctttctcgcc acgttcgccg

1561 gctttccccg tcaagctcta aatcgggggc tccctttagg gttccgattt agtgctttac

1621 ggcacctcga ccccaaaaaa cttgattagg gtgatggttc acgtagtggg ccatcgccct

1681 gatagacggt ttttcgccct ttgacgttgg agtccacgtt ctttaatagt ggactcttgt

1741 tccaaactgg aacaacactc aaccctatct cggtctattc ttttgattta taagggattt

1801 tgccgatttc ggcctattgg ttaaaaaatg agctgattta acaaaaattt aacgcgaatt

1861 aattctgtgg aatgtgtgtc agttagggtg tggaaagtcc ccaggctccc cagcaggcag

1921 aagtatgcaa agcatgcatc tcaattagtc agcaaccagg tgtggaaagt ccccaggctc

1981 cccagcaggc agaagtatgc aaagcatgca tctcaattag tcagcaacca tagtcccgcc

2041 cctaactccg cccatcccgc ccctaactcc gcccagttcc gcccattctc cgccccatgg

2101 ctgactaatt ttttttattt atgcagaggc cgaggccgcc tctgcctctg agctattcca

2161 gaagtagtga ggaggctttt ttggaggcct aggcttttgc aaaaagctcc cgggagcttg

2221 tatatccatt ttcggatctg atcagcacgt gttgacaatt aatcatcggc atagtatatc

2281 ggcatagtat aatacgacaa ggtgaggaac taaaccatgg ccaagttgac cagtgccgtt

2341 ccggtgctca ccgcgcgcga cgtcgccgga gcggtcgagt tctggaccga ccggctcggg

2401 ttctcccggg acttcgtgga ggacgacttc gccggtgtgg tccgggacga cgtgaccctg

2461 ttcatcagcg cggtccagga ccaggtggtg ccggacaaca ccctggcctg ggtgtgggtg

2521 cgcggcctgg acgagctgta cgccgagtgg tcggaggtcg tgtccacgaa cttccgggac

2581 gcctccgggc cggccatgac cgagatcggc gagcagccgt gggggcggga gttcgccctg

2641 cgcgacccgg ccggcaactg cgtgcacttc gtggccgagg agcaggactg acacgtgcta

2701 cgagatttcg attccaccgc cgccttctat gaaaggttgg gcttcggaat cgttttccgg

2761 gacgccggct ggatgatcct ccagcgcggg gatctcatgc tggagttctt cgcccacccc

2821 aacttgttta ttgcagctta taatggttac aaataaagca atagcatcac aaatttcaca

2881 aataaagcat ttttttcact gcattctagt tgtggtttgt ccaaactcat caatgtatct

2941 tatcatgtct gtataccgtc gacatctagc tagagcttgg cgtaatcatg gtcatagctg

3001 tttcctgtgt gaaattgtta tccgctcaca attccacaca acatacgagc cggaagcata

3061 aagtgtaaag cctggggtgc ctaatgagtg agctaactca cattaattgc gttgcgctca

3121 ctgcccgctt tccagtcggg aaacctgtcg tgccagctgc attaatgaat cggccaacgc

3181 gcggggagag gcggtttgcg tattgggcgc tcttccgctt cctcgctcac tgactcgctg

3241 cgctcggtcg ttcggctgcg gcgagcggta tcagctcact caaaggcggt aatacggtta

3301 tccacagaat caggggataa cgcaggaaag aacatgtgag caaaaggcca gcaaaaggcc

3361 aggaaccgta aaaaggccgc gttgctggcg tttttccata ggctccgccc ccctgacgag

3421 catcacaaaa atcgacgctc aagtcagagg tggcgaaacc cgacaggact ataaagatac

3481 caggcgtttc cccctggaag ctccctcgtg cgctctcctg ttccgaccct gccgcttacc

3541 ggatacctgt ccgcctttct cccttcggga agcgtggcgc tttctcatag ctcacgctgt

3601 aggtatctca gttcggtgta ggtcgttcgc tccaagctgg gctgtgtgca cgaacccccc

3661 gttcagcccg accgctgcgc cttatccggt aactatcgtc ttgagtccaa cccggtaaga

3721 cacgacttat cgccactggc agcagccact ggtaacagga ttagcagagc gaggtatgta

3781 ggcggtgcta cagagttctt gaagtggtgg cctaactacg gctacactag aagaacagta

3841 tttggtatct gcgctctgct gaagccagtt accttcggaa aaagagttgg tagctcttga

3901 tccggcaaac aaaccaccgc tggtagcggt ttttttgttt gcaagcagca gattacgcgc

3961 agaaaaaaag gatctcaaga agatcctttg atcttttcta cggggtctga cgctcagtgg

4021 aacgaaaact cacgttaagg gattttggtc atgagattat caaaaaggat cttcacctag

4081 atccttttaa attaaaaatg aagttttaaa tcaatctaaa gtatatatga gtaaacttgg

4141 tctgacagtt accaatgctt aatcagtgag gcacctatct cagcgatctg tctatttcgt

4201 tcatccatag ttgcctgact ccccgtcgcg tagataacta cgatacggga gggcttacca

4261 tctggcccca gtgctgcaat gataccgcga gacccacgct caccggctcc agatttatca

4321 gcaataaacc agccagccgg aagggccgag cgcagaagtg gtcctgcaac tttatccgcc

4381 tccatccagt ctattaattg ttgccgggaa gctagagtaa gtagttcgcc agttaatagt

4441 ttgcgcaacg ttgttgccat tgctacaggc atcgtggtgt cacgctcgtc gtttggtatg

4501 gcttcattca gctccggttc ccaacgatca aggcgagtta catgatcccc catgttgtgc

4561 aaaaaagcgg ttagctcctt cggtcctccg atcgttgtca gaagtaagtt ggccgcagtg

4621 ttatcactca tggttatggc agcactgcat aattctctta ctgtcatgcc atccgtaaga

4681 tgcttttctg tgactggtga gtactcaacc aagtcattct gagaatagtg tatgcggcga

4741 ccgagttgct cttgcccggc gtcaatacgg gataataccg cgccacatag cagaacttta

4801 aaagtgctca tcattggaaa acgttcttcg gggcgaaaac tctcaaggat cttaccgctg

4861 ttgagatcca gttcgatgta acccactcgt gcacccaact gatcttcagc atcttttact

4921 ttcaccagcg tttctgggtg agcaaaaaca ggaaggcaaa atgccgcaaa aaagggaata

4981 agggcgacac ggaaatgttg aatactcata ctcttccttt ttcaatatta ttgaagcatt

5041 tatcagggtt attgtctcat gagcggatac atatttgaat gtatttagaa aaataaacaa

5101 ataggggttc cgcgcacatt tccccgaaaa gtgccacctg acgtcgacgg atcgggagat

5161 cgta

//
